# Correction: Single amino-acid mutation in a *Drosoph ila melanogaster* ribosomal protein: An insight in uL11 transcriptional activity

**DOI:** 10.1371/journal.pone.0304237

**Published:** 2024-05-17

**Authors:** Héloïse Grunchec, Jérôme Deraze, Delphine Dardalhon-Cuménal, Valérie Ribeiro, Anne Coléno-Costes, Karine Dias, Sébastien Bloyer, Emmanuèle Mouchel-Vielh, Frédérique Peronnet, Hélène Thomassin

The word "Drosophila" is misspelled in the article title. The correct title is: Single amino-acid mutation in a *Drosophila melanogaster* ribosomal protein: An insight in uL11 transcriptional activity. The correct citation is: Grunchec H, Deraze J, Dardalhon-Cuménal D, Ribeiro V, Coléno-Costes A, Dias K, et al. (2022) Single amino-acid mutation in a *Drosophila melanogaster* ribosomal protein: An insight in uL11 transcriptional activity. PLoS ONE 17(8): e0273198. https://doi.org/10.1371/journal.pone.0273198.
