# Supplementary figures and images for: Single amino-acid mutation in a Drosoph ila melanogaster ribosomal protein: An insight in uL11 transcriptional activity
Source: PLoS One. 2022 Aug 18;17(8):e0273198. doi: 10.1371/journal.pone.0273198 (PMC9387862; doi:10.1371/journal.pone.0273198)

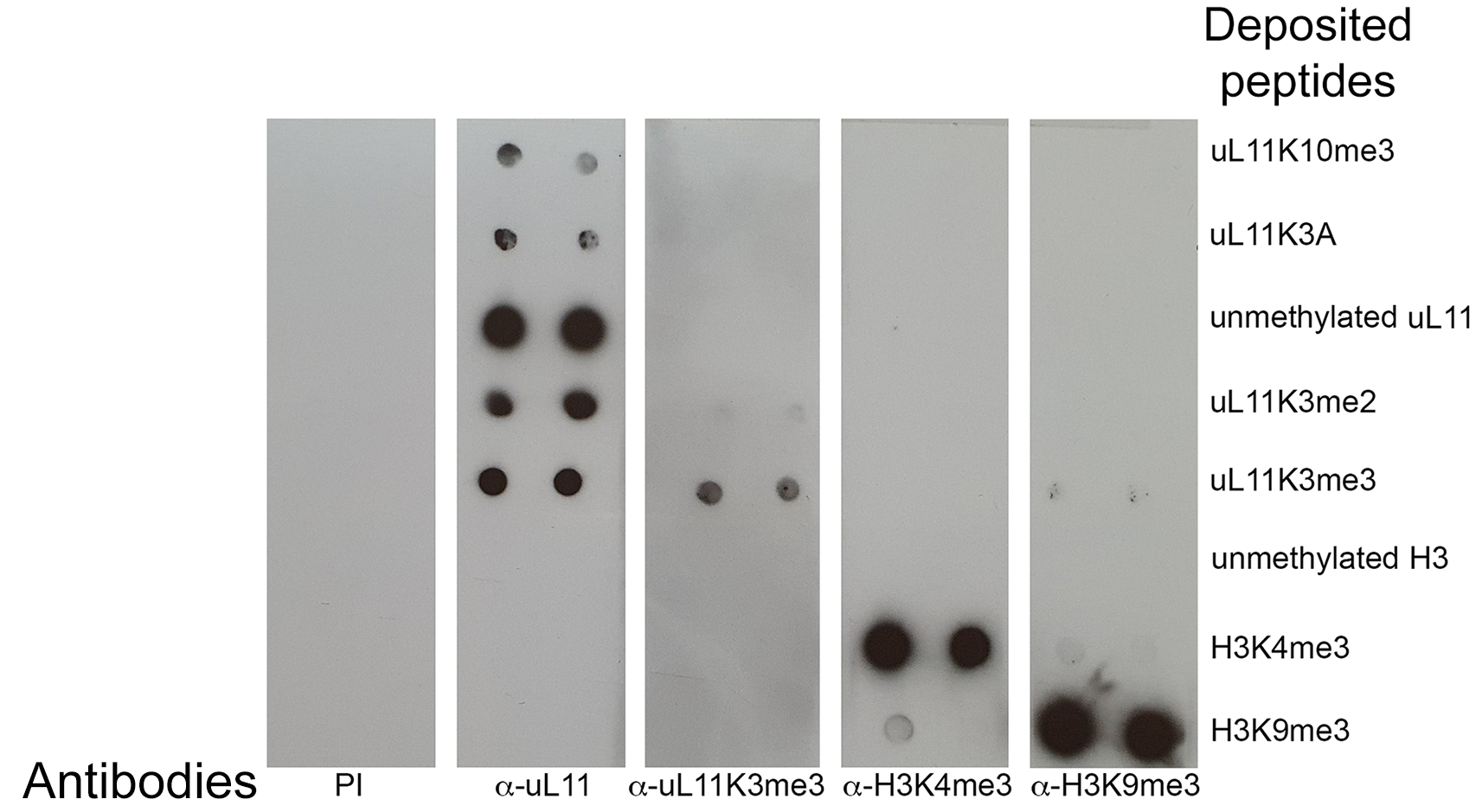

Supplement: S1 Fig — 0.2 (left) and 0.05 μg (right) of each peptide were deposited on a nitrocellulose membrane. Membranes were then incubated with the indicated primary antibodies. Secondary antibodies were as described in Materials and Methods. Peptides: unmethylated uL11, uL11K10me3, uL11K3A, uL11K3me2, and uL11K3me3 peptides were synthesized at the proteomic platform of the Institute of Biology Paris Seine; H3K4me3 and H3K9me3 peptides were from Diagenode, C16000003 and C160000056, respectively. Antibodies: PI: rabbit preimmun serum; α-uL11: 1/14000, described in Materials and Methods; α-uL11K3me3: 1/10000, described in Materials and Methods; α-H3K4me3: 1/1000, Diagenode C15310003; α-H3K9me3: 1/1000, Diagenode C15100146. Secondary antibodies: 1/10000. (TIF) [file pone.0273198.s001.tif]

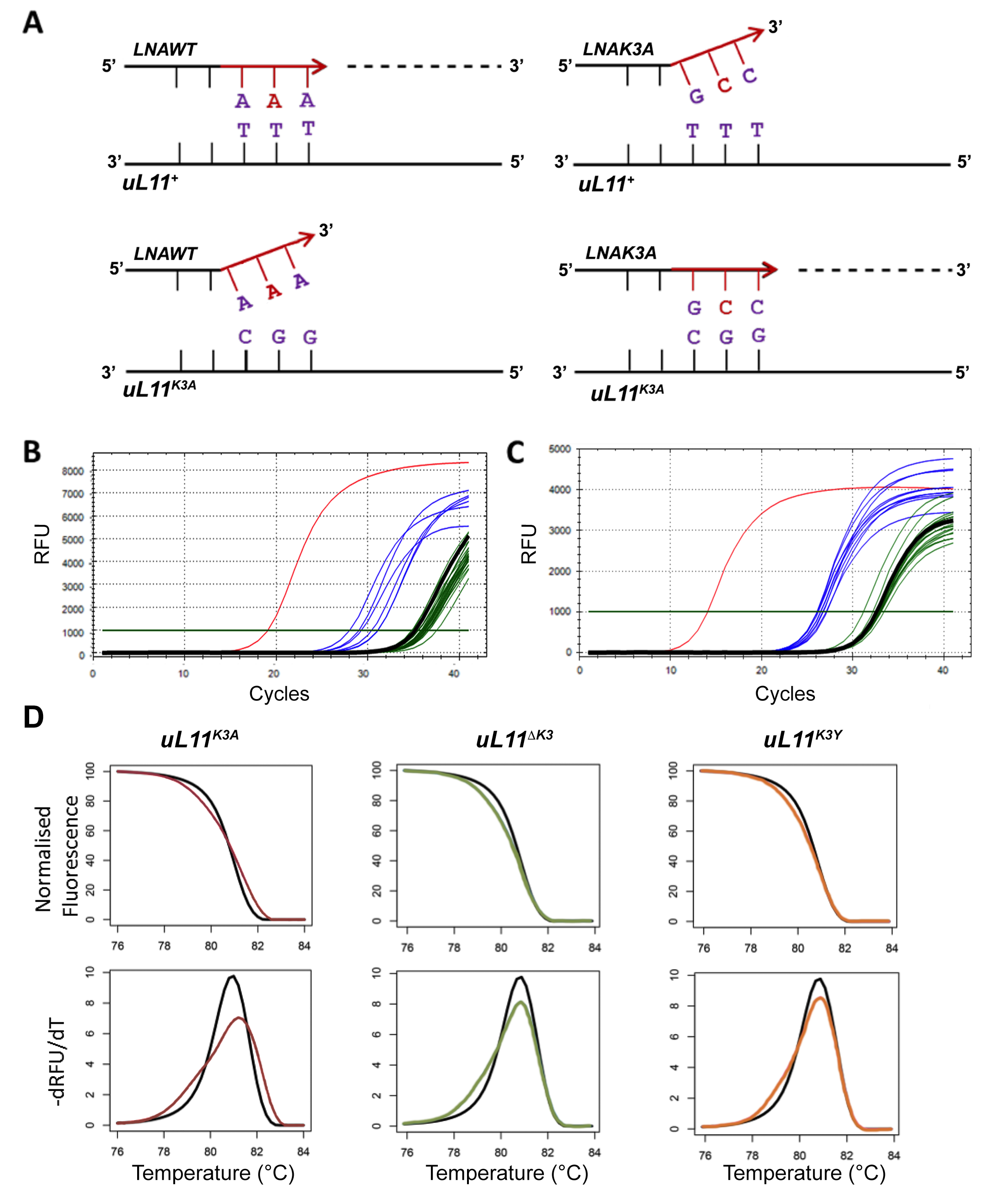

Supplement: S2 Fig — A–Rationale for discriminative PCR. Purple bases correspond to the target codon. Red bases stand for locked nucleic acids (LNA). The LNAWT primer ended with the lysine AAA codon of the wild-type uL11 gene whereas the LNAK3A primer ended with the alanine GCC codon corresponding to the desired mutation. B–qPCRs were performed with the LNAK3A primer matching the uL11K3A allele. Red curve: plasmid carrying the uL11K3A allele as positive control. Black curve: genomic DNA from a wild-type fly. Blue curves: pools of up to 5 different genomic DNAs from candidate G1 flies considered to be positive. Green curves: pools of up to 5 different genomic DNAs from candidate G1 flies considered to be negative. C–The same qPCRs were performed on individual genomic DNAs from the pools that were previously found to be positive for the uL11K3A allele. Several individuals wearing the mutation were thus identified (blue curves). D–High Resolution Melting Analysis (HMRA) of uL11 mutants. Melting profile of the uL11 amplicons from genomic DNAs of G1 flies. Melting peaks flatter and broader than the reference (black) revealed the presence of two different amplicons, indicating that the tested DNA contained a mutation at the uL11 locus. Melting curves were normalized according to the method described by [27]. RFU: Relative Fluorescence Unit. (TIF) [file pone.0273198.s002.tif]

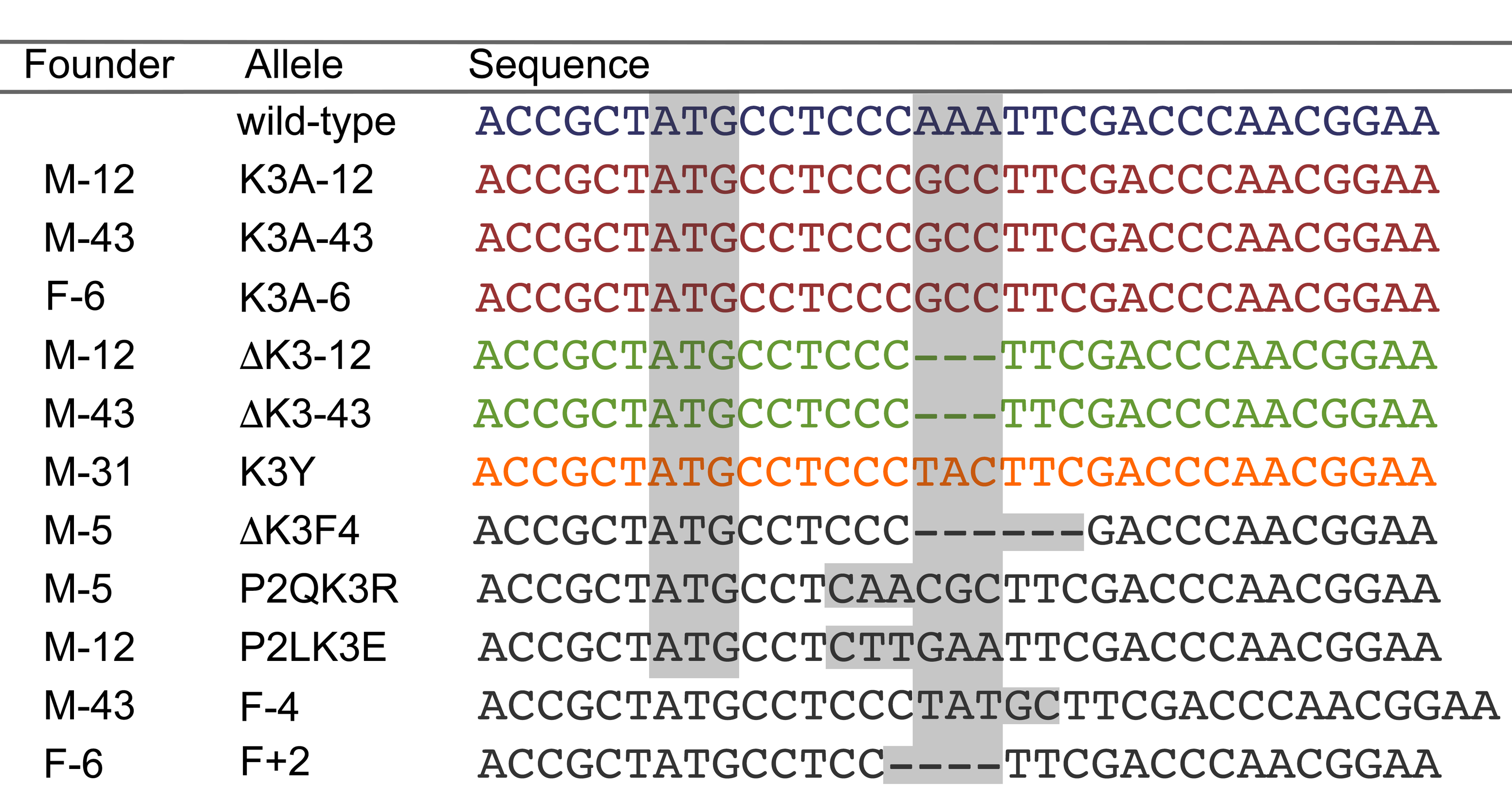

Supplement: S3 Fig — Founder G0 flies were named after their gender (M, male; F, female) and the order of their emergence. Each allele was recovered in several descendants of the same founders. The uL11K3A and uL11ΔK3 alleles were found in the progeny of three and two different founders, respectively. Substitution alleles were named to reflect the amino acid change in the uL11 protein, following the amino acid one letter code. The bottom two alleles were named after the reading frameshift they introduce in the uL11 gene. The wild-type uL11 sequence is provided as reference. The start and the lysine 3 codons of uL11 are highlighted in grey. Mutants F-4 and F+2 introduce a +2 reading frame shift that puts the uL11 CDS in frame with an ATG codon located in the 5’UTR. A protein with a 24 amino acid extension might then be produced. (TIF) [file pone.0273198.s003.tif]

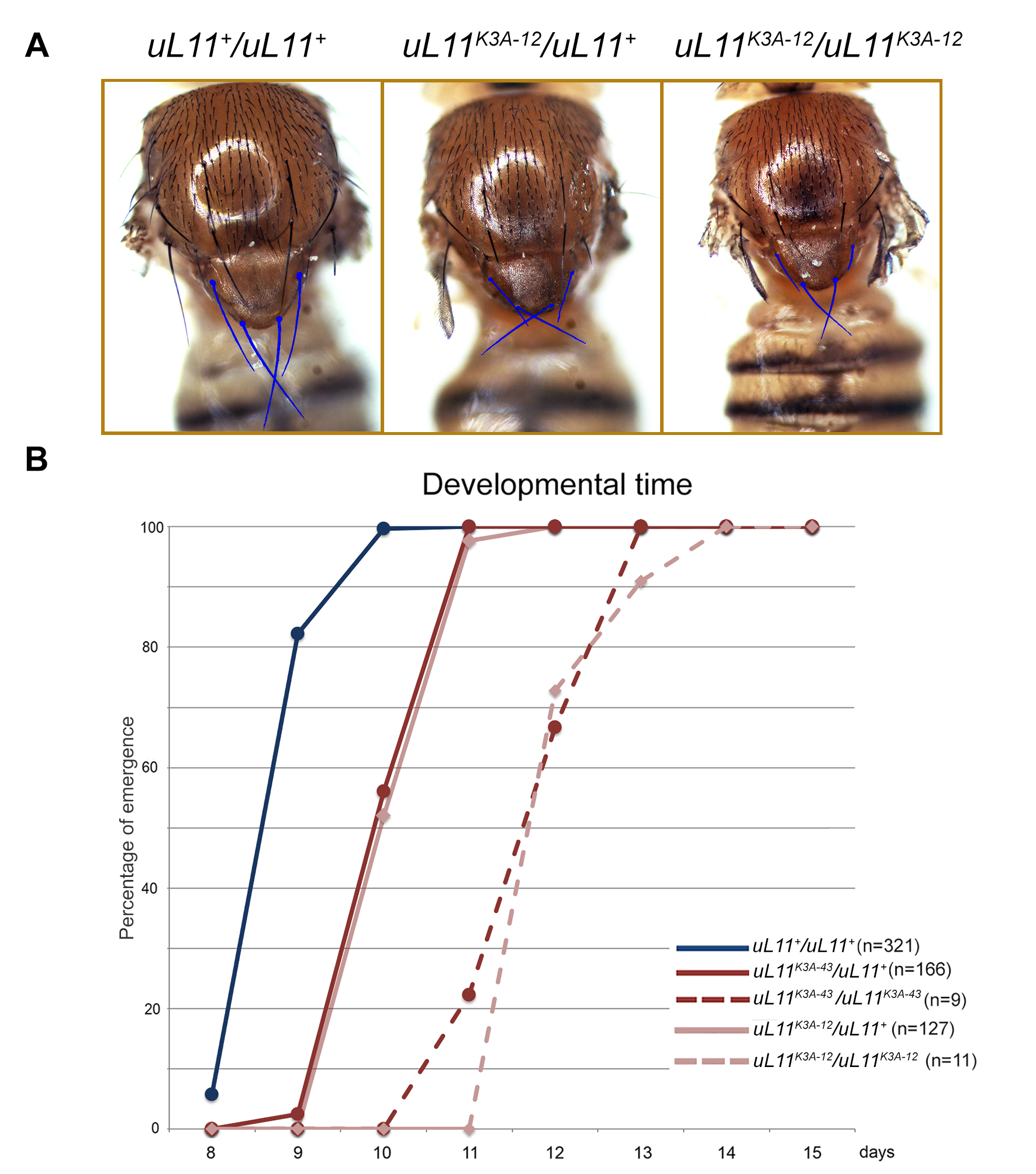

Supplement: S4 Fig — A—From left to right: thorax of a wild-type female, a uL11K3A-12/uL11+ heterozygous female, and a uL11K3A-12/uL11K3A-12 homozygous female. Anterior and posterior scutellar bristles are colorized. B–Developmental time of the uL11K3A-12 mutant as compared to uL11K3A-43—the mutant presented in the main text. The percentage of flies emerged from day 8 to 15 is shown. The total number of flies emerged is indicated in the legend. Solid lines: heterozygous flies; dotted line: homozygous flies; blue: uL11+, burgundy: uL11K3-43, pink: uL11K3A-12. (TIF) [file pone.0273198.s004.tif]

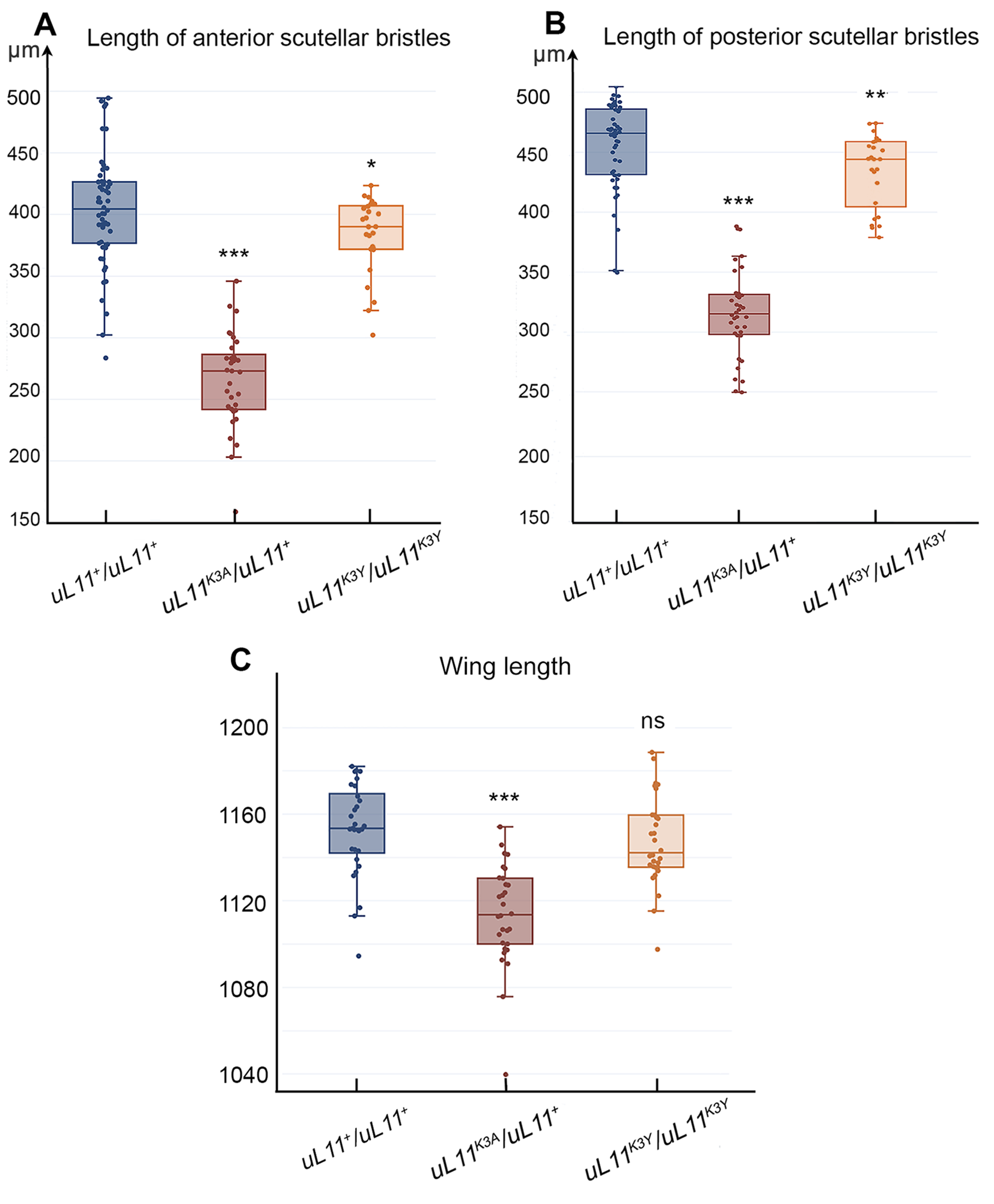

Supplement: S5 Fig — A–Length of anterior scutellar bristles of wild-type females (blue; n = 54), uL11K3A/uL11+ (burgundy, n = 33) and uL11K3Y/uL11K3Y (orange, n = 25). B–Length of posterior scutellar bristles of wild-type males (blue; n = 51), uL11K3A/uL11+ (burgundy, n = 36) and uL11K3Y/uL11K3Y (orange, n = 50). C–Wing size of uL11 wild-type females (blue; n = 29), uL11K3A/uL11+ (burgundy, n = 30) and uL11K3Y/uL11K3Y (orange, n = 25). t-tests: *** p-value < 0.001; ** p-value < 0.01; * p-value < 0.05; ns: non significant. (TIF) [file pone.0273198.s005.tif]
