# Supplementary material for: Single amino-acid mutation in a Drosoph ila melanogaster ribosomal protein: An insight in uL11 transcriptional activity
Source: PLoS One. 2022 Aug 18;17(8):e0273198. doi: 10.1371/journal.pone.0273198 (PMC9387862; doi:10.1371/journal.pone.0273198)
Supplement: S1 Table — In primers LNA-WT and LNA-K3A, uppercase nucleotides correspond to the LNA bases. In primers pho-sgRNA_F and pho-sgRNA_R, uppercase nucleotides correspond to the floating sequences used for cloning. The bold guanosine was introduced to increase efficiency of the U6 promoter. In the ssODN, the complementary ATG and alanine codon sequences are bold and in uppercases, the PAM sequence corresponding to the single guide RNA is in bold. (PDF) [file pone.0273198.s006.pdf]

High Resolution Melting Analysis	
uL11-HRMA_F	5'-tgcggtaaagtacatgagctg-3'
uL11-HRMA_R	5'-tcgaagctcaactcctcaca-3'
LNA Primers	
LNA-WT	5'-gataccgctatgcctcccaAa-3'
LNA-K3A	5'-gataccgctatgcctcccgCc-3'
CRISPR1_R	5'-gaccgaggggaccgatctt-3'
uL11 Sequencing	
uL11-708_F	5'-cgctactgagctttgctacacccc-3'
uL11+805_R	5'-caataacatcgtgagggtgct-3'
Guide RNA	
sgRNA	5′-tccgttgggtcgaatttggg-3'
Cloning of sgRNA in pU6-BbsI-chiRNA	
pho-sgRNA_F	5'-CTTC**G**tccgttgggtcgaatttggg-3'
pho-sgRNA_R	5'-AAACcccaaattcgacccaacggac-3'
Template for fly mutagenesis	
ssODN	5'-agctcaactcctcacaaaaacactcgcttacttacccaatttaacttccgttgggtcg aa**GGC**ggg**aggCAT**agcggtatcttggcttgaacagtcgctgtaaggcaaagattacgtta gttt-3'
uL11^{K3Y} directed mutagenesis	
K3Y_F	5′-caccatgcctccctacttcgacccaacgg-3′
K3Y_R	5′-ccgttgggtcgaagtagggaggcatggtg-3′
qRT-PCR	
Hsp67Bc_F	5′-ctgcttatttgaggctcca-3′
Hsp67Bc_R	5′-ttgatttgcacaccctgggt-3′
GstE6_F	5′-gcccaactttcaccggaata-3′
GstE6_R	5′-tcggcatatttcgagacc-3′
CG13516_F	5′-gctaactccaggacccatat-3′
CG13516_R	5′-gcgacaatagtggtttccag-3′
GAPDH_F	5′-gacgaaatcaaggctaaggtcg-3′
GAPDH_R	5′-aatgggtgtcgctgaagaagtc-3′
RP49_F	5′-ccgcttcaagggacagtatc-3′
RP49_R	5′-gacaatctccttgcgcttct-3′
