# Supplementary material for: Single amino-acid mutation in a Drosoph ila melanogaster ribosomal protein: An insight in uL11 transcriptional activity
Source: PLoS One. 2022 Aug 18;17(8):e0273198. doi: 10.1371/journal.pone.0273198 (PMC9387862; doi:10.1371/journal.pone.0273198)
Supplement: S2 Table — (PDF) [file pone.0273198.s007.pdf]

Sample name	Genotype	Total reads	Unmapped reads	Multimapped reads	Uniquely mapped reads used for the analysis
wc_1	*w^{1118} ; $corto^{+}$*	1.50E+07	8.56E+05	4.29E+05	1.36E+07
wc_2	*w^{1118} ; $corto^{+}$*	1.50E+07	1.17E+06	4.33E+05	1.33E+07
cortoL1420_1	*w^{1118} ; $corto^{L1}$/$corto^{420}$*	2.71E+07	1.24E+06	1.85E+06	2.37E+07
cortoL1420_2	*w^{1118} ; $corto^{L1}$/$corto^{420}$*	2.71E+07	1.83E+06	1.86E+06	2.31E+07
w_1	*w^{1118} ; $uL11^{+}$*	2.58E+07	1.36E+05	3.04E+06	2.21E+07
w_2	*w^{1118} ; $uL11^{+}$*	3.22E+07	1.44E+05	2.13E+06	2.97E+07
w_3	*w^{1118} ; $uL11^{+}$*	3.49E+07	3.90E+05	3.37E+06	3.06E+07
K3A_1	*w^{1118} ; $uL11^{K3A}$*	2.53E+07	2.11E+05	2.07E+06	2.15E+07
K3A_2	*w^{1118} ; $uL11^{K3A}$*	4.01E+07	1.66E+06	2.32E+07	1.16E+07
K3A_3	*w^{1118} ; $uL11^{K3A}$*	3.28E+07	6.25E+05	1.05E+07	1.91E+07
K3Y_1	*w^{1118} ; $uL11^{K3Y}$*	3.07E+07	2.06E+05	2.11E+06	2.81E+07
K3Y_2	*w^{1118} ; $uL11^{K3Y}$*	3.16E+07	9.03E+05	2.86E+06	2.74E+07
K3Y_3	*w^{1118} ; $uL11^{K3Y}$*	3.34E+07	3.10E+05	1.67E+06	3.12E+07
