# Supplementary material for: Single amino-acid mutation in a Drosoph ila melanogaster ribosomal protein: An insight in uL11 transcriptional activity
Source: PLoS One. 2022 Aug 18;17(8):e0273198. doi: 10.1371/journal.pone.0273198 (PMC9387862; doi:10.1371/journal.pone.0273198)
Supplement: S3 Table — Green: Up-regulated genes, log2 fold-change > 0.5, adjusted p-value < 5.E-02. Orange: Down-regulated genes, log2 fold-change < -0.5, adjusted p-value < 5.E-02. Blue: Genes up-regulated in other RPG mutants [35, 36]. (PDF) [file pone.0273198.s008.pdf]

Gene ID	Gene symbol	Base Mean	log_2FoldChange *corto*^{L1}/*corto*^{420} versus *w*^{1118}	adj p-value *corto*^{L1}/*corto*^{420} versus *w*^{1118}	log_2FoldChange *uL11*^{K3A} versus *w*^{1118}	adj p-value *uL11*^{K3A} versus *w*^{1118}	log_2FoldChange *uL11*^{K3Y} versus *w*^{1118}	adj p-value *uL11*^{K3Y} versus *w*^{1118}
FBgn0085813	18SrRNA-Psi:CR41602	492	0.12	5.47E-01	2.53	1.23E-03	-0.01	1.00E+00
FBgn0085753	28SrRNA-Psi:CR40596	815	0.17	2.96E-01	1.41	1.30E-02	-0.01	1.00E+00
FBgn0085771	28SrRNA-Psi:CR40741	96	3.34	8.74E-05	3.25	5.48E-05	-0.01	1.00E+00
FBgn0267508	28SrRNA-Psi:CR45848	67	0.18	3.14E-01	2.82	4.86E-04	0.00	1.00E+00
FBgn0267511	28SrRNA-Psi:CR45851	67	-0.02	8.65E-01	1.85	1.04E-02	0.00	1.00E+00
FBgn0267513	28SrRNA-Psi:CR45853	36	0.04	8.53E-01	2.51	1.39E-03	-0.01	1.00E+00
FBgn0267515	28SrRNA-Psi:CR45855	55	0.08	5.85E-01	1.60	9.23E-03	0.00	1.00E+00
FBgn0267520	28SrRNA-Psi:CR45860	204	0.27	8.96E-02	1.47	1.26E-02	0.00	1.00E+00
FBgn0052016	4E-T	2991	-0.97	6.64E-13	-0.07	3.82E-01	0.00	1.00E+00
FBgn0023129	aay	91	-0.12	6.58E-01	-1.00	6.49E-03	-0.18	1.45E-01
FBgn0039890	ABCD	2965	-0.64	4.77E-10	-0.08	2.74E-01	-0.06	2.18E-01
FBgn0004852	Ac76E	330	-3.26	6.23E-33	0.04	6.42E-01	0.00	1.00E+00
FBgn0027620	Acf	2690	-0.62	3.33E-06	0.02	8.64E-01	0.01	9.06E-01
FBgn0051865	Ada1-1	19	0.05	8.42E-01	-5.48	5.96E-05	0.07	3.78E-01
FBgn0026086	Adar	2162	-0.71	2.33E-05	-0.01	9.70E-01	0.00	1.00E+00
FBgn0026573	ADD1	1787	-0.61	3.07E-10	-0.02	9.06E-01	0.01	1.00E+00
FBgn0052068	Adi1	187	0.12	6.12E-01	0.41	5.04E-03	0.79	4.87E-07
FBgn0038223	Afti	793	-0.64	8.75E-06	0.00	1.00E+00	0.00	1.00E+00
FBgn0029813	AgmNAT	22	0.59	2.40E-02	0.05	6.08E-01	0.00	1.00E+00
FBgn0250816	AGO3	64	-0.64	1.52E-02	2.09	1.17E-13	0.38	5.07E-02
FBgn0024912	agt	851	0.61	3.04E-06	1.63	1.55E-43	0.21	4.55E-02
FBgn0014455	Ahcy	3702	0.65	5.05E-11	0.02	8.74E-01	0.00	1.00E+00
FBgn0033351	AIMP1	804	0.72	4.73E-05	0.05	5.43E-01	0.00	1.00E+00
FBgn0050185	AIMP3	582	0.65	2.84E-04	0.03	7.70E-01	0.00	1.00E+00
FBgn0027932	Akap200	21721	-1.15	5.83E-49	0.04	6.85E-01	0.00	1.00E+00
FBgn0012036	Aldh	651	0.77	6.08E-03	0.00	9.72E-01	0.07	4.09E-01
FBgn0011297	Alg3	292	0.70	8.24E-06	-0.28	7.21E-03	-0.01	9.05E-01
FBgn0283479	Alp1	47	0.10	5.76E-01	-0.60	5.71E-02	-0.01	1.00E+00
FBgn0033423	Alp6	12	-0.02	8.84E-01	-1.02	3.05E-02	-0.09	3.70E-01
FBgn0034710	Alp7	85	-0.13	2.20E-01	-8.44	5.06E-06	-0.01	1.00E+00
FBgn0034712	Alp8	99	-0.07	4.75E-01	-0.66	5.26E-02	0.00	1.00E+00
FBgn0086361	alph	3610	-0.65	1.44E-17	0.04	6.45E-01	0.00	1.00E+00
FBgn0015571	alpha-Est3	211	0.63	2.46E-03	0.42	6.49E-03	0.00	1.00E+00
FBgn0003884	alphaTub84B	98958	0.58	2.30E-12	-0.03	7.61E-01	-0.03	4.82E-01
FBgn0003886	alphaTub85E	97	3.34	1.93E-15	0.01	8.95E-01	0.01	9.37E-01
FBgn0031068	Alr	351	0.61	1.35E-03	0.39	6.66E-03	0.02	8.04E-01
FBgn0000075	amd	647	0.26	1.88E-01	1.20	1.13E-18	0.00	1.00E+00
FBgn0025686	Amnionless	12	-0.02	9.37E-01	-0.61	5.12E-02	-0.01	1.00E+00
FBgn0030328	Amun	7039	-0.79	7.59E-17	0.01	9.64E-01	0.00	1.00E+00
FBgn0012037	Ance	10590	-0.08	5.00E-01	-0.70	3.42E-22	-0.03	4.37E-01
FBgn0032535	Ance-2	17	-0.11	5.24E-01	-0.59	4.28E-02	0.00	1.00E+00
FBgn0032536	Ance-3	9	0.02	8.74E-01	1.73	1.06E-02	0.01	1.00E+00
FBgn0011747	Ank	7198	-0.65	1.55E-07	-0.04	6.39E-01	0.00	1.00E+00
FBgn0052000	anne	6510	-0.58	4.18E-05	0.01	9.54E-01	0.01	1.00E+00
FBgn0260642	Antp	1348	-0.77	7.50E-06	-0.02	8.95E-01	0.01	1.00E+00
FBgn0267408	AOX1	329	0.55	2.96E-02	3.81	3.49E-23	0.01	1.00E+00
FBgn0036111	Aps	7280	-0.69	1.35E-08	0.02	8.52E-01	0.00	1.00E+00
FBgn0286516	aqz	7288	-1.14	3.19E-11	-0.03	8.04E-01	0.00	1.00E+00
FBgn0033928	Arc2	184	0.03	9.37E-01	3.19	8.75E-82	0.22	8.41E-02
FBgn0038893	Archease	334	0.25	2.21E-01	0.63	7.35E-06	0.00	1.00E+00
FBgn0013749	Arf102F	1709	-0.65	3.80E-07	-0.06	5.12E-01	-0.01	1.00E+00
FBgn0017418	ari-1	1097	-0.60	1.00E-06	0.04	6.78E-01	0.00	1.00E+00
FBgn0041164	armi	2051	-0.65	2.47E-07	-0.05	6.22E-01	0.00	1.00E+00
FBgn0031050	Arp10	640	0.68	3.14E-04	0.00	9.77E-01	0.00	1.00E+00
FBgn0001961	Arpc1	2195	0.59	5.56E-08	0.02	8.26E-01	-0.01	1.00E+00
FBgn0284255	Arpc4	1043	1.13	5.45E-17	0.08	3.28E-01	0.00	1.00E+00
FBgn0031437	Arpc5	1308	0.77	7.24E-08	0.00	9.90E-01	-0.01	9.73E-01
FBgn0033062	Ars2	4686	-0.64	7.16E-18	-0.01	9.72E-01	0.00	1.00E+00
FBgn0039908	Asator	1415	-0.76	7.99E-10	-0.04	7.13E-01	0.00	1.00E+00
FBgn0270926	AsnS	394	1.20	2.78E-06	0.16	1.49E-01	0.17	1.29E-01
FBgn0034793	asrij	688	0.86	1.78E-07	0.36	3.09E-03	0.00	1.00E+00
FBgn0017424	asRNA:CR11538	422	0.58	1.49E-02	-0.04	7.33E-01	0.01	1.00E+00
FBgn0265295	asRNA:CR42871	56	-0.73	4.32E-03	0.11	3.22E-01	0.01	9.91E-01
FBgn0263344	asRNA:CR43425	78	-0.50	3.31E-02	0.80	9.37E-03	0.00	1.00E+00
FBgn0263345	asRNA:CR43426	51	0.67	1.10E-02	0.01	9.32E-01	-0.01	1.00E+00
FBgn0263445	asRNA:CR43468	99	-0.59	7.51E-03	0.27	4.57E-02	0.02	7.54E-01
FBgn0264822	asRNA:CR44030	121	-0.10	7.14E-01	0.86	2.95E-04	1.64	7.16E-13
FBgn0264823	asRNA:CR44031	97	0.54	1.87E-02	1.28	2.34E-03	0.02	7.92E-01

FBgn0264837	asRNA:CR44045	82	-0.61	1.50E-02	-0.01	9.70E-01	0.01	1.00E+00
FBgn0265499	asRNA:CR44368	337	0.00	9.87E-01	1.03	2.46E-15	-0.60	2.20E-04
FBgn0265613	asRNA:CR44431	123	0.83	5.37E-04	-0.03	7.85E-01	0.01	1.00E+00
FBgn0266619	asRNA:CR45126	267	-0.96	7.58E-07	2.40	2.95E-15	0.95	3.69E-03
FBgn0266633	asRNA:CR45140	31	0.34	1.70E-01	4.88	2.30E-13	-0.02	8.78E-01
FBgn0266681	asRNA:CR45171	27	-1.00	3.76E-03	0.34	9.99E-02	0.05	5.27E-01
FBgn0266692	asRNA:CR45182	29	-0.64	1.70E-02	-0.03	7.96E-01	0.01	1.00E+00
FBgn0266705	asRNA:CR45195	68	0.10	7.19E-01	7.75	1.00E-27	0.00	1.00E+00
FBgn0267022	asRNA:CR45466	32	-0.97	1.72E-03	0.00	9.72E-01	0.00	1.00E+00
FBgn0267160	asRNA:CR45600	111	-5.01	2.08E-12	5.59	1.04E-17	5.92	1.38E-19
FBgn0267223	asRNA:CR45663	34	1.49	3.22E-04	0.02	9.09E-01	0.00	1.00E+00
FBgn0267302	asRNA:CR45738	32	0.61	2.24E-02	-0.08	4.91E-01	-0.01	1.00E+00
FBgn0267439	asRNA:CR45789	35	-0.71	1.03E-02	-0.03	7.98E-01	0.00	1.00E+00
FBgn0267485	asRNA:CR45835	898	0.61	4.33E-07	-0.03	7.65E-01	0.00	1.00E+00
FBgn0267550	asRNA:CR45890	135	0.74	2.39E-03	-0.09	4.80E-01	0.00	1.00E+00
FBgn0267758	asRNA:CR46089	20	0.02	9.50E-01	6.93	9.49E-07	0.01	1.00E+00
FBgn0015591	AstA	15	-0.47	4.28E-02	-0.85	2.71E-02	-0.03	7.69E-01
FBgn0033010	Atf6	1360	-0.98	3.85E-07	0.00	9.95E-01	0.00	1.00E+00
FBgn0037363	Atg17	1079	-0.92	3.17E-07	0.03	7.45E-01	0.00	1.00E+00
FBgn0032422	atilla	38	0.68	1.38E-02	-0.01	9.56E-01	0.00	1.00E+00
FBgn0016120	ATPsynD	4001	0.89	1.85E-13	-0.03	7.42E-01	0.00	1.00E+00
FBgn0028342	ATPsyndelta	2959	1.17	4.25E-42	0.05	5.61E-01	0.00	1.00E+00
FBgn0038224	ATPsynE	2430	0.91	4.10E-08	-0.01	9.18E-01	0.00	1.00E+00
FBgn0035032	ATPsynF	2536	0.99	9.61E-19	0.00	9.76E-01	0.00	1.00E+00
FBgn0020235	ATPsyngamma	5712	0.73	9.57E-18	0.01	9.13E-01	-0.01	1.00E+00
FBgn0016691	ATPsynO	3667	0.75	2.40E-11	0.01	9.10E-01	0.00	1.00E+00
FBgn0000150	awd	22227	0.68	1.45E-06	0.00	9.85E-01	0.00	1.00E+00
FBgn0013751	Awh	61	-0.10	7.33E-01	-1.11	2.95E-03	0.00	1.00E+00
FBgn0004870	bab1	47	0.12	6.72E-01	-1.35	7.56E-05	0.00	1.00E+00
FBgn0011300	babo	2914	-0.80	1.14E-09	-0.04	7.06E-01	0.00	1.00E+00
FBgn0031453	Bacc	14814	-1.10	3.05E-25	0.05	5.22E-01	0.01	9.02E-01
FBgn0031255	BBS8	105	0.44	6.81E-02	0.88	5.53E-05	0.01	1.00E+00
FBgn0052594	be	704	-0.59	7.31E-05	-0.07	4.14E-01	0.00	1.00E+00
FBgn0000173	ben	4467	-0.60	5.15E-06	-0.04	7.26E-01	0.00	1.00E+00
FBgn0260860	Bet5	305	0.66	1.09E-03	0.02	8.60E-01	0.00	1.00E+00
FBgn0284243	betaTub56D	87388	0.65	3.71E-15	-0.02	8.83E-01	-0.01	1.00E+00
FBgn0035871	BI-1	1623	-0.69	7.33E-12	0.30	5.15E-05	0.00	1.00E+00
FBgn0000183	BicD	2135	-0.68	7.68E-09	0.06	4.83E-01	0.00	1.00E+00
FBgn0024491	Bin1	1697	0.32	1.04E-01	2.12	1.19E-36	0.00	1.00E+00
FBgn0026262	bip2	3652	-1.02	1.89E-14	-0.07	3.85E-01	0.01	8.48E-01
FBgn0085284	Blos3	282	0.75	3.15E-04	0.04	6.56E-01	0.00	1.00E+00
FBgn0036449	bmm	105	0.20	4.43E-01	-1.35	1.15E-03	-0.01	1.00E+00
FBgn0037007	BNIP3	2102	-0.67	5.46E-06	0.02	8.57E-01	0.00	1.00E+00
FBgn0014135	bnl	117	-0.63	1.26E-02	0.02	8.64E-01	0.00	1.00E+00
FBgn0261284	bou	1262	0.81	1.17E-08	0.09	2.46E-01	0.00	1.00E+00
FBgn0050169	Brca2	341	-0.64	8.41E-05	0.00	9.81E-01	0.00	1.00E+00
FBgn0000216	Brd	509	0.53	2.95E-03	1.17	7.95E-18	0.01	1.00E+00
FBgn0264001	bru3	51	0.05	8.22E-01	4.90	3.37E-15	-0.01	1.00E+00
FBgn0261822	Bsg	2702	-0.77	1.00E-06	0.00	9.76E-01	0.00	1.00E+00
FBgn0000229	bsk	1275	-0.72	5.53E-12	0.03	7.89E-01	0.00	1.00E+00
FBgn0000233	btd	63	0.21	3.76E-01	-2.19	6.57E-06	0.01	1.00E+00
FBgn0023096	btv	49	-0.11	6.87E-01	0.58	6.50E-02	0.03	7.38E-01
FBgn0259176	bun	5539	-0.70	3.32E-07	-0.41	7.00E-04	0.01	8.66E-01
FBgn0000246	c(3)G	214	-0.86	2.99E-05	0.06	5.01E-01	0.01	1.00E+00
FBgn0004863	C15	235	0.36	4.16E-02	-0.61	5.84E-06	0.00	1.00E+00
FBgn0263111	cac	124	0.37	1.37E-01	3.85	3.50E-29	-0.02	9.52E-01
FBgn0053653	Cadps	187	0.69	9.19E-03	1.58	2.50E-09	0.01	1.00E+00
FBgn0030054	Caf1-180	1658	-0.58	2.62E-04	0.04	6.21E-01	0.01	1.00E+00
FBgn0039928	Cals	4732	-0.77	1.60E-05	-0.03	8.06E-01	0.00	1.00E+00
FBgn0000253	Cam	12315	-1.48	3.62E-35	0.03	7.66E-01	0.00	1.00E+00
FBgn0016126	CaMKI	3031	-0.81	2.36E-10	0.00	9.77E-01	0.01	1.00E+00
FBgn0264607	CaMKII	2738	-0.98	1.88E-12	0.13	1.18E-01	0.08	2.06E-01
FBgn0267912	CanA-14F	856	-0.61	1.02E-04	0.04	6.99E-01	0.00	1.00E+00
FBgn0037831	Cap-H2	1038	-0.59	1.70E-07	0.03	7.58E-01	0.07	2.13E-01
FBgn0004878	cas	19	0.61	1.87E-02	0.38	8.83E-02	0.00	1.00E+00
FBgn0285954	caz	3727	-0.58	1.17E-03	0.06	5.27E-01	0.00	1.00E+00
FBgn0031148	Cbs	2322	0.62	2.00E-17	0.05	5.13E-01	0.00	1.00E+00
FBgn0030954	CCKLR-17D3	380	0.67	3.15E-04	0.06	4.84E-01	0.00	1.00E+00
FBgn0010621	CCT5	11598	0.65	5.89E-18	0.05	5.39E-01	-0.04	3.73E-01
FBgn0052499	Cda4	4755	0.63	7.21E-13	0.03	7.32E-01	0.02	6.49E-01
FBgn0027491	Cdk5alpha	35	-0.75	9.09E-03	-0.09	3.69E-01	0.00	1.00E+00
FBgn0034437	CG10051	15	-0.03	8.98E-01	-2.23	8.63E-03	-0.38	9.09E-02
FBgn0036369	CG10089	215	-0.17	4.09E-01	0.84	4.59E-09	-0.35	2.02E-02
FBgn0032800	CG10137	20	0.26	2.83E-01	0.65	3.90E-02	0.00	1.00E+00
FBgn0032793	CG10189	280	0.09	6.85E-01	0.61	7.79E-06	0.00	1.00E+00
FBgn0038454	CG10324	235	-0.83	9.43E-06	0.06	4.80E-01	0.00	1.00E+00
FBgn0032805	CG10337	141	-0.44	3.08E-02	0.94	4.60E-08	0.00	1.00E+00
FBgn0034972	CG10339	12	0.07	6.39E-01	2.10	6.08E-04	1.55	5.81E-03
FBgn0034729	CG10344	43	-0.05	8.66E-01	1.22	1.10E-03	0.05	4.80E-01

FBgn0033019	CG10395	486	-0.99	3.91E-12	0.15	5.06E-02	0.00	1.00E+00
FBgn0033021	CG10417	4149	-0.87	9.78E-43	-0.05	4.73E-01	-0.02	6.07E-01
FBgn0036277	CG10418	472	0.97	6.82E-07	0.03	8.02E-01	0.00	1.00E+00
FBgn0037531	CG10445	247	-0.36	7.85E-02	0.81	6.20E-08	0.02	6.24E-01
FBgn0033017	CG10465	1501	-0.72	2.95E-08	-0.06	4.92E-01	-0.01	1.00E+00
FBgn0039312	CG10514	39	-1.49	7.99E-04	0.02	8.51E-01	0.00	1.00E+00
FBgn0039323	CG10559	50	0.14	5.95E-01	1.51	1.14E-08	0.00	1.00E+00
FBgn0032717	CG10600	1849	-0.73	1.91E-05	0.01	9.49E-01	0.00	1.00E+00
FBgn0036290	CG10638	2684	0.14	2.79E-01	1.24	2.06E-62	0.00	1.00E+00
FBgn0029666	CG10803	1895	-0.74	7.57E-06	-0.03	7.80E-01	0.00	1.00E+00
FBgn0027552	CG10863	209	0.62	2.02E-02	-0.03	7.37E-01	0.01	1.00E+00
FBgn0034312	CG10916	405	-0.21	2.73E-01	1.32	2.49E-27	0.00	1.00E+00
FBgn0033149	CG11060	14	0.19	1.86E-01	0.63	4.56E-02	0.00	1.00E+00
FBgn0039930	CG11077	321	0.68	3.96E-04	0.04	6.86E-01	0.00	1.00E+00
FBgn0030519	CG11151	1004	0.66	1.09E-04	-0.04	6.70E-01	0.00	1.00E+00
FBgn0039927	CG11155	89	-0.20	4.49E-01	0.59	1.37E-02	-0.01	1.00E+00
FBgn0030511	CG11158	89	-0.09	7.52E-01	1.15	9.94E-05	0.00	1.00E+00
FBgn0034528	CG11180	768	-1.31	7.99E-12	0.06	4.93E-01	0.00	1.00E+00
FBgn0037115	CG11249	31	-0.27	2.75E-01	0.66	1.81E-02	0.00	1.00E+00
FBgn0036334	CG11267	2756	0.80	2.32E-09	-0.01	9.69E-01	0.00	1.00E+00
FBgn0035552	CG11350	22	1.08	2.47E-03	0.01	9.10E-01	0.07	4.10E-01
FBgn0039920	CG11360	3374	-0.59	2.82E-03	0.08	3.43E-01	0.01	1.00E+00
FBgn0037181	CG11370	1620	0.27	2.29E-01	0.76	6.42E-04	0.14	1.74E-01
FBgn0031224	CG11454	503	0.73	1.74E-06	0.04	6.54E-01	0.00	1.00E+00
FBgn0037396	CG11459	40	0.02	9.37E-01	-3.59	5.30E-04	-0.36	9.07E-02
FBgn0031244	CG11601	288	-1.03	8.59E-12	0.02	8.70E-01	0.00	1.00E+00
FBgn0036196	CG11658	72	0.19	4.82E-01	0.80	4.86E-04	0.00	1.00E+00
FBgn0030311	CG11699	609	0.68	5.97E-04	0.03	7.59E-01	0.00	1.00E+00
FBgn0030292	CG11752	421	0.85	9.10E-06	-0.02	8.49E-01	0.00	1.00E+00
FBgn0037603	CG11753	305	0.71	1.85E-04	0.00	9.72E-01	0.00	1.00E+00
FBgn0031264	CG11835	652	-0.12	5.99E-01	-0.96	6.46E-07	-0.07	2.96E-01
FBgn0039299	CG11854	130	-0.09	5.51E-01	-0.89	3.53E-02	-0.01	1.00E+00
FBgn0014427	CG11899	2687	0.58	2.36E-04	-0.04	6.96E-01	-0.04	4.40E-01
FBgn0037312	CG11999	699	0.71	3.22E-04	0.09	3.31E-01	0.00	1.00E+00
FBgn0039831	CG12054	2044	-0.78	9.35E-06	-0.01	9.62E-01	0.00	1.00E+00
FBgn0030098	CG12057	41	0.04	6.75E-01	-3.13	4.56E-03	-1.77	1.09E-02
FBgn0037386	CG1208	54	3.54	4.06E-08	1.32	3.43E-03	0.00	1.00E+00
FBgn0030048	CG12112	271	-0.85	7.80E-06	0.12	1.48E-01	0.00	1.00E+00
FBgn0030097	CG12115	11	-0.08	5.01E-01	-5.99	2.31E-05	-3.05	1.15E-03
FBgn0037356	CG12170	204	0.70	6.12E-04	0.03	8.04E-01	0.01	1.00E+00
FBgn0037354	CG12171	763	1.03	5.36E-13	0.75	1.51E-13	0.00	1.00E+00
FBgn0030510	CG12177	62	0.10	7.32E-01	0.76	2.59E-03	0.00	1.00E+00
FBgn0031022	CG12204	290	1.03	3.91E-07	0.43	5.96E-03	0.01	9.42E-01
FBgn0037974	CG12224	199	-0.04	9.02E-01	2.01	3.75E-30	0.00	1.00E+00
FBgn0036514	CG12301	779	-0.59	1.10E-03	0.09	2.87E-01	0.00	1.00E+00
FBgn0038590	CG12320	383	0.61	4.18E-04	0.06	4.68E-01	0.00	1.00E+00
FBgn0033558	CG12344	18	-0.73	1.06E-02	-0.07	5.45E-01	0.00	1.00E+00
FBgn0029657	CG12535	259	-2.49	3.99E-31	0.04	6.98E-01	0.00	1.00E+00
FBgn0250830	CG12547	2267	-0.82	2.09E-21	0.05	5.86E-01	0.00	1.00E+00
FBgn0037811	CG12592	84	-5.00	3.37E-05	-0.04	6.97E-01	-0.01	1.00E+00
FBgn0037796	CG12814	1934	-0.66	1.10E-11	0.05	5.48E-01	0.03	5.19E-01
FBgn0033145	CG12828	151	0.87	1.14E-04	-0.05	6.52E-01	-0.02	8.92E-01
FBgn0033945	CG12868	154	-0.36	1.17E-01	1.52	3.95E-16	0.00	1.00E+00
FBgn0033547	CG12935	370	1.10	1.08E-10	-0.02	8.88E-01	0.00	1.00E+00
FBgn0037753	CG12947	202	-0.18	4.28E-01	-0.63	3.27E-04	0.00	1.00E+00
FBgn0035501	CG1299	414	0.26	2.45E-01	1.01	2.72E-08	0.00	1.00E+00
FBgn0030859	CG12990	39	-1.81	3.29E-05	0.02	8.45E-01	0.00	1.00E+00
FBgn0036677	CG13023	385	2.05	7.22E-11	-0.32	5.69E-02	-0.14	1.79E-01
FBgn0036670	CG13029	41	0.35	1.31E-01	2.92	1.84E-18	0.12	2.40E-01
FBgn0036605	CG13041	54	1.02	3.83E-03	-0.07	4.61E-01	-0.40	3.96E-02
FBgn0036599	CG13044	661	0.68	4.80E-03	0.05	5.92E-01	0.02	8.05E-01
FBgn0036596	CG13045	48	-0.59	2.66E-02	-0.06	5.40E-01	-0.01	1.00E+00
FBgn0036660	CG13045	470	-0.83	4.04E-09	0.18	4.71E-02	0.13	1.00E-01
FBgn0040801	CG13053	118	0.97	1.90E-03	-0.96	9.37E-03	-1.91	1.24E-04
FBgn0040796	CG13064	52	0.81	5.07E-03	0.05	6.12E-01	0.00	1.00E+00
FBgn0036589	CG13067	78	0.87	4.69E-03	0.06	5.01E-01	1.27	1.22E-05
FBgn0032803	CG13082	663	0.62	4.19E-03	-0.07	4.59E-01	0.00	1.00E+00
FBgn0032051	CG13097	1071	-0.63	3.97E-06	0.08	3.18E-01	0.00	1.00E+00
FBgn0033608	CG13220	1046	0.59	1.39E-06	0.05	5.80E-01	0.00	1.00E+00
FBgn0033781	CG13319	429	0.85	1.59E-05	0.03	7.98E-01	0.00	1.00E+00
FBgn0029531	CG13362	69	-0.74	3.92E-03	0.03	7.39E-01	-0.04	6.33E-01
FBgn0026879	CG13364	1664	0.76	6.87E-06	0.00	9.93E-01	0.00	1.00E+00
FBgn0025640	CG13369	355	0.63	8.83E-04	-0.01	9.12E-01	0.00	1.00E+00
FBgn0040658	CG13516	84	0.91	8.75E-04	-2.22	4.86E-15	-3.17	1.48E-27
FBgn0035010	CG13579	278	-0.03	8.28E-01	11.51	4.85E-23	0.00	1.00E+00
FBgn0035020	CG13585	575	0.95	4.94E-11	0.13	1.02E-01	0.02	7.52E-01
FBgn0039219	CG13630	1091	0.64	1.67E-09	0.10	1.19E-01	0.00	1.00E+00
FBgn0035859	CG13678	134	1.06	1.15E-03	0.02	8.64E-01	0.09	3.13E-01
FBgn0030539	CG1368	104	0.83	4.40E-03	-1.49	1.89E-04	-0.01	1.00E+00

FBgn0031254	CG13692	54	0.04	8.97E-01	1.31	2.65E-05	0.01	1.00E+00
FBgn0035553	CG13722	58	0.93	4.69E-03	-0.23	1.74E-01	-0.13	2.10E-01
FBgn0036382	CG13737	57	0.62	1.26E-02	0.04	6.72E-01	0.00	1.00E+00
FBgn0033340	CG13751	510	1.29	9.93E-08	0.03	7.26E-01	0.00	1.00E+00
FBgn0031834	CG13766	269	-1.05	7.32E-11	-0.06	5.06E-01	0.00	1.00E+00
FBgn0035176	CG13905	32	1.11	1.36E-03	0.01	9.54E-01	0.00	1.00E+00
FBgn0040786	CG14104	193	1.28	2.01E-07	-0.08	3.84E-01	-0.01	1.00E+00
FBgn0040817	CG14132	340	0.11	6.73E-01	-0.62	1.97E-03	-0.03	5.64E-01
FBgn0038658	CG14292	69	-1.15	1.15E-03	0.06	6.08E-01	0.00	1.00E+00
FBgn0038647	CG14302	16	-0.04	7.60E-01	-1.37	2.08E-02	-0.01	1.00E+00
FBgn0038629	CG14304	297	0.08	7.08E-01	0.62	1.63E-05	0.00	1.00E+00
FBgn0038581	CG14314	75	-0.47	5.04E-02	1.19	4.26E-05	0.00	1.00E+00
FBgn0038148	CG14377	80	-1.02	2.52E-04	-0.09	4.73E-01	-0.01	1.00E+00
FBgn0032900	CG14401	26	-0.99	2.51E-03	-0.02	8.90E-01	-0.01	1.00E+00
FBgn0030584	CG14407	427	0.78	1.12E-04	0.00	9.86E-01	0.00	1.00E+00
FBgn0029894	CG14440	648	-0.66	2.53E-05	-0.01	9.13E-01	0.00	1.00E+00
FBgn0033000	CG14464	898	-0.68	2.86E-05	0.04	7.03E-01	0.00	1.00E+00
FBgn0037127	CG14566	326	1.00	4.07E-05	-0.02	8.40E-01	0.52	1.64E-02
FBgn0037503	CG14598	331	1.24	1.79E-12	-0.18	6.31E-02	0.00	1.00E+00
FBgn0031184	CG14615	61	-0.84	2.10E-03	-0.02	8.61E-01	-0.03	6.96E-01
FBgn0037850	CG14695	97	0.09	5.32E-01	7.33	1.55E-43	0.03	7.36E-01
FBgn0037930	CG14715	686	0.87	8.24E-08	-0.03	7.95E-01	-0.01	1.00E+00
FBgn0033243	CG14763	94	-0.69	4.53E-03	-0.06	6.21E-01	0.00	1.00E+00
FBgn0038455	CG14907	184	-0.29	2.10E-01	0.97	2.91E-09	-0.13	1.54E-01
FBgn0032335	CG14915	24	0.28	2.37E-01	0.64	2.32E-02	0.02	9.05E-01
FBgn0035409	CG14963	27	0.00	9.87E-01	0.01	9.11E-01	0.69	4.79E-02
FBgn0035414	CG14965	340	-0.68	9.14E-05	-0.02	8.87E-01	0.00	1.00E+00
FBgn0035415	CG14966	318	-0.70	7.73E-05	-0.03	7.86E-01	-0.01	9.03E-01
FBgn0035469	CG14977	355	0.66	3.70E-04	0.01	9.69E-01	-0.01	1.00E+00
FBgn0035480	CG14984	277	0.75	9.39E-04	0.23	5.87E-02	0.01	1.00E+00
FBgn0034399	CG15083	584	0.79	1.63E-04	0.02	8.17E-01	0.00	1.00E+00
FBgn0032733	CG15170	18	0.02	9.12E-01	-2.23	8.48E-03	-0.18	1.79E-01
FBgn0030234	CG15211	42	0.60	2.38E-02	0.00	9.86E-01	0.01	1.00E+00
FBgn0033104	CG15237	463	0.71	3.29E-05	0.03	8.00E-01	0.00	1.00E+00
FBgn0030183	CG15309	348	-1.15	2.11E-07	0.03	7.91E-01	0.00	1.00E+00
FBgn0030029	CG15343	65	-0.08	7.69E-01	-1.24	6.05E-03	-0.01	1.00E+00
FBgn0030040	CG15347	54	4.74	6.59E-09	0.00	9.83E-01	-0.03	7.54E-01
FBgn0040718	CG15353	3826	0.82	1.11E-05	0.03	7.62E-01	0.61	2.02E-03
FBgn0031393	CG15382	81	-0.62	8.94E-03	-0.11	2.88E-01	-0.01	1.00E+00
FBgn0039828	CG1542	1295	0.67	1.31E-06	0.06	5.20E-01	-0.01	1.00E+00
FBgn0029719	CG15473	26	0.74	8.74E-03	0.00	9.77E-01	0.00	1.00E+00
FBgn0039807	CG15546	179	0.73	3.79E-04	0.75	4.93E-04	0.03	6.76E-01
FBgn0031632	CG15628	830	-0.69	6.10E-05	-0.02	8.10E-01	0.02	6.86E-01
FBgn0030309	CG1572	778	0.65	7.98E-06	-0.10	1.88E-01	0.00	1.00E+00
FBgn0029766	CG15784	8033	0.41	1.73E-02	5.32	0.00E+00	0.61	6.92E-05
FBgn0030246	CG1582	1397	0.03	8.89E-01	0.74	2.03E-15	0.01	1.00E+00
FBgn0038814	CG15923	256	-1.32	5.53E-08	0.07	5.34E-01	0.01	1.00E+00
FBgn0033191	CG1598	1089	0.60	9.56E-06	0.05	5.99E-01	0.00	1.00E+00
FBgn0039602	CG1647	659	-0.61	1.64E-05	0.03	7.46E-01	0.01	1.00E+00
FBgn0038719	CG16727	101	-13.72	9.08E-07	0.00	9.77E-01	0.00	1.00E+00
FBgn0032488	CG16812	796	-0.63	2.20E-08	0.05	5.27E-01	0.00	1.00E+00
FBgn0032495	CG16820	141	-0.22	3.58E-01	1.11	4.35E-10	-0.63	3.41E-03
FBgn0030321	CG1703	2733	-0.59	3.11E-05	0.17	5.48E-02	0.00	1.00E+00
FBgn0040496	CG17104	334	-0.16	5.23E-01	1.06	2.36E-07	0.00	1.00E+00
FBgn0036440	CG17177	11	-0.03	8.46E-01	6.67	3.10E-06	0.00	1.00E+00
FBgn0038043	CG17202	1061	0.69	2.76E-04	0.01	9.18E-01	0.00	1.00E+00
FBgn0039977	CG17454	820	-0.64	7.83E-09	0.13	4.25E-02	0.00	1.00E+00
FBgn0032997	CG17486	1168	-0.59	1.85E-06	0.00	9.88E-01	0.00	1.00E+00
FBgn0039959	CG17514	7525	-0.60	2.64E-04	0.03	7.60E-01	0.00	1.00E+00
FBgn0261387	CG17528	2579	-0.62	4.04E-09	-0.01	9.46E-01	0.00	1.00E+00
FBgn0033777	CG17574	551	0.29	8.95E-02	0.91	1.94E-17	-0.67	1.94E-08
FBgn0031597	CG17612	367	-0.72	9.48E-06	0.04	7.15E-01	0.01	1.00E+00
FBgn0263780	CG17684	127	-0.31	1.98E-01	1.48	1.26E-18	-0.11	1.79E-01
FBgn0040056	CG17698	1191	-0.96	1.17E-14	-0.06	4.28E-01	0.00	1.00E+00
FBgn0033802	CG17724	203	-1.43	3.95E-06	0.06	4.74E-01	0.00	1.00E+00
FBgn0038718	CG17752	65	-2.13	4.83E-04	0.02	8.61E-01	0.00	1.00E+00
FBgn0040899	CG17776	374	1.06	1.51E-06	0.02	8.65E-01	0.00	1.00E+00
FBgn0040005	CG17883	1756	-0.62	7.07E-07	0.09	2.41E-01	0.00	1.00E+00
FBgn0028856	CG18063	238	-0.03	8.28E-01	8.80	7.19E-73	-0.03	7.66E-01
FBgn0038470	CG18213	611	-0.08	7.21E-01	0.82	1.62E-08	0.00	1.00E+00
FBgn0033431	CG1827	277	1.05	3.97E-06	-0.04	6.56E-01	0.00	1.00E+00
FBgn0030351	CG1840	156	-0.65	5.29E-03	-0.01	9.48E-01	0.00	1.00E+00
FBgn0037973	CG18547	919	0.26	1.35E-01	1.90	8.11E-70	-0.01	1.00E+00
FBgn0031929	CG18585	13	-0.02	8.82E-01	-1.09	2.80E-02	-0.09	3.38E-01
FBgn0033421	CG1888	455	0.63	3.47E-04	0.01	9.03E-01	0.00	1.00E+00
FBgn0039911	CG1909	92	0.51	4.75E-02	3.31	4.98E-37	0.04	5.75E-01
FBgn0022349	CG1910	5653	-0.77	1.49E-09	-0.01	9.26E-01	0.00	1.00E+00
FBgn0039881	CG1971	10	-0.03	9.12E-01	0.70	3.73E-02	0.00	1.00E+00
FBgn0039886	CG2003	66	0.01	9.87E-01	-0.65	4.84E-03	-0.09	2.74E-01

FBgn0039664	CG2006	513	0.76	3.00E-05	0.01	9.46E-01	0.00	1.00E+00
FBgn0035271	CG2021	304	0.65	5.50E-04	0.02	8.14E-01	0.00	1.00E+00
FBgn0033205	CG2064	103	-0.13	6.35E-01	1.83	2.85E-21	0.00	1.00E+00
FBgn0017448	CG2187	56	0.76	7.19E-03	1.48	2.14E-05	1.25	4.85E-04
FBgn0030447	CG2200	465	0.66	5.82E-05	-0.04	7.06E-01	-0.01	1.00E+00
FBgn0039665	CG2310	1991	0.82	6.19E-05	0.02	8.88E-01	0.00	1.00E+00
FBgn0034931	CG2812	789	0.77	1.36E-07	0.55	9.49E-07	0.52	2.63E-05
FBgn0023526	CG2865	935	-0.93	1.42E-06	0.02	8.50E-01	0.01	1.00E+00
FBgn0029679	CG2901	32	1.17	2.64E-03	0.06	5.87E-01	-0.01	1.00E+00
FBgn0030189	CG2909	129	0.00	9.91E-01	1.71	1.07E-19	0.01	1.00E+00
FBgn0023528	CG2924	2315	-0.73	1.93E-12	-0.05	5.33E-01	0.00	1.00E+00
FBgn0031643	CG3008	1831	-0.27	1.20E-02	1.75	8.62E-147	0.01	9.62E-01
FBgn0050187	CG30187	266	-0.12	6.49E-01	1.68	1.49E-24	0.00	1.00E+00
FBgn0050196	CG30196	60	0.19	2.15E-01	2.12	1.75E-11	0.08	3.26E-01
FBgn0050197	CG30197	45	0.25	3.20E-01	-2.87	5.43E-10	0.00	1.00E+00
FBgn0031645	CG3036	831	0.87	2.51E-06	0.65	6.15E-05	-0.01	8.88E-01
FBgn0050424	CG30424	15	-0.20	3.78E-01	-1.39	6.47E-03	-0.01	1.00E+00
FBgn0050428	CG30428	190	-0.09	7.38E-01	0.65	6.52E-04	0.86	1.95E-05
FBgn0050440	CG30440	2603	-0.80	1.00E-14	-0.07	3.70E-01	0.00	1.00E+00
FBgn0034816	CG3085	164	0.45	2.53E-02	0.73	1.11E-06	0.00	1.00E+00
FBgn0051030	CG31030	110	-0.64	1.41E-02	-0.16	1.63E-01	0.00	1.00E+00
FBgn0051055	CG31055	196	0.70	1.15E-03	-0.04	7.60E-01	0.00	1.00E+00
FBgn0033005	CG3107	2743	-0.71	1.56E-07	-0.01	9.10E-01	0.00	1.00E+00
FBgn0051076	CG31076	39	0.06	8.28E-01	-0.05	5.92E-01	-2.80	1.70E-05
FBgn0051126	CG31126	212	0.76	3.03E-04	0.02	8.97E-01	0.00	1.00E+00
FBgn0047114	CG31142	452	0.13	5.07E-01	0.59	2.76E-05	-0.01	9.42E-01
FBgn0051156	CG31156	386	-0.80	1.01E-06	0.03	7.46E-01	0.00	1.00E+00
FBgn0051157	CG31157	19	4.04	1.52E-04	0.08	5.01E-01	0.01	1.00E+00
FBgn0051391	CG31391	23	-0.69	1.21E-02	0.00	9.88E-01	0.00	1.00E+00
FBgn0051431	CG31431	21	-0.13	5.83E-01	-0.81	2.70E-02	0.00	1.00E+00
FBgn0051467	CG31467	117	-0.64	7.42E-03	0.00	9.83E-01	0.00	1.00E+00
FBgn0051510	CG31510	942	-1.10	4.98E-15	-0.09	2.64E-01	0.00	1.00E+00
FBgn0051548	CG31548	553	0.68	1.35E-05	0.02	8.45E-01	0.00	1.00E+00
FBgn0051549	CG31549	582	0.18	2.97E-01	0.93	1.89E-23	0.00	1.00E+00
FBgn0051559	CG31559	89	0.07	8.26E-01	-0.59	2.16E-02	-0.02	8.81E-01
FBgn0051633	CG31633	109	0.66	6.96E-03	0.14	1.73E-01	0.01	1.00E+00
FBgn0029896	CG3168	768	0.19	4.55E-01	1.17	3.76E-06	0.00	1.00E+00
FBgn0051898	CG31898	120	-0.71	1.78E-03	0.04	7.04E-01	0.01	1.00E+00
FBgn0031360	CG31937	567	0.78	1.14E-07	0.01	9.51E-01	0.00	1.00E+00
FBgn0051955	CG31955	41	0.02	9.57E-01	-0.16	2.03E-01	-1.28	1.29E-03
FBgn0051997	CG31997	2776	0.64	9.55E-06	0.08	3.37E-01	-0.14	9.82E-02
FBgn0051998	CG31998	3283	-0.73	1.38E-04	0.00	9.83E-01	0.01	1.00E+00
FBgn0051999	CG31999	642	0.48	4.40E-04	0.94	7.82E-23	-0.52	3.45E-06
FBgn0052017	CG32017	50	-0.13	6.30E-01	1.88	8.72E-06	0.03	6.95E-01
FBgn0052055	CG32055	76	0.88	1.45E-03	-1.68	1.88E-07	-2.31	1.15E-12
FBgn0052069	CG32069	283	0.66	8.71E-04	0.00	9.89E-01	-0.01	1.00E+00
FBgn0052095	CG32095	635	-0.64	3.32E-07	0.01	9.67E-01	0.00	1.00E+00
FBgn0052163	CG32163	213	0.66	1.33E-03	0.01	9.06E-01	0.00	1.00E+00
FBgn0052176	CG32176	526	-0.83	1.30E-07	0.02	8.39E-01	0.00	1.00E+00
FBgn0052243	CG32243	1236	-0.76	1.70E-15	0.01	9.47E-01	-0.01	1.00E+00
FBgn0052354	CG32354	120	0.18	4.83E-01	0.68	4.05E-03	0.73	5.80E-03
FBgn0041706	CG3253	101	0.07	8.05E-01	1.71	4.60E-19	-0.01	9.62E-01
FBgn0052532	CG32532	10	-0.17	4.17E-01	5.19	1.02E-04	4.29	1.22E-03
FBgn0052544	CG32544	30	-0.69	1.34E-02	0.00	9.85E-01	-0.01	1.00E+00
FBgn0052549	CG32549	668	0.19	4.29E-01	1.12	1.39E-07	-0.02	8.49E-01
FBgn0032986	CG3262	1798	-0.61	1.50E-16	0.23	1.11E-05	0.02	5.82E-01
FBgn0052625	CG32625	10	-0.04	8.29E-01	2.22	9.92E-04	0.00	1.00E+00
FBgn0052694	CG32694	95	0.22	3.56E-01	-0.84	2.94E-04	0.00	1.00E+00
FBgn0052850	CG32850	772	-1.46	4.07E-37	0.05	5.76E-01	0.00	1.00E+00
FBgn0053225	CG33225	21	0.60	1.61E-02	0.15	2.63E-01	0.00	1.00E+00
FBgn0053281	CG33281	14	-10.96	7.84E-06	0.05	6.42E-01	0.01	1.00E+00
FBgn0053509	CG33509	743	-0.64	6.34E-04	1.82	4.17E-46	0.19	6.27E-02
FBgn0031619	CG3355	63	0.66	1.35E-02	-0.04	6.88E-01	0.00	1.00E+00
FBgn0052831	CG33695	761	0.62	7.88E-05	-0.02	8.24E-01	0.00	1.00E+00
FBgn0262731	CG33941	610	-1.36	5.20E-10	0.05	5.38E-01	0.00	1.00E+00
FBgn0053978	CG33978	6959	-0.69	2.06E-04	0.11	2.26E-01	0.04	4.82E-01
FBgn0085192	CG34163	279	-0.70	1.49E-03	0.00	9.76E-01	0.00	1.00E+00
FBgn0033100	CG3420	534	0.60	6.60E-04	0.03	8.04E-01	0.00	1.00E+00
FBgn0085236	CG34207	212	-0.84	3.04E-05	-0.02	8.28E-01	0.00	1.00E+00
FBgn0085276	CG34247	87	4.23	8.81E-18	0.05	5.80E-01	-1.88	7.16E-04
FBgn0085411	CG34382	59	0.85	1.89E-03	-1.48	1.98E-05	-1.22	2.36E-04
FBgn0085452	CG34423	18	0.32	1.61E-01	-0.96	1.15E-02	-1.98	2.34E-04
FBgn0035996	CG3448	164	-0.30	1.51E-01	1.45	4.49E-18	-0.01	1.00E+00
FBgn0024984	CG3457	47	1.34	1.07E-04	1.75	3.05E-08	0.00	1.00E+00
FBgn0035007	CG3492	22	-0.03	8.41E-01	7.08	6.14E-09	-0.01	1.00E+00
FBgn0035008	CG3494	13	0.00	1.00E+00	7.85	3.38E-08	0.00	1.00E+00
FBgn0034849	CG3500	293	0.62	1.41E-03	0.01	9.05E-01	0.00	1.00E+00
FBgn0035063	CG3594	504	0.59	1.16E-03	0.06	4.53E-01	0.00	1.00E+00
FBgn0029824	CG3726	143	0.27	2.31E-01	0.78	5.21E-05	0.03	5.79E-01

FBgn0035085	CG3770	365	0.18	3.02E-01	-0.79	4.13E-11	-0.03	5.47E-01
FBgn0036422	CG3868	17	-0.06	6.16E-01	-1.60	1.44E-02	-0.28	1.22E-01
FBgn0058160	CG40160	3127	-0.80	2.78E-16	-0.05	5.08E-01	-0.01	9.40E-01
FBgn0058191	CG40191	1684	-0.60	5.47E-08	-0.06	4.68E-01	0.00	1.00E+00
FBgn0035986	CG4022	1651	-0.83	7.91E-10	-0.06	4.64E-01	-0.01	9.41E-01
FBgn0063670	CG40228	993	-1.01	2.80E-10	0.02	8.24E-01	0.00	1.00E+00
FBgn0039955	CG41099	2624	-0.75	2.20E-08	-0.07	4.01E-01	0.00	1.00E+00
FBgn0087011	CG41520	382	-0.08	7.78E-01	0.72	3.67E-04	-0.02	7.95E-01
FBgn0038302	CG4210	304	0.06	8.08E-01	0.84	6.58E-11	0.01	9.40E-01
FBgn0250862	CG42237	12	-0.60	2.25E-02	-0.05	6.20E-01	0.00	1.00E+00
FBgn0259143	CG42258	765	-1.18	1.27E-20	0.02	8.81E-01	0.00	1.00E+00
FBgn0266569	CG42259	221	-0.77	2.05E-03	0.02	8.22E-01	0.00	1.00E+00
FBgn0259222	CG42322	26	-0.66	1.61E-02	0.03	8.02E-01	0.01	1.00E+00
FBgn0259742	CG42360	459	-0.79	9.71E-08	0.07	4.15E-01	0.00	1.00E+00
FBgn0259711	CG42365	141	-0.21	3.83E-01	1.18	2.56E-11	0.00	1.00E+00
FBgn0034761	CG4250	49	0.70	1.17E-02	0.09	3.74E-01	0.01	1.00E+00
FBgn0260768	CG42566	16	0.14	5.66E-01	-1.69	2.81E-03	-0.84	1.90E-02
FBgn0034741	CG4269	32	-1.33	1.99E-03	-0.02	8.60E-01	0.01	1.00E+00
FBgn0261584	CG42694	19	-0.16	5.02E-01	-0.86	2.34E-02	0.00	1.00E+00
FBgn0261641	CG42724	4450	-0.87	2.78E-09	0.02	8.50E-01	0.00	1.00E+00
FBgn0261995	CG42813	479	0.60	7.35E-04	0.01	9.46E-01	0.00	1.00E+00
FBgn0034742	CG4294	1630	-0.72	3.08E-08	0.18	3.61E-02	0.02	7.92E-01
FBgn0038784	CG4362	57	0.04	8.38E-01	-1.24	1.81E-02	-0.59	5.15E-02
FBgn0263993	CG43736	3291	-0.60	2.45E-04	-0.03	7.64E-01	0.00	1.00E+00
FBgn0032132	CG4382	1610	1.20	1.55E-11	-0.43	3.97E-03	-0.64	3.44E-04
FBgn0038771	CG4390	2352	0.60	4.28E-06	-0.01	9.12E-01	-0.01	1.00E+00
FBgn0264748	CG44006	16	-0.19	3.72E-01	0.85	1.86E-02	0.00	1.00E+00
FBgn0264911	CG44102	35	0.02	9.40E-01	2.66	5.21E-15	0.00	1.00E+00
FBgn0265413	CG44325	1479	0.60	1.75E-04	-0.03	7.71E-01	0.00	1.00E+00
FBgn0266101	CG44838	1266	-0.58	3.40E-05	0.00	9.80E-01	0.00	1.00E+00
FBgn0031896	CG4502	1190	-0.65	9.37E-07	-0.07	3.45E-01	0.00	1.00E+00
FBgn0266410	CG45050	4461	-1.41	2.07E-17	0.01	9.46E-01	0.00	1.00E+00
FBgn0035006	CG4563	11	-0.02	8.74E-01	0.61	6.06E-02	0.00	1.00E+00
FBgn0037844	CG4570	35	1.56	1.77E-04	0.00	9.85E-01	0.01	1.00E+00
FBgn0035016	CG4612	3093	-1.01	2.21E-13	-0.03	7.29E-01	0.00	1.00E+00
FBgn0031299	CG4629	51	-0.05	8.82E-01	0.67	2.58E-02	0.00	1.00E+00
FBgn0030776	CG4653	10	-0.02	8.87E-01	-1.22	2.33E-02	-0.09	3.33E-01
FBgn0038739	CG4686	1110	0.67	8.46E-09	-0.08	2.57E-01	0.00	1.00E+00
FBgn0037992	CG4702	49	0.17	5.07E-01	-1.32	4.95E-04	-0.36	5.98E-02
FBgn0033820	CG4716	80	0.10	4.21E-01	-2.55	6.25E-03	0.02	9.19E-01
FBgn0030790	CG4768	1064	-0.63	3.80E-07	-0.05	5.59E-01	0.00	1.00E+00
FBgn0030792	CG4789	1285	0.59	2.29E-05	-0.02	8.66E-01	0.00	1.00E+00
FBgn0039013	CG4813	494	0.59	4.01E-05	-0.03	8.17E-01	0.01	1.00E+00
FBgn0037999	CG4860	453	0.67	2.87E-04	-0.04	7.15E-01	0.00	1.00E+00
FBgn0030803	CG4880	886	-0.73	1.11E-10	-0.08	2.48E-01	0.00	1.00E+00
FBgn0035959	CG4911	926	-0.72	6.09E-06	0.00	9.75E-01	0.00	1.00E+00
FBgn0036587	CG4950	63	-0.37	1.42E-01	-0.05	5.76E-01	-1.06	3.07E-03
FBgn0036579	CG5027	367	0.81	2.00E-08	-0.02	8.81E-01	-0.01	1.00E+00
FBgn0036576	CG5151	885	-0.67	4.99E-04	-0.08	3.45E-01	0.00	1.00E+00
FBgn0038943	CG5391	400	-0.78	3.04E-03	3.62	7.63E-72	0.01	9.15E-01
FBgn0032429	CG5446	1410	0.62	1.05E-06	0.00	9.99E-01	0.00	1.00E+00
FBgn0039430	CG5455	141	-0.01	9.85E-01	-0.87	5.09E-05	-2.03	7.80E-21
FBgn0036760	CG5567	232	0.60	1.79E-03	0.46	6.67E-04	0.03	4.91E-01
FBgn0036975	CG5618	404	0.94	3.40E-05	-0.02	8.64E-01	0.00	1.00E+00
FBgn0036254	CG5645	551	-1.20	2.36E-10	0.36	8.57E-03	0.01	9.37E-01
FBgn0037082	CG5664	538	-0.61	3.00E-05	0.46	6.71E-05	0.01	1.00E+00
FBgn0032171	CG5846	201	0.69	1.24E-03	0.03	7.79E-01	0.00	1.00E+00
FBgn0025700	CG5885	3676	0.60	7.64E-10	-0.05	5.96E-01	-0.01	1.00E+00
FBgn0038400	CG5903	2203	0.58	1.78E-07	0.13	7.05E-02	0.00	1.00E+00
FBgn0036997	CG5955	8	-0.23	1.55E-01	3.40	5.82E-05	-0.01	1.00E+00
FBgn0034725	CG6044	626	0.16	3.62E-01	0.96	1.39E-17	-0.01	1.00E+00
FBgn0031918	CG6055	37	0.14	5.96E-01	-1.12	5.37E-03	-0.01	1.00E+00
FBgn0036186	CG6071	137	-0.03	9.16E-01	0.73	1.03E-03	0.83	7.55E-04
FBgn0032453	CG6180	4013	0.63	5.57E-09	-0.03	8.04E-01	0.00	1.00E+00
FBgn0032343	CG6201	37	-0.25	3.33E-01	-0.81	1.07E-02	-0.01	1.00E+00
FBgn0038321	CG6218	206	-0.62	1.72E-03	-0.04	6.35E-01	-0.02	7.96E-01
FBgn0033866	CG6280	188	-0.32	9.19E-02	-0.99	2.70E-08	-0.01	1.00E+00
FBgn0037807	CG6293	15	-0.15	5.13E-01	-1.83	4.48E-03	-0.02	7.77E-01
FBgn0036121	CG6310	302	0.31	1.04E-01	-0.53	1.07E-04	-1.17	6.76E-19
FBgn0039464	CG6330	158	0.41	8.87E-02	0.88	6.45E-04	-0.20	1.22E-01
FBgn0033875	CG6357	463	-0.71	3.51E-04	-1.63	2.20E-19	0.00	1.00E+00
FBgn0029690	CG6414	96	0.63	1.99E-02	-1.28	1.82E-03	0.00	1.00E+00
FBgn0036702	CG6512	2297	0.13	2.43E-01	0.63	1.96E-19	0.01	8.51E-01
FBgn0032388	CG6686	1995	-0.61	1.94E-05	0.22	2.61E-02	0.00	1.00E+00
FBgn0032305	CG6700	2874	-0.81	6.27E-07	0.05	5.82E-01	0.00	1.00E+00
FBgn0037915	CG6790	9	0.18	2.26E-01	3.70	8.75E-06	0.00	1.00E+00
FBgn0038290	CG6912	99	-5.89	1.77E-07	4.27	3.46E-14	4.50	3.37E-15
FBgn0036240	CG6928	2325	-0.21	2.85E-01	3.92	1.78E-156	2.87	1.32E-82
FBgn0038972	CG7054	111	-0.57	2.87E-02	2.11	1.30E-26	0.33	4.67E-02

FBgn0038941	CG7080	40	0.15	4.58E-01	4.03	3.25E-20	0.00	1.00E+00
FBgn0037099	CG7173	192	0.15	5.00E-01	-0.65	5.38E-04	-0.52	8.92E-03
FBgn0035861	CG7213	27	0.29	2.45E-01	7.51	2.14E-09	0.00	1.00E+00
FBgn0031976	CG7367	156	-0.70	2.69E-03	-0.17	1.49E-01	-0.02	7.39E-01
FBgn0037135	CG7414	11131	0.59	7.82E-19	0.01	9.52E-01	0.00	1.00E+00
FBgn0035833	CG7565	175	-0.69	2.66E-03	-2.28	1.43E-19	-0.04	5.16E-01
FBgn0040793	CG7630	1562	1.04	1.98E-14	0.05	5.86E-01	0.00	1.00E+00
FBgn0033548	CG7637	973	1.92	5.72E-24	0.02	8.99E-01	0.00	1.00E+00
FBgn0036926	CG7646	134	-1.04	3.22E-05	0.02	8.47E-01	0.00	1.00E+00
FBgn0036929	CG7668	1484	-0.86	1.89E-13	-0.02	8.26E-01	-0.01	9.40E-01
FBgn0038619	CG7685	164	0.63	6.57E-03	0.04	6.43E-01	0.00	1.00E+00
FBgn0036714	CG7692	656	-0.87	8.80E-12	0.04	6.82E-01	0.03	5.49E-01
FBgn0036509	CG7739	1153	-0.64	3.23E-11	-0.05	6.08E-01	-0.01	1.00E+00
FBgn0036124	CG7839	1429	-0.80	6.12E-07	0.09	2.63E-01	0.00	1.00E+00
FBgn0037548	CG7900	41	4.55	2.26E-05	2.11	6.72E-04	0.00	1.00E+00
FBgn0028534	CG7916	16	0.13	3.16E-01	0.04	7.30E-01	-1.26	2.16E-02
FBgn0039737	CG7920	3784	0.65	1.36E-09	-0.26	2.06E-03	0.00	1.00E+00
FBgn0028533	CG7953	31	-0.07	5.70E-01	-3.18	4.57E-04	-0.52	5.46E-02
FBgn0038115	CG7966	139	0.86	3.03E-03	-0.07	4.85E-01	0.01	1.00E+00
FBgn0035260	CG7991	93	0.16	5.45E-01	-1.20	1.07E-04	0.01	1.00E+00
FBgn0037612	CG8112	68	0.33	1.87E-01	-0.92	1.04E-02	-0.03	7.02E-01
FBgn0034010	CG8157	91	0.13	5.33E-01	-3.51	4.35E-08	0.00	1.00E+00
FBgn0034011	CG8160	19	0.19	1.39E-01	-0.66	5.32E-02	0.00	1.00E+00
FBgn0034030	CG8192	167	0.76	5.57E-04	-0.26	6.59E-02	0.00	1.00E+00
FBgn0034033	CG8204	166	0.65	4.51E-03	0.01	9.09E-01	0.00	1.00E+00
FBgn0034143	CG8303	208	0.46	3.10E-02	0.69	2.01E-04	0.00	1.00E+00
FBgn0032001	CG8360	132	0.71	2.84E-03	0.03	7.61E-01	0.00	1.00E+00
FBgn0034067	CG8399	2181	0.73	4.88E-15	0.00	9.75E-01	0.00	1.00E+00
FBgn0037754	CG8500	73	-0.69	8.60E-03	-1.78	1.52E-04	-0.03	6.54E-01
FBgn0035791	CG8539	9	-0.06	7.27E-01	0.83	2.63E-02	0.00	1.00E+00
FBgn0035714	CG8549	532	-0.44	1.74E-02	0.60	6.84E-05	0.00	1.00E+00
FBgn0036386	CG8833	390	-0.60	2.98E-04	0.03	8.06E-01	0.01	9.13E-01
FBgn0038404	CG8925	35	-0.18	4.46E-01	0.59	3.32E-02	0.00	1.00E+00
FBgn0038405	CG8927	52	-0.12	6.41E-01	2.02	1.81E-04	0.74	2.16E-02
FBgn0028920	CG8997	28	-0.10	5.51E-01	-5.54	2.35E-06	-0.77	3.60E-02
FBgn0040931	CG9034	454	1.08	6.35E-06	0.05	5.91E-01	0.00	1.00E+00
FBgn0030610	CG9065	223	1.09	7.84E-06	0.38	2.24E-02	0.00	1.00E+00
FBgn0030716	CG9170	1167	0.00	9.77E-01	0.67	9.06E-26	0.01	9.27E-01
FBgn0035181	CG9205	889	1.09	7.19E-17	0.07	3.38E-01	0.00	1.00E+00
FBgn0030669	CG9240	519	0.58	4.27E-05	0.07	3.97E-01	0.00	1.00E+00
FBgn0032925	CG9246	1988	-0.61	5.99E-06	0.06	4.73E-01	0.00	1.00E+00
FBgn0038179	CG9312	150	0.53	2.53E-02	-0.70	4.84E-03	-0.21	1.14E-01
FBgn0032889	CG9331	2348	0.64	4.33E-13	-0.05	5.25E-01	-0.07	1.62E-01
FBgn0032897	CG9336	285	1.72	2.22E-08	-0.01	8.95E-01	0.01	1.00E+00
FBgn0032899	CG9338	208	1.66	1.58E-07	-0.12	2.34E-01	0.00	1.00E+00
FBgn0030569	CG9411	168	-0.21	4.11E-01	0.90	2.91E-04	0.00	1.00E+00
FBgn0037721	CG9427	1394	0.88	9.72E-07	0.00	9.73E-01	0.00	1.00E+00
FBgn0037749	CG9471	643	0.73	3.50E-06	0.10	2.20E-01	0.00	1.00E+00
FBgn0030588	CG9521	33	-0.09	7.33E-01	1.59	1.95E-04	0.33	7.06E-02
FBgn0031824	CG9547	243	0.77	3.20E-04	0.04	7.14E-01	0.00	1.00E+00
FBgn0034184	CG9646	708	-0.15	4.09E-01	0.62	8.27E-08	0.00	1.00E+00
FBgn0029939	CG9650	337	-0.32	1.09E-01	-0.61	9.83E-04	0.00	1.00E+00
FBgn0030160	CG9691	853	0.65	4.41E-04	0.03	7.54E-01	-0.01	1.00E+00
FBgn0039754	CG9747	717	1.09	4.41E-12	-0.51	1.11E-04	-0.01	1.00E+00
FBgn0024248	chico	1054	-0.76	1.68E-13	-0.08	2.97E-01	0.01	1.00E+00
FBgn0000307	chif	2908	-0.58	8.33E-04	0.04	6.23E-01	0.00	1.00E+00
FBgn0086758	chinmo	519	0.32	1.69E-01	0.72	1.56E-03	0.35	4.97E-02
FBgn0045761	CHKov1	1347	-0.44	8.28E-04	0.59	8.77E-09	0.00	1.00E+00
FBgn0039328	CHKov2	325	-0.17	4.44E-01	1.30	1.30E-19	0.00	1.00E+00
FBgn0028387	chm	727	-0.61	1.26E-07	-0.03	7.48E-01	0.00	1.00E+00
FBgn0035589	CHMP2B	224	0.81	1.72E-04	0.03	7.93E-01	0.00	1.00E+00
FBgn0043002	Chrac-14	373	0.65	1.59E-03	0.07	4.13E-01	0.00	1.00E+00
FBgn0036165	chrb	3380	-0.90	1.39E-05	-0.05	5.80E-01	-0.01	1.00E+00
FBgn0022702	Cht2	3503	0.66	5.55E-09	-0.06	4.38E-01	-0.01	8.73E-01
FBgn0038180	Cht5	1359	0.60	1.59E-03	0.04	6.63E-01	0.00	1.00E+00
FBgn0035398	Cht7	1066	0.58	3.91E-03	-0.05	5.99E-01	0.01	9.27E-01
FBgn0004859	ci	8944	-1.01	3.99E-10	-0.05	5.37E-01	0.00	1.00E+00
FBgn0001977	CIAPIN1	1526	0.20	1.04E-01	0.71	1.61E-18	0.00	1.00E+00
FBgn0026084	cib	15779	-0.64	2.76E-06	0.06	4.84E-01	-0.01	1.00E+00
FBgn0015024	CkIalpha	6805	-1.20	3.97E-18	-0.03	7.82E-01	0.00	1.00E+00
FBgn0000259	CkIIbeta	4366	-0.71	2.16E-16	0.01	9.62E-01	0.00	1.00E+00
FBgn0000318	cl	1966	1.07	9.57E-13	0.11	1.85E-01	-0.01	1.00E+00
FBgn0052251	Claspin	1098	-0.97	1.62E-05	0.02	8.37E-01	0.00	1.00E+00
FBgn0259152	Clbn	1163	-0.65	2.13E-07	0.52	2.71E-08	0.00	1.00E+00
FBgn0020503	CLIP-190	1351	-0.94	5.34E-09	0.01	9.54E-01	0.00	1.00E+00
FBgn0035767	Cln7	1086	0.63	1.01E-04	-0.09	2.90E-01	0.00	1.00E+00
FBgn0026255	clumsy	768	-0.05	8.43E-01	1.88	6.29E-37	0.10	1.79E-01
FBgn0034802	CNBP	42330	-0.99	2.65E-17	0.03	7.89E-01	0.00	1.00E+00
FBgn0264077	Cnx14D	13	0.08	7.33E-01	6.73	7.83E-07	0.00	1.00E+00

FBgn0010105	comm	299	0.84	5.61E-06	-0.03	7.35E-01	0.00	1.00E+00
FBgn0039994	conu	1969	-0.92	1.92E-15	0.03	7.39E-01	0.00	1.00E+00
FBgn0033192	Corin	24	-0.10	7.04E-01	-1.49	7.09E-03	0.00	1.00E+00
FBgn0030028	Corp	23	0.18	4.74E-01	0.74	1.54E-02	-0.01	1.00E+00
FBgn0032833	COX4	2941	0.86	5.76E-08	0.12	1.56E-01	0.00	1.00E+00
FBgn0019624	COX5A	2606	1.17	1.83E-09	0.05	5.56E-01	0.00	1.00E+00
FBgn0031830	COX5B	1591	0.59	1.91E-06	0.00	9.88E-01	-0.02	7.52E-01
FBgn0031066	COX6B	2997	0.78	3.12E-11	-0.06	5.05E-01	-0.01	1.00E+00
FBgn0040529	COX7A	3160	1.11	4.94E-14	-0.01	9.48E-01	0.00	1.00E+00
FBgn0263911	COX8	1195	0.59	1.97E-04	0.02	8.62E-01	0.00	1.00E+00
FBgn0053302	Cpr31A	46	1.71	3.35E-06	0.02	8.71E-01	0.00	1.00E+00
FBgn0028871	Cpr35B	60	1.28	2.13E-04	0.00	9.89E-01	-0.01	1.00E+00
FBgn0033598	Cpr47Eb	67	1.16	1.59E-03	0.05	6.06E-01	0.00	1.00E+00
FBgn0033602	Cpr47Ee	24	-0.03	8.88E-01	-1.69	4.84E-03	0.00	1.00E+00
FBgn0033730	Cpr49Ag	381	0.92	1.89E-07	-0.07	3.83E-01	0.00	1.00E+00
FBgn0033731	Cpr49Ah	539	0.81	1.36E-03	1.02	1.13E-04	0.16	1.61E-01
FBgn0033942	Cpr51A	266	0.96	8.10E-05	-0.51	9.48E-03	0.00	1.00E+00
FBgn0034499	Cpr56F	33	-0.17	4.91E-01	-2.07	2.64E-04	-0.01	1.00E+00
FBgn0035737	Cpr65Ec	171	0.83	1.01E-03	-0.33	4.57E-02	-0.21	1.14E-01
FBgn0052029	Cpr66D	478	1.87	5.31E-25	-0.03	8.00E-01	0.00	1.00E+00
FBgn0036109	Cpr67Fa2	86	0.27	2.67E-01	-0.09	3.18E-01	-0.66	5.64E-03
FBgn0036617	Cpr72Ea	13	0.83	6.43E-03	0.03	7.54E-01	0.00	1.00E+00
FBgn0037114	Cpr78E	355	0.71	9.83E-03	-0.59	2.57E-02	-0.02	8.40E-01
FBgn0042701	CR12628	15	0.32	1.58E-01	1.70	3.97E-03	0.03	7.10E-01
FBgn0040928	CR15345	118	-0.66	4.31E-03	0.00	9.77E-01	0.00	1.00E+00
FBgn0262532	CR43086	36	0.01	9.46E-01	5.51	5.98E-22	3.08	3.23E-06
FBgn0263093	CR43361	426	-0.03	8.89E-01	-1.74	1.53E-30	0.00	1.00E+00
FBgn0263744	CR43669	21	-0.86	5.34E-03	0.04	7.25E-01	0.00	1.00E+00
FBgn0035636	Cralbp	80	0.73	3.28E-03	-0.10	3.25E-01	0.00	1.00E+00
FBgn0004396	CrebA	1973	-0.77	1.03E-05	0.00	9.83E-01	0.00	1.00E+00
FBgn0023023	CRMP	259	-0.13	5.66E-01	-1.04	7.11E-10	0.00	1.00E+00
FBgn0015924	crq	883	-0.78	1.85E-12	-0.06	4.91E-01	0.00	1.00E+00
FBgn0036746	Crtc	1137	-0.88	5.35E-06	0.04	7.12E-01	0.00	1.00E+00
FBgn0000384	cta	945	-0.64	6.04E-04	0.02	8.88E-01	0.00	1.00E+00
FBgn0062412	Ctr1B	34	0.02	8.78E-01	-0.82	3.92E-02	-0.07	4.03E-01
FBgn0260932	cuff	181	-0.59	9.91E-04	0.34	1.92E-02	0.00	1.00E+00
FBgn0032956	Cul2	6827	-0.48	5.97E-05	3.49	0.00E+00	0.01	1.00E+00
FBgn0261268	Cul3	3133	-0.62	1.96E-10	-0.02	9.07E-01	0.00	1.00E+00
FBgn0035880	Culd	2104	-0.15	5.49E-01	3.94	7.19E-150	5.04	1.82E-247
FBgn0031689	Cyp28d1	91	1.86	1.03E-03	-0.16	2.56E-01	0.00	1.00E+00
FBgn0001992	Cyp303a1	68	-0.99	1.46E-03	0.02	8.88E-01	0.02	9.40E-01
FBgn0034756	Cyp6d2	381	0.01	9.79E-01	4.75	2.83E-216	0.02	7.02E-01
FBgn0039006	Cyp6d4	137	0.36	1.38E-01	1.42	6.67E-10	0.00	1.00E+00
FBgn0025454	Cyp6g1	147	0.24	3.07E-01	-1.83	1.59E-04	-0.01	1.00E+00
FBgn0031126	Cyp6v1	1037	-1.19	1.46E-27	-0.18	5.37E-03	0.00	1.00E+00
FBgn0000411	D	14	0.19	2.73E-01	-0.62	4.04E-02	0.01	1.00E+00
FBgn0005677	dac	806	-0.13	4.87E-01	-0.95	7.73E-13	0.03	5.06E-01
FBgn0263852	Dad1	1589	1.13	4.02E-14	0.00	9.86E-01	-0.01	1.00E+00
FBgn0263930	dally	6391	-0.67	2.37E-07	0.06	4.68E-01	0.00	1.00E+00
FBgn0262636	dati	43	-0.07	7.98E-01	3.80	3.07E-10	5.11	4.48E-18
FBgn0031820	Daxx	2065	-0.59	3.50E-06	-0.02	8.83E-01	0.01	1.00E+00
FBgn0067779	dbr	1696	-0.84	1.08E-11	0.01	9.73E-01	0.00	1.00E+00
FBgn0002413	dco	3330	-0.72	1.41E-14	-0.01	9.24E-01	0.00	1.00E+00
FBgn0036534	DCP2	6445	-0.99	5.36E-08	0.03	7.18E-01	0.00	1.00E+00
FBgn0029067	Dd	965	-0.87	1.81E-10	-0.02	8.60E-01	0.00	1.00E+00
FBgn0086251	del	642	-0.93	2.75E-08	-0.05	5.91E-01	0.00	1.00E+00
FBgn0267972	Der-1	1024	0.64	1.65E-07	0.03	7.60E-01	0.00	1.00E+00
FBgn0022893	Df31	48354	-0.63	6.61E-10	0.02	8.64E-01	0.00	1.00E+00
FBgn0013812	Dhc93AB	47	-0.10	7.32E-01	0.83	4.04E-03	0.00	1.00E+00
FBgn0000449	dib	86	-0.60	1.66E-02	-1.67	3.76E-06	-0.04	5.19E-01
FBgn0011274	Dif	323	-0.45	3.54E-02	2.57	3.24E-38	0.00	1.00E+00
FBgn0000459	disco	168	-0.02	9.57E-01	-0.73	2.21E-03	0.02	8.78E-01
FBgn0285879	disco-r	318	0.11	6.79E-01	-1.45	2.90E-11	0.04	4.80E-01
FBgn0263106	DnaJ-1	4644	0.13	5.57E-01	-0.72	2.47E-05	0.00	1.00E+00
FBgn0286075	DNAlig3	384	-0.23	1.73E-01	0.73	5.92E-14	0.00	1.00E+00
FBgn0030506	DNAlig4	262	-0.19	3.68E-01	0.74	2.66E-08	0.00	1.00E+00
FBgn0002891	DNApol-zeta	1022	-0.31	3.75E-02	0.88	1.75E-15	0.02	6.30E-01
FBgn0035542	DOR	485	-0.62	6.59E-03	0.00	9.77E-01	0.00	1.00E+00
FBgn0024558	Dph5	1749	0.65	2.00E-07	0.09	2.15E-01	0.00	1.00E+00
FBgn0037295	dpr16	116	2.16	3.85E-14	0.00	9.71E-01	-0.01	9.39E-01
FBgn0053196	dpy	6720	1.12	5.51E-07	0.63	1.77E-03	0.01	1.00E+00
FBgn0032293	Dpy-30L1	556	0.88	3.69E-06	-0.01	9.49E-01	0.00	1.00E+00
FBgn0260006	drd	87	0.15	5.72E-01	1.73	4.49E-05	0.01	1.00E+00
FBgn0004638	drk	3220	-0.61	7.50E-08	-0.02	8.51E-01	0.00	1.00E+00
FBgn0024244	drm	2325	-0.71	1.13E-07	-0.15	8.06E-02	-0.01	1.00E+00
FBgn0278608	Dsp1	6983	-0.58	7.34E-05	0.00	9.82E-01	0.00	1.00E+00
FBgn0261799	dsx-c73A	101	1.06	6.38E-06	0.37	2.38E-02	0.00	1.00E+00
FBgn0027101	Dyrk3	2604	-0.88	1.38E-09	-0.03	7.75E-01	0.00	1.00E+00
FBgn0002592	E(spl)m2-BFM	1109	0.65	6.60E-06	-0.12	1.52E-01	0.00	1.00E+00

FBgn0002629	E(spl)m4-BFM	800	0.72	3.01E-07	0.05	5.64E-01	0.09	1.63E-01
FBgn0002732	E(spl)malpha-BFM	2631	0.76	1.39E-05	-0.01	9.10E-01	0.00	1.00E+00
FBgn0002735	E(spl)mgamma-HLH	148	0.65	4.39E-03	0.05	5.80E-01	0.01	8.87E-01
FBgn0011766	E2f1	4380	-0.84	3.21E-10	0.01	9.30E-01	0.01	1.00E+00
FBgn0069242	eca	731	0.68	1.04E-03	0.00	9.98E-01	-0.01	1.00E+00
FBgn0033879	Echs1	3518	0.92	3.48E-26	-0.02	8.64E-01	0.00	1.00E+00
FBgn0260746	Ect3	339	-1.92	2.07E-19	0.04	7.15E-01	-0.01	1.00E+00
FBgn0028737	eEF1beta	17684	0.95	5.64E-23	-0.07	3.37E-01	0.00	1.00E+00
FBgn0029176	eEF1gamma	47067	0.69	1.56E-30	-0.04	6.50E-01	0.00	1.00E+00
FBgn0034883	Eglp2	10	0.03	8.59E-01	1.95	2.90E-03	0.19	1.44E-01
FBgn0037270	eIF3f1	6584	0.80	1.53E-14	-0.04	7.25E-01	0.00	1.00E+00
FBgn0022023	eIF3h	7150	0.90	6.93E-21	0.00	9.80E-01	0.00	1.00E+00
FBgn0020660	eIF4B	389	-0.77	7.87E-03	-0.12	2.19E-01	-0.07	3.37E-01
FBgn0053100	eIF4EHP	1127	-1.07	3.14E-18	0.03	7.43E-01	0.00	1.00E+00
FBgn0023213	eIF4G1	11399	-0.77	1.42E-10	-0.03	7.88E-01	0.00	1.00E+00
FBgn0262734	eIF4H1	7393	-1.47	7.70E-14	0.01	9.18E-01	0.00	1.00E+00
FBgn0030719	eIF5	87	-2.37	1.38E-05	0.03	7.39E-01	0.00	1.00E+00
FBgn0034915	eIF6	2129	0.36	8.36E-03	0.83	2.03E-15	0.00	1.00E+00
FBgn0004858	elB	1567	-0.77	1.52E-09	0.04	6.25E-01	0.00	1.00E+00
FBgn0037534	ELOVL	41	0.60	2.45E-02	-0.09	4.12E-01	0.00	1.00E+00
FBgn0062440	EMRE	456	0.87	1.66E-05	0.01	9.36E-01	0.00	1.00E+00
FBgn0000579	Eno	10736	0.65	1.05E-11	-0.03	7.79E-01	-0.01	1.00E+00
FBgn0264693	ens	3968	-0.63	2.15E-06	0.01	9.18E-01	0.00	1.00E+00
FBgn0036319	Ent3	19	-0.14	4.83E-01	-0.83	1.87E-02	-0.01	1.00E+00
FBgn0025936	Eph	4729	-0.81	1.92E-08	-0.07	4.30E-01	0.00	1.00E+00
FBgn0040324	Ephrin	2393	-0.62	2.73E-03	-0.01	9.53E-01	0.00	1.00E+00
FBgn0005660	Ets21C	87	0.15	5.10E-01	2.51	3.68E-04	0.00	1.00E+00
FBgn0033668	exp	166	-0.65	1.72E-03	-0.06	4.78E-01	0.00	1.00E+00
FBgn0034529	FAM21	494	-0.58	1.42E-05	0.02	8.90E-01	0.01	1.00E+00
FBgn0032428	Fam92	20	0.07	7.33E-01	3.79	5.35E-11	0.00	1.00E+00
FBgn0042627	FASN2	15	0.06	6.87E-01	0.67	4.83E-02	0.10	2.91E-01
FBgn0034999	Fatp3	93	0.25	3.03E-01	-1.49	1.09E-03	-0.09	3.00E-01
FBgn0014163	fax	30703	-0.92	3.44E-16	-0.10	1.77E-01	0.00	1.00E+00
FBgn0000639	Fbp1	20462	0.58	1.65E-02	-0.05	6.80E-01	0.00	1.00E+00
FBgn0004897	fd96Ca	39	0.18	2.26E-01	-2.52	9.80E-08	0.00	1.00E+00
FBgn0004898	fd96Cb	154	1.83	1.09E-03	-1.96	8.37E-05	0.01	1.00E+00
FBgn0011768	Fdh	3119	1.45	1.89E-32	0.12	9.98E-02	0.02	6.08E-01
FBgn0015221	Fer2LCH	4994	0.62	1.05E-16	0.11	6.21E-02	0.00	1.00E+00
FBgn0039969	Fis1	1106	-0.59	9.95E-05	-0.02	8.95E-01	0.00	1.00E+00
FBgn0000658	fj	5209	-0.61	3.71E-11	0.11	9.65E-02	0.00	1.00E+00
FBgn0013954	Fkbp12	9074	1.03	6.70E-36	0.08	2.51E-01	0.00	1.00E+00
FBgn0033806	FLASH	452	-0.84	2.02E-07	0.04	6.52E-01	0.00	1.00E+00
FBgn0028734	Fmr1	5727	-0.78	5.68E-09	0.05	5.79E-01	0.00	1.00E+00
FBgn0286222	Fum1	4193	0.72	1.52E-10	-0.04	7.12E-01	0.00	1.00E+00
FBgn0039932	fuss	37	-0.01	9.66E-01	2.22	1.05E-05	0.00	1.00E+00
FBgn0001085	fz	2807	-0.62	1.53E-06	-0.08	3.18E-01	0.00	1.00E+00
FBgn0031275	GABA-B-R3	12	0.31	1.46E-01	3.04	4.22E-05	0.00	1.00E+00
FBgn0033153	Gadd45	924	-0.03	8.97E-01	1.85	2.07E-41	0.00	1.00E+00
FBgn0001122	Galphao	883	-0.93	4.45E-10	-0.02	8.19E-01	0.00	1.00E+00
FBgn0004435	Galphaq	656	-1.67	8.33E-15	0.00	9.86E-01	0.00	1.00E+00
FBgn0001123	Galphas	2681	-0.69	1.58E-16	-0.03	8.08E-01	0.00	1.00E+00
FBgn0001091	Gapdh1	5452	0.68	3.17E-08	0.03	7.91E-01	0.00	1.00E+00
FBgn0001092	Gapdh2	16172	0.72	1.33E-21	0.08	2.03E-01	0.00	1.00E+00
FBgn0032223	GATAd	1049	-0.75	4.92E-07	-0.05	6.16E-01	0.00	1.00E+00
FBgn0001105	Gbeta13F	6116	-1.18	2.14E-13	-0.02	8.45E-01	0.00	1.00E+00
FBgn0040319	Gclc	466	-0.10	6.52E-01	0.86	6.23E-10	0.00	1.00E+00
FBgn0020388	Gcn5	1584	0.01	9.38E-01	1.05	8.06E-33	0.01	1.00E+00
FBgn0000808	gd	21	0.13	5.32E-01	3.47	7.24E-13	0.00	1.00E+00
FBgn0033081	geminin	1895	0.66	1.64E-06	-0.02	9.04E-01	0.00	1.00E+00
FBgn0250823	gish	4407	-0.94	4.34E-13	0.03	7.82E-01	0.00	1.00E+00
FBgn0015229	glec	1116	-0.61	3.40E-05	0.02	8.88E-01	-0.01	1.00E+00
FBgn0004913	Gnf1	1884	-0.64	1.25E-06	-0.01	9.54E-01	0.01	1.00E+00
FBgn0263048	Gpdh3	671	-0.71	1.65E-07	0.05	5.72E-01	0.01	1.00E+00
FBgn0260798	Gprk1	1444	-0.93	2.42E-16	-0.06	4.44E-01	0.00	1.00E+00
FBgn0035167	Gr61a	111	0.64	1.02E-02	1.25	3.97E-08	0.00	1.00E+00
FBgn0039494	grass	168	0.95	6.23E-04	-0.03	7.66E-01	0.00	1.00E+00
FBgn0001137	grk	60	-1.88	3.50E-06	0.01	9.09E-01	-0.01	1.00E+00
FBgn0019982	Gs1l	293	0.61	5.31E-03	0.00	9.95E-01	0.00	1.00E+00
FBgn0001148	gsb	14	0.09	6.04E-01	-1.84	2.89E-03	0.00	1.00E+00
FBgn0001147	gsb-n	13	0.07	6.91E-01	-1.55	4.35E-03	0.00	1.00E+00
FBgn0001149	GstD1	10655	1.18	7.26E-22	0.08	3.21E-01	-0.01	1.00E+00
FBgn0010039	GstD3	959	-1.17	1.90E-10	0.63	3.50E-07	0.00	1.00E+00
FBgn0038020	GstD9	723	-0.53	5.19E-03	0.89	3.62E-10	0.02	7.52E-01
FBgn0034335	GstE1	1270	0.50	2.32E-02	5.48	0.00E+00	0.62	1.95E-05
FBgn0027590	GstE12	2243	0.80	1.39E-08	0.06	5.13E-01	0.00	1.00E+00
FBgn0063498	GstE2	76	0.03	8.52E-01	6.89	9.09E-39	0.00	1.00E+00
FBgn0063495	GstE5	41	0.36	1.44E-01	1.34	9.60E-05	-0.01	1.00E+00
FBgn0063494	GstE6	374	0.19	4.42E-01	4.52	2.60E-102	0.80	1.67E-03
FBgn0063492	GstE8	106	0.14	5.85E-01	1.66	7.07E-11	0.63	1.24E-02

FBgn0050000	GstT1	515	-0.02	9.28E-01	1.05	3.81E-19	0.00	1.00E+00
FBgn0031117	GstT3	109	0.98	3.51E-04	0.02	8.80E-01	-0.03	6.08E-01
FBgn0026238	gus	2692	-0.99	3.39E-18	-0.01	9.44E-01	0.00	1.00E+00
FBgn0039936	Gyf	5384	-0.60	9.10E-09	-0.05	5.59E-01	0.00	1.00E+00
FBgn0016660	H15	67	0.29	1.75E-01	-1.81	1.80E-04	0.01	1.00E+00
FBgn0032394	Hacd1	670	0.71	1.68E-07	-0.22	1.37E-02	0.00	1.00E+00
FBgn0034488	Hacl	2789	0.59	1.23E-11	-0.03	7.67E-01	0.00	1.00E+00
FBgn0286508	Had1	310	0.66	8.55E-03	-0.01	9.16E-01	0.01	1.00E+00
FBgn0033949	Had2	63	0.21	4.30E-01	-0.31	7.20E-02	-0.59	2.15E-02
FBgn0046706	Haspin	1098	-1.00	3.56E-13	0.01	9.18E-01	-0.01	9.13E-01
FBgn0051119	HDAC11	108	-0.87	4.32E-04	0.13	3.37E-01	-0.01	1.00E+00
FBgn0011224	heph	19761	-0.80	6.76E-07	-0.01	9.33E-01	0.00	1.00E+00
FBgn0001185	her	823	-0.65	6.24E-10	0.00	9.90E-01	0.00	1.00E+00
FBgn0031459	HINT1	2526	1.22	1.99E-18	0.04	6.85E-01	0.00	1.00E+00
FBgn0029676	HIP-R	1449	-1.00	1.27E-11	0.02	8.38E-01	-0.01	1.00E+00
FBgn0010228	HmgZ	5046	-1.13	3.34E-40	-0.06	4.70E-01	0.00	1.00E+00
FBgn0035160	hng3	952	0.83	6.58E-08	0.03	7.51E-01	-0.02	7.72E-01
FBgn0286786	hoip	2078	0.67	3.30E-05	0.06	4.66E-01	0.00	1.00E+00
FBgn0025777	homer	1227	-0.59	2.79E-06	-0.01	9.45E-01	0.00	1.00E+00
FBgn0039019	HP1c	924	0.74	1.49E-08	0.02	8.82E-01	-0.01	1.00E+00
FBgn0035829	HP4	943	0.86	1.33E-08	-0.01	9.47E-01	0.00	1.00E+00
FBgn0036992	Hpd	47	-0.06	8.29E-01	1.06	2.78E-02	0.00	1.00E+00
FBgn0062928	hpRNA:CR33940	1575	0.04	8.63E-01	0.08	3.50E-01	-0.63	6.35E-05
FBgn0261239	Hr39	2570	-0.62	2.60E-04	0.03	7.29E-01	0.00	1.00E+00
FBgn0004838	Hrb27C	27370	-0.74	5.65E-06	0.00	9.93E-01	0.00	1.00E+00
FBgn0015949	hrg	4460	-0.65	6.58E-08	-0.01	9.30E-01	0.00	1.00E+00
FBgn0001217	Hsc70-2	69	6.71	3.42E-10	-0.17	2.55E-01	-0.01	1.00E+00
FBgn0266599	Hsc70-4	117280	0.62	2.13E-13	-0.02	8.37E-01	-0.01	1.00E+00
FBgn0001225	Hsp26	9822	1.22	1.08E-09	-0.76	2.25E-05	-0.01	9.36E-01
FBgn0015245	Hsp60A	10641	0.72	1.47E-15	0.03	7.32E-01	0.00	1.00E+00
FBgn0001229	Hsp67Bc	154	0.75	5.60E-03	-0.25	1.02E-01	1.19	2.82E-04
FBgn0001230	Hsp68	4283	2.04	3.72E-05	-0.23	1.53E-01	0.00	1.00E+00
FBgn0013276	Hsp70Ab	244	1.56	5.11E-05	-0.04	6.54E-01	0.00	1.00E+00
FBgn0013278	Hsp70Bb	477	3.65	6.59E-06	-0.04	6.85E-01	0.00	1.00E+00
FBgn0013279	Hsp70Bc	336	0.14	5.78E-01	-0.60	2.76E-02	0.00	1.00E+00
FBgn0061198	HSPC300	346	0.67	1.15E-03	0.03	7.98E-01	0.00	1.00E+00
FBgn0001235	hth	8836	-1.34	8.95E-12	-0.03	7.37E-01	0.00	1.00E+00
FBgn0010389	htl	655	-0.47	5.38E-04	-0.68	7.42E-11	-0.03	5.57E-01
FBgn0033968	hui	830	1.30	1.52E-31	-0.25	1.07E-03	-0.40	2.25E-06
FBgn0036556	hzg	1250	-0.84	3.30E-07	-0.04	6.88E-01	0.00	1.00E+00
FBgn0286204	ich	15	-0.15	4.49E-01	0.76	3.76E-02	0.82	3.10E-02
FBgn0001248	Idh	11385	0.64	1.66E-18	-0.41	1.55E-11	-0.79	3.46E-41
FBgn0036690	Ilp8	61	0.10	4.72E-01	7.14	7.50E-17	0.00	1.00E+00
FBgn0285926	Imp	2083	-0.92	7.76E-06	0.01	9.32E-01	0.00	1.00E+00
FBgn0001255	ImpE3	1673	0.60	8.75E-03	-0.03	8.00E-01	-0.01	1.00E+00
FBgn0001256	ImpL1	53	0.18	4.69E-01	-2.17	5.55E-05	-0.01	1.00E+00
FBgn0001263	inaD	152	-1.09	6.41E-06	-0.33	1.27E-01	-0.05	5.16E-01
FBgn0039459	IntS12	266	0.59	4.56E-03	0.12	1.91E-01	0.84	3.22E-06
FBgn0262117	IntS3	2249	-0.72	5.69E-13	-0.03	8.06E-01	0.00	1.00E+00
FBgn0027106	Inx7	34	1.67	7.61E-05	0.00	9.88E-01	-0.01	1.00E+00
FBgn0259683	Ir40a	56	0.12	4.36E-01	5.29	3.59E-25	0.01	1.00E+00
FBgn0050081	Ir51b	36	-0.63	1.99E-02	0.00	9.89E-01	0.00	1.00E+00
FBgn0011774	Irbp	458	-0.04	8.60E-01	1.22	2.35E-45	0.02	6.08E-01
FBgn0036126	Irbp18	342	-0.22	2.45E-01	1.38	2.54E-33	0.00	1.00E+00
FBgn0037637	IscU	1069	-0.24	1.13E-01	1.08	5.20E-30	0.00	1.00E+00
FBgn0034005	ItgaPS4	26	0.83	6.27E-03	0.28	1.34E-01	0.00	1.00E+00
FBgn0010053	Jheh1	254	0.43	2.53E-02	-0.67	4.97E-05	0.00	1.00E+00
FBgn0028424	JhI-26	62	-0.06	8.21E-01	1.56	1.78E-03	0.00	1.00E+00
FBgn0020906	Jon25Bi	21	-0.13	2.46E-01	-2.69	6.00E-03	-0.01	1.00E+00
FBgn0031654	Jon25Bii	14	-0.12	2.89E-01	-1.07	2.92E-02	-0.01	1.00E+00
FBgn0035665	Jon65Aiii	69	-0.02	8.87E-01	-3.27	4.09E-03	-0.02	9.06E-01
FBgn0250815	Jon65Aiv	95	-0.01	9.39E-01	-5.49	2.64E-04	-0.02	1.00E+00
FBgn0025820	JTBR	391	0.66	1.51E-04	0.04	6.21E-01	0.00	1.00E+00
FBgn0001296	kar	177	0.71	1.82E-03	0.08	3.88E-01	-0.01	1.00E+00
FBgn0015399	kek1	2777	-0.75	1.30E-07	-0.10	1.92E-01	0.15	8.10E-02
FBgn0039925	Kif3C	10	-0.20	3.61E-01	0.22	1.84E-01	1.55	9.41E-03
FBgn0001323	knrl	445	-0.98	3.52E-07	0.18	9.48E-02	0.00	1.00E+00
FBgn0034098	krimp	58	0.81	5.98E-03	0.02	8.22E-01	0.53	3.30E-02
FBgn0040206	krz	1877	-0.74	4.98E-07	0.02	8.67E-01	0.00	1.00E+00
FBgn0040890	ksh	432	0.80	1.06E-04	0.01	9.07E-01	0.00	1.00E+00
FBgn0041627	Ku80	500	-0.25	7.23E-02	1.10	1.85E-41	0.02	6.49E-01
FBgn0036667	kud	749	0.74	1.15E-04	0.01	9.30E-01	0.00	1.00E+00
FBgn0259984	kuz	2737	-0.59	1.26E-03	-0.04	6.48E-01	0.00	1.00E+00
FBgn0040153	l(1)G0469	354	-0.48	2.80E-02	-0.66	2.67E-03	-0.01	1.00E+00
FBgn0284251	l(2)05287	1676	-0.58	6.07E-06	0.03	8.01E-01	0.00	1.00E+00
FBgn0002031	l(2)37Cc	3950	0.63	7.33E-12	0.12	4.75E-02	0.00	1.00E+00
FBgn0002121	l(2)gl	5956	-0.66	9.23E-06	-0.03	7.39E-01	0.00	1.00E+00
FBgn0284244	l(2)k05911	1160	-0.03	8.80E-01	0.95	2.58E-13	-0.05	3.65E-01
FBgn0035617	l(3)psg2	1918	-0.65	2.42E-05	0.04	6.68E-01	0.00	1.00E+00

FBgn0008651	lbl	44	0.20	2.70E-01	-2.03	4.28E-05	0.00	1.00E+00
FBgn0001258	Ldh	467	0.64	4.76E-03	1.77	2.03E-15	0.07	3.52E-01
FBgn0016675	Lectin-galC1	18	0.05	7.07E-01	1.81	1.00E-02	0.97	3.10E-02
FBgn0034877	levy	2150	0.87	1.58E-07	0.02	8.52E-01	0.00	1.00E+00
FBgn0025692	Lfg	29	-0.11	6.55E-01	-0.99	2.30E-02	-0.02	8.66E-01
FBgn0039907	lgs	3040	-0.63	1.33E-06	-0.03	7.79E-01	0.00	1.00E+00
FBgn0041111	lilli	1423	-0.65	1.41E-04	-0.01	9.51E-01	0.00	1.00E+00
FBgn0026411	Lim1	66	0.31	1.24E-01	-2.43	3.62E-08	0.01	1.00E+00
FBgn0032253	LManI	47	-0.30	2.32E-01	-0.82	3.71E-03	-0.16	1.60E-01
FBgn0027611	LManII	658	0.16	5.25E-01	-0.98	2.09E-04	-0.03	7.07E-01
FBgn0051044	lncRNA:CR31044	109	-0.62	1.51E-02	-0.06	4.99E-01	0.00	1.00E+00
FBgn0063127	lncRNA:CR33938	820	2.12	4.16E-22	0.14	1.56E-01	0.00	1.00E+00
FBgn0267910	lncRNA:CR34335	7316	1.25	1.68E-05	0.02	8.76E-01	0.00	1.00E+00
FBgn0259993	lncRNA:CR42491	1363	0.61	4.70E-06	0.06	4.52E-01	-0.94	5.20E-17
FBgn0261813	lncRNA:CR42755	10	-0.25	2.06E-01	-0.12	3.37E-01	-2.47	2.02E-03
FBgn0262620	lncRNA:CR43144	765	0.08	7.87E-01	5.09	1.28E-279	0.02	7.45E-01
FBgn0262886	lncRNA:CR43241	230	-1.36	3.84E-09	0.04	7.17E-01	0.00	1.00E+00
FBgn0286827	lncRNA:CR43334	202	0.33	1.75E-01	0.68	2.98E-02	-0.03	7.07E-01
FBgn0263415	lncRNA:CR43461	36	-0.06	8.34E-01	0.61	3.42E-02	0.00	1.00E+00
FBgn0263617	lncRNA:CR43626	25	-0.89	4.25E-03	0.00	9.88E-01	0.00	1.00E+00
FBgn0263626	lncRNA:CR43635	12	0.86	5.82E-03	0.02	8.52E-01	0.00	1.00E+00
FBgn0264721	lncRNA:CR43989	32	-0.04	9.11E-01	0.85	7.12E-03	0.01	1.00E+00
FBgn0264794	lncRNA:CR44024	20	0.03	9.25E-01	2.10	2.05E-06	0.00	1.00E+00
FBgn0264834	lncRNA:CR44042	177	-0.77	1.55E-03	0.63	1.55E-03	0.00	1.00E+00
FBgn0264942	lncRNA:CR44111	42	0.09	5.34E-01	5.99	1.17E-27	0.00	1.00E+00
FBgn0264984	lncRNA:CR44135	23	-0.29	1.06E-01	1.21	3.43E-03	-0.01	1.00E+00
FBgn0265422	lncRNA:CR44334	427	-0.68	3.70E-04	0.20	6.50E-02	0.01	8.66E-01
FBgn0265497	lncRNA:CR44366	18	-0.66	1.58E-02	-0.03	7.38E-01	0.00	1.00E+00
FBgn0265651	lncRNA:CR44458	17	-0.04	8.81E-01	1.51	3.11E-03	-0.01	1.00E+00
FBgn0266049	lncRNA:CR44814	120	-1.88	1.42E-09	0.04	7.13E-01	0.00	1.00E+00
FBgn0266051	lncRNA:CR44816	80	-1.42	2.27E-06	0.00	9.92E-01	-0.01	1.00E+00
FBgn0266414	lncRNA:CR45054	19	0.14	3.83E-01	0.79	3.16E-02	0.00	1.00E+00
FBgn0266549	lncRNA:CR45102	308	-2.24	1.22E-12	0.02	8.91E-01	0.00	1.00E+00
FBgn0266691	lncRNA:CR45181	18	0.87	5.43E-03	0.52	4.50E-02	0.01	9.89E-01
FBgn0267793	lncRNA:CR45232	366	-0.73	3.43E-04	-0.15	1.61E-01	0.00	1.00E+00
FBgn0266866	lncRNA:CR45327	19	-0.60	2.28E-02	-0.03	8.04E-01	-0.01	1.00E+00
FBgn0266958	lncRNA:CR45409	159	-1.00	1.92E-05	-0.03	7.29E-01	0.00	1.00E+00
FBgn0267029	lncRNA:CR45473	135	-0.38	7.22E-02	0.78	1.73E-05	0.01	1.00E+00
FBgn0267073	lncRNA:CR45517	44	-0.04	8.97E-01	2.24	6.58E-11	0.04	5.85E-01
FBgn0267126	lncRNA:CR45566	684	0.92	7.68E-13	0.05	6.05E-01	0.00	1.00E+00
FBgn0267635	lncRNA:CR45973	81	-0.26	3.03E-01	1.44	1.55E-08	0.00	1.00E+00
FBgn0278598	lncRNA:CR46268	190	-1.19	1.56E-06	0.03	7.60E-01	0.00	1.00E+00
FBgn0267585	lncRNA:dntRL	95	-0.03	9.12E-01	1.02	6.17E-06	0.00	1.00E+00
FBgn0022238	lolal	3178	-1.02	7.82E-19	0.05	5.82E-01	0.00	1.00E+00
FBgn0052699	LPCAT	956	-0.61	5.17E-07	0.00	9.93E-01	0.00	1.00E+00
FBgn0028582	lqf	1867	-0.59	1.74E-08	-0.01	9.20E-01	0.00	1.00E+00
FBgn0267861	Maf1	2161	-0.95	1.80E-17	0.00	9.83E-01	0.00	1.00E+00
FBgn0029979	mahe	4391	-0.78	1.42E-04	-0.03	7.25E-01	0.00	1.00E+00
FBgn0032382	Mal-B2	163	0.24	3.10E-01	2.29	9.15E-07	0.00	1.00E+00
FBgn0034282	Mapmodulin	8990	-0.84	1.93E-15	0.05	5.94E-01	0.00	1.00E+00
FBgn0039972	Marf1	212	0.44	4.74E-02	1.26	1.83E-10	-0.01	1.00E+00
FBgn0039914	mav	2998	-0.75	1.06E-16	0.07	3.67E-01	0.00	1.00E+00
FBgn0035811	Mcad	4056	0.66	1.26E-09	-0.26	2.17E-03	0.00	1.00E+00
FBgn0032929	Mcm10	613	-0.83	1.75E-08	0.06	4.99E-01	0.02	7.84E-01
FBgn0284442	Mcm3	2442	0.59	1.94E-05	-0.01	9.16E-01	0.00	1.00E+00
FBgn0032116	Mco1	31	-0.16	5.19E-01	-0.74	2.10E-02	0.00	1.00E+00
FBgn0262782	Mdh1	2243	0.69	7.72E-06	-0.03	7.73E-01	0.00	1.00E+00
FBgn0262559	Mdh2	2464	0.61	1.94E-06	0.05	5.86E-01	0.00	1.00E+00
FBgn0034707	MED16	746	0.29	2.14E-02	-0.58	1.75E-11	0.00	1.00E+00
FBgn0039923	MED26	3695	-0.93	2.24E-08	-0.04	6.74E-01	0.00	1.00E+00
FBgn0039337	MED28	361	0.64	1.35E-03	0.03	7.60E-01	0.00	1.00E+00
FBgn0024556	mEFTu1	2555	0.70	8.55E-10	0.15	4.44E-02	0.00	1.00E+00
FBgn0039851	mey	1124	1.29	3.47E-17	1.26	1.81E-23	1.01	3.55E-15
FBgn0031307	MFS3	3325	0.69	9.89E-07	0.00	9.93E-01	0.00	1.00E+00
FBgn0025814	Mgstl	739	0.87	2.43E-05	-0.03	7.47E-01	0.00	1.00E+00
FBgn0036333	MICAL-like	1637	-0.63	2.14E-13	-0.12	3.63E-02	0.00	1.00E+00
FBgn0261963	mid	203	0.43	4.48E-02	-0.58	3.07E-03	0.01	1.00E+00
FBgn0039250	Mink	2302	-0.60	9.89E-07	-0.06	4.88E-01	0.00	1.00E+00
FBgn0026061	Mipp1	38	1.35	5.54E-04	0.00	9.98E-01	0.00	1.00E+00
FBgn0263112	Mitf	2077	-0.73	4.40E-07	0.00	9.90E-01	0.04	4.11E-01
FBgn0024326	Mkk4	1661	-0.65	1.15E-11	-0.02	9.00E-01	0.00	1.00E+00
FBgn0002774	mle	2065	-0.68	3.04E-14	-0.08	2.61E-01	0.00	1.00E+00
FBgn0259168	mnb	2631	-0.67	4.00E-06	-0.05	6.05E-01	0.00	1.00E+00
FBgn0263241	Mocs1	299	0.02	9.55E-01	1.00	6.99E-12	-0.02	7.07E-01
FBgn0026409	Mpcp2	7899	0.69	8.14E-23	0.05	5.60E-01	0.00	1.00E+00
FBgn0020270	mre11	1699	0.34	1.30E-03	1.11	7.59E-75	0.35	3.50E-07
FBgn0034091	mrj	966	-0.79	8.03E-13	-0.01	9.45E-01	0.00	1.00E+00
FBgn0032456	MRP	3772	0.06	7.35E-01	1.55	5.28E-48	0.00	1.00E+00
FBgn0011787	mRpL12	969	0.91	4.58E-07	0.02	8.61E-01	0.00	1.00E+00

FBgn0035122	mRpL17	461	0.68	2.30E-05	0.04	6.74E-01	0.00	1.00E+00
FBgn0036135	mRpL2	767	0.65	2.40E-05	0.01	9.37E-01	0.00	1.00E+00
FBgn0040907	mRpL33	284	1.11	1.42E-07	0.00	9.86E-01	-0.01	1.00E+00
FBgn0038923	mRpL35	302	0.61	9.42E-04	-0.01	9.37E-01	0.00	1.00E+00
FBgn0030552	mRpL38	817	0.65	1.45E-05	0.02	8.73E-01	0.00	1.00E+00
FBgn0037330	mRpL44	725	0.66	2.81E-05	0.02	8.52E-01	0.00	1.00E+00
FBgn0031357	mRpL48	312	0.58	5.52E-03	0.01	9.18E-01	0.00	1.00E+00
FBgn0030433	mRpL49	450	0.84	2.67E-05	0.03	8.00E-01	0.00	1.00E+00
FBgn0028648	mRpL50	407	0.69	2.41E-04	0.01	9.24E-01	0.00	1.00E+00
FBgn0032053	mRpL51	425	0.96	1.82E-08	0.01	9.68E-01	-0.01	1.00E+00
FBgn0033208	mRpL52	367	0.95	2.88E-07	0.03	7.47E-01	0.00	1.00E+00
FBgn0038678	mRpL55	253	0.72	4.36E-04	0.00	9.72E-01	0.00	1.00E+00
FBgn0044030	mRpS14	193	1.02	3.28E-05	0.07	4.31E-01	0.00	1.00E+00
FBgn0030572	mRpS25	392	0.59	8.81E-04	0.03	7.83E-01	0.00	1.00E+00
FBgn0044510	mRpS5	493	-0.93	9.94E-07	-0.01	9.26E-01	0.00	1.00E+00
FBgn0011666	msi	2622	-0.62	3.63E-04	0.01	9.71E-01	0.00	1.00E+00
FBgn0027948	msps	6613	-0.62	3.50E-06	-0.01	9.70E-01	0.00	1.00E+00
FBgn0013672	mt:ATPase6	7880	-3.31	5.37E-41	-0.02	8.94E-01	0.00	1.00E+00
FBgn0013673	mt:ATPase8	1600	-3.84	3.78E-230	0.02	9.06E-01	0.02	9.34E-01
FBgn0013674	mt:CoI	59337	-0.61	1.51E-02	0.01	8.92E-01	0.00	1.00E+00
FBgn0013675	mt:CoII	18791	-1.47	1.12E-09	0.00	9.75E-01	-0.01	1.00E+00
FBgn0013686	mt:lrRNA	420131	-2.84	3.45E-10	0.02	8.37E-01	0.00	1.00E+00
FBgn0013679	mt:ND1	4891	-3.62	7.13E-14	0.02	8.91E-01	0.00	1.00E+00
FBgn0013680	mt:ND2	1185	-4.38	3.24E-10	-0.03	7.42E-01	0.00	1.00E+00
FBgn0013681	mt:ND3	583	-3.85	7.22E-11	0.02	8.24E-01	0.01	1.00E+00
FBgn0262952	mt:ND4	3538	-2.38	7.67E-11	0.04	7.12E-01	0.00	1.00E+00
FBgn0013684	mt:ND5	6582	-2.51	1.53E-58	0.00	9.88E-01	0.03	4.80E-01
FBgn0013685	mt:ND6	440	-0.91	5.02E-03	-0.02	8.68E-01	0.00	1.00E+00
FBgn0013688	mt:srRNA	534	-0.37	6.27E-02	1.14	2.01E-02	0.00	1.00E+00
FBgn0013698	mt:tRNA:Leu-TAG	25	-2.56	3.95E-05	0.02	9.10E-01	0.00	1.00E+00
FBgn0013710	mt:tRNA:Tyr-GTA	24	-0.61	2.14E-02	-0.01	9.71E-01	0.00	1.00E+00
FBgn0028956	mthl3	3240	-0.66	8.47E-16	0.05	5.44E-01	0.00	1.00E+00
FBgn0034219	mthl4	587	-0.91	1.26E-09	-0.02	8.83E-01	0.00	1.00E+00
FBgn0052475	mthl8	493	-0.52	7.63E-04	-0.06	6.13E-01	10.83	1.59E-36
FBgn0025352	Mtpbeta	4531	0.62	3.72E-12	-0.09	1.74E-01	0.00	1.00E+00
FBgn0262737	mub	5736	-0.69	5.78E-07	-0.05	5.79E-01	0.00	1.00E+00
FBgn0002887	mus201	555	-0.84	2.67E-06	0.57	8.19E-05	0.00	1.00E+00
FBgn0264272	mwh	95	-0.49	2.83E-02	-1.28	1.73E-08	0.00	1.00E+00
FBgn0262656	Myc	3026	-1.25	4.29E-08	0.01	9.30E-01	0.00	1.00E+00
FBgn0026199	myo	1852	-1.04	1.52E-11	0.02	8.64E-01	0.00	1.00E+00
FBgn0039157	Myo95E	879	-0.68	4.80E-07	0.00	9.96E-01	0.00	1.00E+00
FBgn0051216	Naam	156	-0.21	3.64E-01	0.62	4.56E-04	0.01	1.00E+00
FBgn0086904	Nacalpha	17191	0.66	9.52E-13	-0.05	5.39E-01	0.00	1.00E+00
FBgn0260795	NaPi-III	1252	-0.59	1.45E-05	-0.07	3.45E-01	-0.04	4.03E-01
FBgn0085417	natalisin	246	1.08	1.55E-09	0.12	1.55E-01	0.02	6.27E-01
FBgn0013303	Nca	871	-0.82	8.10E-09	-0.03	8.10E-01	0.00	1.00E+00
FBgn0086707	ncm	2162	-1.09	8.23E-10	0.03	7.48E-01	0.00	1.00E+00
FBgn0031021	ND-18	707	0.84	1.01E-05	0.08	3.70E-01	0.00	1.00E+00
FBgn0266582	ND-30	1362	0.67	1.79E-09	0.04	6.37E-01	0.00	1.00E+00
FBgn0037001	ND-39	2350	0.93	2.39E-21	0.03	7.96E-01	-0.01	1.00E+00
FBgn0058002	ND-AGGG	176	-1.41	4.06E-09	0.04	6.30E-01	0.00	1.00E+00
FBgn0034645	ND-B12	751	0.88	5.97E-07	-0.06	4.68E-01	0.00	1.00E+00
FBgn0025839	ND-B14.5A	357	0.75	3.67E-05	0.06	5.10E-01	0.00	1.00E+00
FBgn0034576	ND-B14.7	900	0.81	1.14E-06	0.04	6.74E-01	0.00	1.00E+00
FBgn0033961	ND-B15	514	0.94	2.40E-06	0.03	7.82E-01	0.00	1.00E+00
FBgn0029868	ND-B16.6	1491	0.95	1.26E-13	0.02	9.07E-01	0.00	1.00E+00
FBgn0001989	ND-B17	829	0.73	7.01E-07	-0.01	9.18E-01	0.00	1.00E+00
FBgn0031436	ND-B17.2	939	1.38	2.39E-21	-0.01	9.33E-01	0.00	1.00E+00
FBgn0030605	ND-B18	697	0.72	1.67E-08	0.07	4.19E-01	0.00	1.00E+00
FBgn0085468	ND-MWFE	607	0.80	3.40E-05	0.00	9.82E-01	0.00	1.00E+00
FBgn0021967	ND-PDSW	1240	1.05	1.19E-06	0.10	2.58E-01	0.00	1.00E+00
FBgn0017430	Nelf-E	566	0.19	2.49E-01	0.51	4.14E-07	0.90	5.05E-21
FBgn0027570	Nep2	1864	0.24	1.37E-01	-1.19	2.14E-22	-0.72	4.22E-08
FBgn0039564	Nep7	118	0.99	2.44E-04	0.02	8.45E-01	0.10	2.75E-01
FBgn0032393	Nfs1	892	0.16	3.80E-01	1.00	1.30E-19	0.00	1.00E+00
FBgn0036101	NijA	1536	-0.22	1.07E-01	-0.38	2.26E-04	-0.67	1.96E-10
FBgn0038079	NijC	67	0.83	2.32E-03	0.01	9.16E-01	-0.01	1.00E+00
FBgn0259896	NimC1	57	0.18	4.82E-01	-2.27	4.02E-06	-0.01	1.00E+00
FBgn0026401	Nipped-B	5024	-1.01	7.71E-10	0.01	9.69E-01	0.00	1.00E+00
FBgn0024321	NK7.1	331	-0.67	2.02E-04	-0.02	8.22E-01	0.03	5.71E-01
FBgn0051547	NKCC	84	0.03	9.36E-01	1.28	1.77E-03	-0.01	1.00E+00
FBgn0031866	Nlg2	15	2.40	3.35E-04	-0.03	7.75E-01	0.00	1.00E+00
FBgn0011817	nmo	3519	-0.72	9.06E-07	0.04	6.54E-01	0.00	1.00E+00
FBgn0037617	nom	365	-0.63	4.56E-04	0.05	6.11E-01	0.01	1.00E+00
FBgn0015520	nonA-l	647	-0.65	1.76E-06	-0.11	1.51E-01	0.00	1.00E+00
FBgn0033029	Not3	5518	-0.69	2.88E-13	-0.05	5.72E-01	0.00	1.00E+00
FBgn0027785	NP15.6	1071	1.36	1.53E-13	0.02	8.85E-01	0.00	1.00E+00
FBgn0031381	Npc2a	201	0.59	1.30E-02	-0.01	9.10E-01	0.00	1.00E+00
FBgn0035092	Nplp1	42	-0.16	5.61E-01	-1.43	6.65E-05	0.00	1.00E+00

FBgn0040717	Nplp4	686	1.01	6.07E-04	-0.62	1.63E-02	0.96	4.01E-03
FBgn0261526	NT1	183	-0.07	8.23E-01	-0.80	1.26E-03	0.00	1.00E+00
FBgn0028411	Nxt1	883	0.59	7.17E-04	0.13	1.56E-01	0.00	1.00E+00
FBgn0027791	O-fut2	218	0.96	2.28E-05	0.00	9.76E-01	0.00	1.00E+00
FBgn0022774	Oat	220	-0.06	8.35E-01	-0.69	2.98E-02	-0.01	1.00E+00
FBgn0038344	obe	1568	-0.17	2.99E-01	0.87	8.91E-14	0.01	1.00E+00
FBgn0046875	Obp83g	76	1.39	1.93E-05	-0.07	4.76E-01	0.00	1.00E+00
FBgn0039678	Obp99a	345	2.49	2.05E-15	-2.20	1.17E-27	0.00	1.00E+00
FBgn0027600	obst-B	1761	0.83	9.71E-08	-0.04	6.25E-01	0.00	1.00E+00
FBgn0028996	onecut	28	-0.17	4.64E-01	2.85	7.82E-12	0.04	6.14E-01
FBgn0026393	Or43b	242	-0.70	3.46E-05	0.04	7.13E-01	0.00	1.00E+00
FBgn0039551	Or98a	41	-0.03	8.31E-01	8.76	7.97E-13	0.00	1.00E+00
FBgn0040279	Osi14	132	0.95	6.82E-04	1.56	3.99E-09	0.18	1.42E-01
FBgn0032197	ova	1116	-0.77	3.32E-07	0.00	9.86E-01	0.00	1.00E+00
FBgn0011227	ox	799	2.14	2.56E-51	0.02	8.38E-01	0.00	1.00E+00
FBgn0260799	p120ctn	3379	-1.11	1.81E-16	0.00	9.86E-01	0.00	1.00E+00
FBgn0265297	pAbp	44968	-0.66	4.08E-04	0.02	8.24E-01	0.00	1.00E+00
FBgn0005648	Pabp2	4791	-0.80	3.13E-09	0.15	7.73E-02	0.00	1.00E+00
FBgn0060296	pain	1156	-0.10	5.67E-01	-0.63	8.12E-08	-0.03	4.82E-01
FBgn0038100	Paip2	1311	-0.80	1.58E-07	0.00	1.00E+00	0.00	1.00E+00
FBgn0085432	pan	2131	-0.59	6.42E-03	0.00	9.77E-01	0.00	1.00E+00
FBgn0035397	PAN3	3490	-0.71	4.02E-11	-0.10	1.74E-01	0.00	1.00E+00
FBgn0010247	Parp	2656	-0.94	3.12E-08	0.38	6.00E-03	0.00	1.00E+00
FBgn0036007	path	4128	0.03	8.74E-01	0.03	8.10E-01	0.80	5.05E-13
FBgn0027580	PCB	2192	0.26	2.54E-01	-0.84	2.06E-04	-0.02	7.92E-01
FBgn0024841	Pcd	661	0.96	6.82E-08	-0.02	8.22E-01	0.00	1.00E+00
FBgn0086768	Pcmt	1557	0.98	5.51E-10	0.02	8.59E-01	0.00	1.00E+00
FBgn0005655	PCNA	4886	0.69	2.73E-15	0.06	3.90E-01	0.00	1.00E+00
FBgn0036580	PDCD-5	930	0.91	5.47E-08	0.04	6.66E-01	0.00	1.00E+00
FBgn0039635	Pdhb	1804	0.61	4.78E-04	0.03	7.61E-01	0.00	1.00E+00
FBgn0016694	Pdp1	373	-1.14	5.67E-06	0.00	9.98E-01	0.00	1.00E+00
FBgn0031969	pes	393	-0.80	8.68E-06	0.00	9.86E-01	0.00	1.00E+00
FBgn0031530	Pgant2	288	0.65	9.45E-04	0.04	6.43E-01	0.00	1.00E+00
FBgn0031681	Pgant5	2854	-0.59	8.73E-06	-0.05	5.36E-01	0.00	1.00E+00
FBgn0014869	Pglym78	1405	0.87	6.68E-09	0.25	1.76E-02	0.00	1.00E+00
FBgn0035976	PGRP-LC	112	0.10	7.03E-01	-0.74	1.85E-04	-1.41	7.16E-13
FBgn0035438	PHGPx	2555	0.73	1.23E-05	0.04	7.06E-01	0.00	1.00E+00
FBgn0035089	Phk-3	2712	0.81	2.59E-09	-0.04	6.45E-01	-0.01	1.00E+00
FBgn0002521	pho	2284	-1.27	2.01E-21	-0.06	5.03E-01	0.00	1.00E+00
FBgn0036522	Phs	2259	-0.66	1.14E-07	0.03	7.54E-01	0.00	1.00E+00
FBgn0033479	PIG-N	188	-0.69	2.91E-04	-0.08	3.40E-01	0.00	1.00E+00
FBgn0086448	PIG-Q	352	-0.64	5.87E-05	-0.10	2.17E-01	-0.01	1.00E+00
FBgn0038966	pinta	68	-0.07	8.23E-01	2.59	5.85E-18	1.24	2.00E-04
FBgn0039924	PIP4K	2088	-0.89	2.33E-15	-0.03	8.07E-01	-0.01	1.00E+00
FBgn0030400	Pits	2206	-0.74	4.02E-07	0.01	9.52E-01	0.00	1.00E+00
FBgn0004872	piwi	37	-0.40	1.07E-01	-0.69	2.81E-02	0.00	1.00E+00
FBgn0022382	Pka-R2	572	-0.62	4.35E-03	0.01	9.63E-01	0.00	1.00E+00
FBgn0036192	Pldn	144	-0.60	3.59E-03	-0.02	8.38E-01	0.00	1.00E+00
FBgn0259214	PMCA	7693	-0.73	5.33E-08	-0.01	9.33E-01	0.00	1.00E+00
FBgn0037737	Pnn	621	-0.66	3.63E-06	0.00	9.86E-01	0.00	1.00E+00
FBgn0036696	Pop5	239	0.63	2.04E-03	0.17	9.54E-02	0.01	1.00E+00
FBgn0003130	Poxn	231	0.63	5.12E-04	0.64	4.29E-06	0.05	3.88E-01
FBgn0053508	ppk13	1275	-0.27	1.10E-01	1.86	1.49E-64	0.45	1.10E-03
FBgn0261363	PPO3	59	0.63	1.08E-02	0.03	7.94E-01	0.00	1.00E+00
FBgn0011474	PR-Set7	2050	-0.63	3.34E-05	0.01	9.28E-01	0.00	1.00E+00
FBgn0033635	Prip	107	-0.08	7.73E-01	-0.62	4.40E-03	-0.06	3.60E-01
FBgn0086134	Prosalpha2	4106	0.70	1.72E-16	-0.03	7.26E-01	-0.03	5.00E-01
FBgn0261394	Prosalpha3	3487	0.71	2.64E-08	-0.03	7.85E-01	0.00	1.00E+00
FBgn0027587	Prp4k	992	-0.65	1.36E-11	0.03	7.55E-01	0.16	1.67E-02
FBgn0033518	Prx2540-2	56	0.53	4.22E-02	-0.62	1.93E-02	0.00	1.00E+00
FBgn0035770	pst	722	0.88	2.24E-04	0.18	1.36E-01	0.00	1.00E+00
FBgn0026379	Pten	1333	-0.83	2.36E-09	-0.05	5.30E-01	0.00	1.00E+00
FBgn0034085	Ptp52F	12	-0.16	4.59E-01	-0.29	1.17E-01	-0.60	3.13E-02
FBgn0014007	Ptp69D	2086	-0.89	2.12E-13	-0.14	6.28E-02	0.00	1.00E+00
FBgn0243512	puc	688	-0.86	6.08E-06	0.00	9.80E-01	-0.01	1.00E+00
FBgn0033226	puml	453	0.27	1.11E-01	0.65	3.52E-09	0.01	1.00E+00
FBgn0022361	Pur-alpha	1438	-1.00	6.23E-08	0.01	9.30E-01	0.00	1.00E+00
FBgn0004577	Pxd	86	-0.01	9.75E-01	-0.87	3.05E-02	-0.03	6.72E-01
FBgn0033649	pyr	313	-0.85	3.77E-07	-0.05	5.43E-01	0.00	1.00E+00
FBgn0052412	QC	164	0.44	5.93E-02	-0.01	9.12E-01	-1.14	2.11E-06
FBgn0022987	qkr54B	1628	-1.10	1.29E-12	-0.05	5.42E-01	0.00	1.00E+00
FBgn0022986	qkr58E-1	1684	-0.95	7.55E-09	-0.02	8.81E-01	0.00	1.00E+00
FBgn0022984	qkr58E-3	1887	-0.97	6.58E-09	0.06	4.61E-01	0.00	1.00E+00
FBgn0051864	Qtzl	82	-0.35	1.62E-01	1.34	6.16E-08	-0.01	9.19E-01
FBgn0014010	Rab5	3111	-0.75	1.02E-15	0.00	9.99E-01	-0.01	1.00E+00
FBgn0030200	RabX2	20	0.03	8.57E-01	6.65	2.74E-11	0.00	1.00E+00
FBgn0014011	Rac2	1610	-0.87	1.83E-12	-0.04	6.69E-01	-0.01	1.00E+00
FBgn0020618	Rack1	56381	1.07	6.74E-36	-0.32	1.48E-05	0.00	1.00E+00
FBgn0034728	rad50	1403	-0.96	1.79E-10	0.87	1.40E-13	0.01	9.73E-01

FBgn0034646	Rae1	1214	0.61	3.94E-06	0.06	4.85E-01	0.00	1.00E+00
FBgn0003079	Raf	1265	-0.81	2.61E-16	-0.03	7.61E-01	0.00	1.00E+00
FBgn0039110	RanBP3	2099	0.62	1.98E-08	-0.02	8.88E-01	-0.01	9.85E-01
FBgn0004636	Rap1	4020	-1.04	1.23E-12	-0.01	9.55E-01	0.00	1.00E+00
FBgn0031745	rau	255	0.61	1.41E-03	-0.05	5.50E-01	0.00	1.00E+00
FBgn0030479	Rbp1-like	2083	-1.39	8.00E-35	0.06	4.91E-01	0.01	1.00E+00
FBgn0031047	Rcd-1	3879	0.62	4.92E-07	-0.02	8.48E-01	0.00	1.00E+00
FBgn0039644	rdog	430	-0.19	4.02E-01	2.41	3.73E-52	0.00	1.00E+00
FBgn0264493	rdx	1794	-0.80	1.57E-15	0.00	9.77E-01	0.01	1.00E+00
FBgn0027375	RecQ5	730	-1.02	3.31E-11	0.00	1.00E+00	0.01	1.00E+00
FBgn0021800	Reph	522	-0.83	9.85E-06	0.07	4.30E-01	0.01	9.70E-01
FBgn0021906	RFeSP	1542	0.74	6.01E-07	0.06	5.05E-01	-0.01	1.00E+00
FBgn0014020	Rho1	8527	-0.78	9.28E-22	0.03	7.71E-01	0.00	1.00E+00
FBgn0265605	Ric	315	-0.61	4.46E-04	-0.02	8.59E-01	0.00	1.00E+00
FBgn0003256	rl	1022	-0.65	3.22E-05	-0.01	9.40E-01	0.00	1.00E+00
FBgn0014022	Rlb1	390	-0.84	1.02E-05	0.04	6.98E-01	0.00	1.00E+00
FBgn0262116	RNASEK	1235	-0.70	2.47E-07	0.09	2.58E-01	-0.03	5.42E-01
FBgn0065098	RNaseMRP:RNA	80	4.59	4.42E-10	0.57	3.60E-02	-0.03	6.87E-01
FBgn0046696	RNaseP:RNA	131	2.12	4.96E-12	0.05	5.84E-01	0.00	1.00E+00
FBgn0250838	roh	1748	0.64	8.88E-05	-0.03	7.61E-01	0.00	1.00E+00
FBgn0005649	Rox8	5628	-0.89	7.39E-27	-0.13	3.05E-02	0.00	1.00E+00
FBgn0010173	RpA-70	3830	-0.17	1.04E-01	0.58	8.68E-17	0.00	1.00E+00
FBgn0262954	Rpb12	562	1.01	4.13E-07	0.06	4.95E-01	-0.01	9.40E-01
FBgn0050499	Rpe	1477	0.82	4.26E-11	-0.02	8.91E-01	0.00	1.00E+00
FBgn0004855	RpII15	1036	0.63	2.77E-06	-0.01	9.39E-01	-0.01	1.00E+00
FBgn0026373	RpII33	900	0.63	3.15E-05	0.02	8.21E-01	0.00	1.00E+00
FBgn0022981	rpk	458	0.06	7.97E-01	0.75	1.76E-08	-0.01	1.00E+00
FBgn0036213	RpL10Ab	42243	0.74	3.62E-14	-0.35	3.27E-05	0.00	1.00E+00
FBgn0013325	RpL11	28172	0.92	3.13E-18	-0.02	8.26E-01	0.00	1.00E+00
FBgn0034968	RpL12	29887	0.59	9.92E-06	-0.13	1.05E-01	0.00	1.00E+00
FBgn0011272	RpL13	31038	1.32	7.08E-19	0.01	9.16E-01	0.00	1.00E+00
FBgn0037351	RpL13A	29649	1.58	4.20E-41	-0.03	7.96E-01	0.00	1.00E+00
FBgn0017579	RpL14	24860	1.54	1.76E-29	-0.04	7.13E-01	0.00	1.00E+00
FBgn0029897	RpL17	24696	1.51	2.72E-26	-0.03	7.43E-01	0.00	1.00E+00
FBgn0035753	RpL18	25719	1.26	6.13E-19	-0.04	7.01E-01	0.00	1.00E+00
FBgn0010409	RpL18A	33698	1.19	5.09E-22	0.01	9.46E-01	0.00	1.00E+00
FBgn0285950	RpL19	37514	0.70	2.79E-10	-0.02	8.39E-01	0.00	1.00E+00
FBgn0034837	RpL22-like	14	0.31	1.65E-01	-2.96	6.20E-04	0.00	1.00E+00
FBgn0010078	RpL23	29740	1.18	6.09E-15	-0.01	9.33E-01	0.00	1.00E+00
FBgn0032518	RpL24	20688	1.16	1.40E-10	0.00	9.94E-01	0.00	1.00E+00
FBgn0036825	RpL26	26262	1.18	5.38E-19	-0.09	2.57E-01	0.00	1.00E+00
FBgn0039359	RpL27	30893	1.82	7.59E-44	-0.07	4.00E-01	0.00	1.00E+00
FBgn0035422	RpL28	33296	0.92	3.35E-12	-0.05	5.91E-01	0.00	1.00E+00
FBgn0016726	RpL29	15027	2.47	1.56E-58	-0.03	7.67E-01	0.00	1.00E+00
FBgn0020910	RpL3	84241	0.82	9.57E-18	-0.32	1.18E-04	0.00	1.00E+00
FBgn0086710	RpL30	15925	0.82	5.69E-08	-0.15	9.68E-02	0.00	1.00E+00
FBgn0285949	RpL31	23945	1.87	4.08E-31	-0.07	3.96E-01	0.00	1.00E+00
FBgn0002626	RpL32	26148	1.55	3.34E-30	-0.08	3.38E-01	0.00	1.00E+00
FBgn0039406	RpL34a	4745	0.79	2.78E-08	0.00	9.93E-01	0.00	1.00E+00
FBgn0037686	RpL34b	14511	1.00	8.70E-10	0.00	1.00E+00	0.00	1.00E+00
FBgn0029785	RpL35	22897	1.11	1.57E-14	-0.04	6.97E-01	0.00	1.00E+00
FBgn0037328	RpL35A	18082	1.07	1.66E-06	0.03	7.56E-01	0.00	1.00E+00
FBgn0002579	RpL36	22750	0.63	4.03E-05	-0.05	5.93E-01	0.00	1.00E+00
FBgn0031980	RpL36A	20310	1.45	5.37E-17	-0.01	9.36E-01	0.00	1.00E+00
FBgn0030616	RpL37a	28870	1.44	2.13E-31	-0.01	9.31E-01	0.00	1.00E+00
FBgn0261608	RpL37A	15974	1.17	6.51E-15	-0.05	5.40E-01	0.00	1.00E+00
FBgn0040007	RpL38	10930	-1.22	8.25E-12	0.01	9.45E-01	0.00	1.00E+00
FBgn0023170	RpL39	14842	1.09	6.07E-13	-0.01	9.54E-01	0.00	1.00E+00
FBgn0003279	RpL4	68623	0.74	7.97E-10	-0.01	9.31E-01	0.00	1.00E+00
FBgn0003941	RpL40	28783	1.13	3.13E-23	-0.04	6.70E-01	0.00	1.00E+00
FBgn0066084	RpL41	44221	1.27	1.23E-12	-0.10	2.39E-01	0.00	1.00E+00
FBgn0039857	RpL6	40858	0.66	5.68E-09	-0.02	8.96E-01	0.00	1.00E+00
FBgn0005593	RpL7	46015	0.88	6.36E-19	-0.07	3.97E-01	0.00	1.00E+00
FBgn0014026	RpL7A	51685	0.64	1.91E-08	-0.04	6.85E-01	0.00	1.00E+00
FBgn0261602	RpL8	41973	0.93	2.53E-13	-0.03	7.65E-01	0.00	1.00E+00
FBgn0015756	RpL9	33572	1.23	3.91E-24	-0.04	6.30E-01	0.00	1.00E+00
FBgn0000100	RpLP0	49121	0.88	2.69E-10	-0.02	8.64E-01	0.00	1.00E+00
FBgn0002593	RpLP1	34330	1.50	2.88E-29	-0.05	6.18E-01	0.00	1.00E+00
FBgn0003274	RpLP2	29029	0.72	3.01E-04	0.02	8.90E-01	0.00	1.00E+00
FBgn0015283	Rpn10	3237	0.60	8.41E-07	-0.04	6.60E-01	-0.01	8.51E-01
FBgn0285947	RpS10b	22456	0.90	7.94E-17	-0.04	7.10E-01	0.00	1.00E+00
FBgn0033699	RpS11	27054	1.05	7.21E-11	-0.02	8.37E-01	0.00	1.00E+00
FBgn0286213	RpS12	22585	1.49	7.12E-26	-0.27	1.12E-02	0.00	1.00E+00
FBgn0010265	RpS13	36129	0.85	1.52E-13	-0.04	7.11E-01	0.00	1.00E+00
FBgn0004403	RpS14a	14676	0.84	1.97E-10	-0.02	8.88E-01	0.00	1.00E+00
FBgn0004404	RpS14b	5724	1.39	5.22E-15	0.00	9.94E-01	0.00	1.00E+00
FBgn0034138	RpS15	32466	1.00	1.50E-15	-0.12	1.11E-01	0.00	1.00E+00
FBgn0010198	RpS15Aa	22650	0.73	3.20E-09	-0.08	3.36E-01	0.00	1.00E+00
FBgn0033555	RpS15Ab	2011	0.81	1.69E-12	-0.06	5.17E-01	0.00	1.00E+00

FBgn0005533	RpS17	26903	1.72	4.39E-37	-0.06	4.59E-01	0.00	1.00E+00
FBgn0010411	RpS18	32633	1.38	2.01E-26	-0.05	5.78E-01	0.00	1.00E+00
FBgn0010412	RpS19a	27379	1.20	1.49E-08	0.00	9.88E-01	0.00	1.00E+00
FBgn0004867	RpS2	48438	0.78	4.36E-11	-0.12	1.17E-01	0.00	1.00E+00
FBgn0019936	RpS20	22643	1.07	7.12E-14	-0.18	4.75E-02	0.00	1.00E+00
FBgn0015521	RpS21	11086	0.72	1.53E-06	-0.03	8.07E-01	0.00	1.00E+00
FBgn0033912	RpS23	31632	1.84	3.62E-35	-0.04	6.97E-01	0.00	1.00E+00
FBgn0261596	RpS24	32050	1.17	2.61E-22	-0.08	2.62E-01	-0.01	1.00E+00
FBgn0086472	RpS25	28124	0.59	1.91E-05	-0.04	6.82E-01	0.00	1.00E+00
FBgn0261597	RpS26	31285	1.52	3.40E-35	-0.02	8.47E-01	0.00	1.00E+00
FBgn0030136	RpS28b	20297	0.86	1.46E-06	-0.07	3.87E-01	0.00	1.00E+00
FBgn0261599	RpS29	31258	0.83	1.80E-11	-0.07	4.10E-01	0.00	1.00E+00
FBgn0002622	RpS3	41096	1.00	7.80E-28	-0.10	1.22E-01	0.00	1.00E+00
FBgn0038834	RpS30	22722	0.64	4.12E-07	-0.04	6.49E-01	0.00	1.00E+00
FBgn0011284	RpS4	55820	1.07	4.18E-29	-0.06	4.68E-01	0.00	1.00E+00
FBgn0002590	RpS5a	48391	0.92	1.61E-23	-0.07	3.37E-01	0.00	1.00E+00
FBgn0261592	RpS6	46007	1.20	1.79E-20	-0.04	6.36E-01	0.00	1.00E+00
FBgn0039757	RpS7	40640	0.89	5.35E-11	-0.05	5.82E-01	0.00	1.00E+00
FBgn0039713	RpS8	39353	1.28	3.54E-25	-0.04	6.64E-01	0.00	1.00E+00
FBgn0010408	RpS9	31452	0.59	9.82E-06	-0.15	7.18E-02	0.00	1.00E+00
FBgn0028686	Rpt3	3750	0.69	2.73E-15	-0.02	8.50E-01	-0.01	9.19E-01
FBgn0283472	S6k	1848	-1.11	1.68E-13	0.06	4.86E-01	0.02	7.50E-01
FBgn0262866	S6kII	523	-0.60	8.22E-05	0.05	5.74E-01	0.00	1.00E+00
FBgn0037672	sage	118	-0.97	7.66E-04	0.02	8.54E-01	0.00	1.00E+00
FBgn0026371	SAK	1348	-0.58	1.68E-07	-0.05	6.10E-01	0.01	1.00E+00
FBgn0013334	Sap47	1082	-0.65	1.87E-05	0.00	9.73E-01	0.00	1.00E+00
FBgn0267378	sau	2766	-0.71	2.10E-12	-0.08	2.97E-01	0.00	1.00E+00
FBgn0051950	Sbat	156	1.17	4.34E-07	0.07	4.00E-01	-0.01	1.00E+00
FBgn0261872	scaf6	2332	-0.63	8.81E-18	-0.03	7.70E-01	0.00	1.00E+00
FBgn0038038	Sccpdh2	466	1.03	4.86E-08	-0.02	8.24E-01	0.08	2.30E-01
FBgn0020907	Scp2	14	0.05	7.27E-01	0.07	5.58E-01	0.78	4.12E-02
FBgn0021765	scu	3185	1.00	5.91E-14	-0.11	1.65E-01	0.00	1.00E+00
FBgn0041094	scyl	8561	-1.33	8.68E-18	-0.06	4.87E-01	-0.01	1.00E+00
FBgn0003345	sd	7654	-0.68	6.92E-06	-0.01	9.09E-01	0.00	1.00E+00
FBgn0010415	Sdc	5389	-0.69	8.04E-06	-0.01	9.30E-01	0.00	1.00E+00
FBgn0014028	SdhB	1621	0.63	1.89E-06	0.00	9.76E-01	0.00	1.00E+00
FBgn0039112	SdhD	864	0.75	4.35E-07	-0.02	8.28E-01	0.00	1.00E+00
FBgn0053497	Sdic2	15	-0.38	1.03E-01	0.06	5.87E-01	2.96	5.80E-04
FBgn0035771	Sec63	3488	0.58	7.21E-13	0.02	8.44E-01	0.00	1.00E+00
FBgn0010414	SerT	71	-0.42	8.67E-02	-1.55	5.72E-07	-0.03	6.87E-01
FBgn0040022	Set1	2236	-1.19	1.64E-18	-0.04	6.94E-01	0.00	1.00E+00
FBgn0003371	sgg	3372	-0.85	5.82E-06	0.07	4.58E-01	0.00	1.00E+00
FBgn0052423	shep	1920	-0.90	1.74E-10	0.04	6.70E-01	0.00	1.00E+00
FBgn0003392	shi	2787	-0.60	3.04E-06	-0.03	7.56E-01	0.00	1.00E+00
FBgn0263873	sick	3454	0.08	6.69E-01	3.08	4.79E-168	0.00	1.00E+00
FBgn0010762	simj	2866	-0.61	1.03E-05	-0.07	4.22E-01	0.00	1.00E+00
FBgn0037802	Sirt6	173	-0.63	1.58E-03	0.03	7.51E-01	0.00	1.00E+00
FBgn0031971	Sirup	481	0.45	4.83E-02	-0.90	1.93E-04	0.00	1.00E+00
FBgn0031998	SLC5A11	132	-0.38	1.05E-01	-0.99	6.52E-04	-0.01	9.70E-01
FBgn0037810	sle	3373	-0.67	2.17E-05	0.02	8.42E-01	0.00	1.00E+00
FBgn0037203	slif	87	-0.66	1.01E-02	-0.06	5.03E-01	0.00	1.00E+00
FBgn0040011	Slmap	2039	-0.78	5.55E-15	0.04	7.15E-01	0.00	1.00E+00
FBgn0040283	SMC1	3037	-0.64	1.85E-06	-0.02	8.19E-01	0.00	1.00E+00
FBgn0261789	SmD2	1956	0.73	2.12E-06	0.04	6.65E-01	0.00	1.00E+00
FBgn0000426	SmF	1111	0.73	3.23E-05	0.01	9.46E-01	0.00	1.00E+00
FBgn0003444	smo	2301	-0.63	3.91E-07	-0.10	2.03E-01	0.00	1.00E+00
FBgn0036282	Smyd4-2	19	1.33	8.38E-04	1.15	6.49E-03	0.00	1.00E+00
FBgn0086129	snama	1490	-0.65	8.22E-09	0.02	8.84E-01	0.01	1.00E+00
FBgn0011288	Snap25	98	-0.08	7.87E-01	4.02	5.30E-61	-0.02	8.64E-01
FBgn0030026	sni	488	0.71	2.21E-08	0.07	3.85E-01	0.00	1.00E+00
FBgn0083027	snoRNA:Psi18S-531	228	0.76	7.84E-06	0.01	9.54E-01	-0.99	6.07E-15
FBgn0065099	snRNA:7SK	74	0.77	8.34E-03	0.12	3.03E-01	-0.01	1.00E+00
FBgn0041721	snRNA:U12	72	0.99	6.65E-04	0.25	9.48E-02	0.02	9.19E-01
FBgn0052758	Snx27	1056	-0.64	4.13E-06	0.00	9.90E-01	0.00	1.00E+00
FBgn0003462	Sod1	4607	0.82	6.89E-09	-0.01	9.62E-01	-0.02	7.72E-01
FBgn0033631	Sod3	3675	0.63	1.89E-07	-0.08	3.00E-01	-0.01	1.00E+00
FBgn0042630	Sox21b	15	0.01	9.47E-01	-0.84	2.59E-02	0.01	1.00E+00
FBgn0020378	Sp1	380	0.20	4.39E-01	-2.11	5.44E-08	0.01	1.00E+00
FBgn0035710	SP1173	2433	0.10	5.98E-01	0.11	1.53E-01	0.66	3.68E-07
FBgn0026562	SPARC	1433	0.81	2.04E-06	-0.34	1.04E-02	0.00	1.00E+00
FBgn0040623	Spase12	1070	0.90	4.41E-07	0.02	8.81E-01	-0.01	1.00E+00
FBgn0039172	Spase22-23	2345	0.62	4.08E-07	0.01	9.32E-01	-0.01	1.00E+00
FBgn0037025	Spc105R	1970	-0.64	3.39E-06	0.00	9.82E-01	0.02	6.68E-01
FBgn0031549	Spindly	889	-0.77	1.08E-11	-0.58	2.41E-11	-0.47	5.60E-07
FBgn0003483	spn-E	262	0.05	8.37E-01	0.95	2.55E-08	0.01	1.00E+00
FBgn0039795	Spn100A	2226	0.21	4.08E-01	-0.60	2.38E-02	-0.01	1.00E+00
FBgn0028990	Spn27A	3624	0.72	4.88E-15	-0.12	5.13E-02	0.00	1.00E+00
FBgn0033115	Spn42De	140	1.10	3.10E-06	0.08	3.73E-01	0.00	1.00E+00
FBgn0024294	Spn43Aa	3847	0.81	1.21E-04	-0.01	9.37E-01	-0.02	8.73E-01

FBgn0033574	Spn47C	40	-2.01	4.50E-05	2.30	2.05E-07	0.00	1.00E+00
FBgn0003486	spo	14	-0.61	1.57E-02	0.01	9.14E-01	0.00	1.00E+00
FBgn0263987	spoon	4782	-0.94	1.55E-08	-0.01	9.13E-01	0.00	1.00E+00
FBgn0031260	Spp	2973	0.59	1.42E-06	-0.01	9.49E-01	0.00	1.00E+00
FBgn0032362	spz4	38	-1.26	9.87E-05	-0.02	8.49E-01	0.00	1.00E+00
FBgn0263396	sqd	24176	-1.21	2.03E-15	0.01	9.25E-01	0.00	1.00E+00
FBgn0037248	srl	2653	-0.64	2.17E-06	-0.05	5.95E-01	0.00	1.00E+00
FBgn0011481	Ssdp	3514	-1.21	3.35E-12	-0.02	8.47E-01	0.00	1.00E+00
FBgn0037665	St2	912	0.77	6.07E-04	-0.05	5.42E-01	-0.09	2.64E-01
FBgn0265052	St3	62	0.34	1.70E-01	1.40	5.50E-08	1.21	3.44E-06
FBgn0003517	sta	62664	1.36	1.84E-26	-0.14	8.33E-02	0.00	1.00E+00
FBgn0086779	step	1533	-0.70	1.73E-06	0.05	5.62E-01	0.00	1.00E+00
FBgn0046692	Stlk	960	-0.69	1.41E-04	0.03	7.68E-01	0.00	1.00E+00
FBgn0020299	stumps	1216	-0.16	1.84E-01	-0.61	1.14E-13	-0.01	1.00E+00
FBgn0086708	stv	2256	0.16	5.24E-01	-1.48	4.91E-10	0.01	1.00E+00
FBgn0014388	sty	1582	-0.70	7.56E-06	0.04	6.60E-01	0.00	1.00E+00
FBgn0003567	su(Hw)	2513	-0.63	1.09E-08	-0.01	9.69E-01	0.00	1.00E+00
FBgn0014391	sun	1616	1.01	5.14E-09	0.01	9.64E-01	-0.01	1.00E+00
FBgn0028675	Sur	11	-0.09	6.53E-01	0.58	4.64E-02	0.00	1.00E+00
FBgn0261403	sxc	3212	-0.69	8.92E-08	-0.05	6.02E-01	0.00	1.00E+00
FBgn0038826	Syp	23681	-1.10	4.58E-08	-0.03	7.82E-01	0.00	1.00E+00
FBgn0028400	Syt4	264	7.49	1.96E-06	4.89	1.96E-08	6.07	2.24E-12
FBgn0028398	Taf10	549	0.66	1.19E-04	-0.01	9.18E-01	0.00	1.00E+00
FBgn0004406	tam	490	0.29	8.95E-02	0.72	1.51E-09	0.01	8.66E-01
FBgn0021795	Tapdelta	3819	0.58	3.60E-05	-0.02	8.76E-01	0.00	1.00E+00
FBgn0034451	TBCB	731	0.19	2.42E-01	0.68	1.75E-10	0.58	3.63E-07
FBgn0285892	tea	768	-0.63	4.20E-05	-0.03	7.57E-01	0.00	1.00E+00
FBgn0261953	TfAP-2	180	0.34	1.52E-01	-2.03	5.20E-06	0.01	1.00E+00
FBgn0013347	TfIIA-S	1676	0.94	1.18E-09	0.02	8.85E-01	0.00	1.00E+00
FBgn0026869	Thd1	3729	-0.71	8.71E-05	-0.03	7.76E-01	0.01	1.00E+00
FBgn0261560	Thor	335	1.02	1.07E-03	-0.09	4.00E-01	0.00	1.00E+00
FBgn0032988	Tif-IA	2210	-0.65	1.22E-11	-0.06	4.87E-01	0.00	1.00E+00
FBgn0027359	Tim8	995	0.89	3.09E-07	0.03	7.23E-01	0.00	1.00E+00
FBgn0025879	Timp	183	1.31	4.85E-08	0.00	9.93E-01	0.00	1.00E+00
FBgn0004841	TkR86C	16	2.44	5.62E-04	6.69	3.31E-07	6.67	9.56E-07
FBgn0003721	Tm1	10585	-0.87	8.91E-13	-0.01	9.30E-01	0.00	1.00E+00
FBgn0267796	Tmc	187	-1.09	6.16E-06	-0.01	9.30E-01	-0.01	9.67E-01
FBgn0026160	tna	4864	-0.86	1.63E-08	-0.01	9.53E-01	0.00	1.00E+00
FBgn0033357	Tom7	1314	1.19	2.26E-12	0.04	6.63E-01	0.00	1.00E+00
FBgn0037751	topi	17	0.16	2.73E-01	3.33	7.94E-09	0.01	1.00E+00
FBgn0086355	Tpi	3109	0.75	5.13E-06	0.06	4.99E-01	0.00	1.00E+00
FBgn0031692	TpnC25D	30	-0.49	4.92E-02	-0.68	4.79E-02	-0.05	5.00E-01
FBgn0086674	Tpst	715	-1.40	5.38E-11	0.03	7.58E-01	0.00	1.00E+00
FBgn0030748	Traf-like	115	-0.73	5.07E-03	-0.08	4.80E-01	0.00	1.00E+00
FBgn0026319	Traf4	481	0.24	1.13E-01	0.76	4.59E-17	0.00	1.00E+00
FBgn0261793	Trf2	2420	-0.99	3.98E-07	0.03	7.55E-01	0.00	1.00E+00
FBgn0013263	Trl	369	-1.26	3.45E-08	-0.01	9.05E-01	0.00	1.00E+00
FBgn0050343	Tsen15	167	-1.57	1.05E-14	0.02	8.94E-01	0.00	1.00E+00
FBgn0003866	tsh	5545	-0.66	4.17E-20	-0.16	2.69E-03	0.00	1.00E+00
FBgn0031850	Tsp	2665	0.69	2.98E-10	-0.34	3.00E-04	0.00	1.00E+00
FBgn0032943	Tsp39D	1560	-0.58	8.20E-05	0.01	9.37E-01	0.00	1.00E+00
FBgn0029507	Tsp42Ed	56	0.33	1.26E-01	1.57	7.02E-04	-0.02	8.30E-01
FBgn0033130	Tsp42Ei	87	0.57	2.08E-02	-0.61	1.22E-02	-0.02	7.84E-01
FBgn0043550	Tsp68C	24	0.92	4.23E-03	3.43	4.52E-12	0.04	5.83E-01
FBgn0027865	Tsp96F	1091	-0.73	1.13E-04	0.00	9.93E-01	0.00	1.00E+00
FBgn0032744	Ttc19	220	-0.65	9.39E-04	1.15	1.18E-14	0.01	1.00E+00
FBgn0051108	TTLL5	618	-0.89	5.37E-10	0.00	9.85E-01	0.00	1.00E+00
FBgn0052364	tut	126	-0.50	3.44E-02	-0.04	6.75E-01	-3.19	3.46E-25
FBgn0039434	TwdlM	16	0.92	5.02E-03	0.34	1.02E-01	2.59	7.18E-05
FBgn0029170	TwdlT	119	0.43	7.55E-02	-1.52	1.34E-06	-0.11	2.46E-01
FBgn0003900	twi	636	0.03	8.92E-01	-0.69	5.16E-11	-0.12	1.00E-01
FBgn0262801	twr	2970	0.60	6.91E-05	-0.03	8.10E-01	-0.01	1.00E+00
FBgn0034636	twz	761	0.58	1.40E-03	-0.01	9.54E-01	0.03	6.00E-01
FBgn0029996	UbcE2H	1978	-0.73	9.00E-09	0.15	6.58E-02	0.01	1.00E+00
FBgn0026076	UBL3	1245	-0.79	6.80E-08	-0.02	8.24E-01	0.00	1.00E+00
FBgn0003944	Ubx	347	-0.41	9.19E-02	-1.48	4.15E-08	0.00	1.00E+00
FBgn0262124	uex	1998	-0.87	4.48E-09	0.01	9.32E-01	0.00	1.00E+00
FBgn0040259	Ugt302C1	1204	-0.23	8.13E-02	0.70	2.85E-15	0.00	1.00E+00
FBgn0040251	Ugt302K1	820	-1.93	1.49E-26	2.29	4.84E-76	0.00	1.00E+00
FBgn0040091	Ugt317A1	411	0.43	6.49E-03	-1.04	4.52E-19	-1.12	4.52E-23
FBgn0045800	Uhg1	543	-0.62	8.92E-04	0.04	6.63E-01	0.00	1.00E+00
FBgn0083124	Uhg4	570	-0.63	2.41E-04	-0.02	8.43E-01	0.00	1.00E+00
FBgn0025549	unc-119	1224	-0.71	1.70E-13	-0.09	1.90E-01	0.00	1.00E+00
FBgn0025726	unc-13	8539	-0.16	3.82E-01	3.08	1.68E-117	0.01	9.70E-01
FBgn0024184	unc-4	56	0.21	1.73E-01	-1.78	2.44E-04	0.01	1.00E+00
FBgn0263352	Unr	3986	-1.51	8.25E-12	-0.01	9.12E-01	0.00	1.00E+00
FBgn0030904	upd2	26	0.21	1.41E-01	2.98	6.12E-04	0.00	1.00E+00
FBgn0053542	upd3	34	0.01	9.69E-01	2.77	2.35E-07	0.01	1.00E+00
FBgn0034245	UQCR-6.4	522	0.83	2.12E-05	0.02	8.90E-01	0.00	1.00E+00

FBgn0038271	UQCR-C1	4523	0.84	1.29E-13	0.03	7.62E-01	0.00	1.00E+00
FBgn0036728	UQCR-Q	1447	1.88	3.34E-40	0.00	9.79E-01	0.00	1.00E+00
FBgn0033428	Urod	1510	0.64	3.48E-06	0.02	8.25E-01	0.00	1.00E+00
FBgn0260749	Utx	1479	-0.75	1.33E-17	-0.06	4.40E-01	0.00	1.00E+00
FBgn0050101	Vajk4	36	1.18	5.97E-04	0.14	2.34E-01	0.00	1.00E+00
FBgn0035942	ValRS-m	232	0.60	4.18E-04	-0.01	9.18E-01	0.00	1.00E+00
FBgn0029687	Vap33	7747	-0.78	2.49E-07	0.01	9.16E-01	0.00	1.00E+00
FBgn0053200	VepD	48	-1.91	1.02E-06	0.11	3.01E-01	0.00	1.00E+00
FBgn0262524	ver	296	-0.22	2.25E-01	2.11	4.76E-65	0.00	1.00E+00
FBgn0261341	verm	9505	0.62	9.38E-04	-0.13	1.70E-01	0.00	1.00E+00
FBgn0033911	VGAT	134	-0.09	7.49E-01	0.64	1.29E-03	-0.63	6.34E-03
FBgn0267975	vib	1469	-1.04	1.87E-26	0.04	6.69E-01	-0.01	1.00E+00
FBgn0024183	vig	3544	-0.78	1.04E-08	0.01	9.06E-01	0.00	1.00E+00
FBgn0259978	vlc	2238	-0.62	3.56E-08	-0.03	7.54E-01	0.00	1.00E+00
FBgn0052350	Vps11	1027	-0.65	2.49E-03	0.00	9.72E-01	0.00	1.00E+00
FBgn0260987	vtd	2064	-0.95	1.33E-21	-0.06	4.50E-01	-0.01	1.00E+00
FBgn0266848	wap	3147	-0.66	1.92E-08	-0.03	7.45E-01	0.00	1.00E+00
FBgn0027492	wdb	5970	-0.84	1.30E-11	0.01	9.22E-01	0.00	1.00E+00
FBgn0027499	wde	2735	-0.89	1.99E-07	0.02	8.39E-01	0.00	1.00E+00
FBgn0011739	wts	1411	-0.66	9.62E-07	0.02	8.38E-01	0.00	1.00E+00
FBgn0261113	Xrp1	10923	-0.55	6.59E-06	1.08	1.69E-26	0.11	1.21E-01
FBgn0041711	yellow-e	215	0.87	5.43E-05	-0.03	7.39E-01	0.00	1.00E+00
FBgn0041710	yellow-f	83	-0.23	3.71E-01	-0.62	3.15E-02	-0.05	5.27E-01
FBgn0039896	yellow-h	56	0.51	4.40E-02	2.46	1.25E-10	-0.01	1.00E+00
FBgn0040060	yip7	41	-0.04	6.60E-01	-0.97	3.20E-02	-0.02	9.62E-01
FBgn0039261	Ythdf	2954	-0.66	9.40E-09	-0.04	6.25E-01	0.00	1.00E+00
FBgn0004606	zfh1	688	-0.13	5.56E-01	-0.63	2.67E-04	0.00	1.00E+00
FBgn0266709	Zmynd10	117	-1.05	1.89E-06	-0.13	2.22E-01	0.00	1.00E+00
FBgn0011642	Zyx	2299	-1.25	1.45E-35	0.00	9.77E-01	0.00	1.00E+00
