# Supplementary material for: Single amino-acid mutation in a Drosoph ila melanogaster ribosomal protein: An insight in uL11 transcriptional activity
Source: PLoS One. 2022 Aug 18;17(8):e0273198. doi: 10.1371/journal.pone.0273198 (PMC9387862; doi:10.1371/journal.pone.0273198)
Supplement: S4 Table — CC: Cellular Component; BP: Biological Process; MF: Molecular Function. (PDF) [file pone.0273198.s009.pdf]

Expression in corto^{L1}/corto^{420}		GO term	Count	p-value	Benjamini
up-regulated	BP-GO:0002181	cytoplasmic translation	66	6.90E-72	4.20E-69
up-regulated	CC-GO:0005840	ribosome	61	1.00E-61	2.00E-59
up-regulated	MF-GO:0003735	structural constituent of ribosome	82	5.40E-47	1.70E-44
up-regulated	CC-GO:0022625	cytosolic large ribosomal subunit	38	3.90E-39	3.90E-37
up-regulated	CC-GO:0005840	cytosolic small ribosomal subunit	28	4.10E-28	2.70E-26
up-regulated	CC-GO:0005840	cytosolic ribosome	41	2.10E-23	1.00E-21
up-regulated	BP-GO:0006412	translation	74	2.80E-22	8.50E-20
up-regulated	CC-GO:0005840	mitochondrial respiratory chain complex I	15	6.40E-10	1.80E-08
up-regulated	MF-GO:0003954	NADH dehydrogenase activity	10	1.50E-06	1.60E-04
up-regulated	BP-GO:0002181	mitochondrial electron transport, NADH to ubiquinone	9	2.80E-05	2.90E-03
up-regulated	MF-GO:0003954	NADH dehydrogenase (ubiquinone) activity	8	4.10E-04	1.80E-02
up-regulated	CC-GO:0005751	mitochondrial respiratory chain complex IV	6	1.10E-04	1.70E-03
up-regulated	MF-GO:0004129	cytochrome-c oxidase activity	7	1.80E-04	1.20E-02
up-regulated	BP-GO:0006123	mitochondrial electron transport, cytochrome c to oxygen	5	5.40E-04	3.70E-02
up-regulated	CC-GO:0005750	mitochondrial respiratory chain complex III	5	5.10E-04	7.20E-03
up-regulated	MF-GO:0008121	ubiquinol-cytochrome-c reductase activity	5	7.10E-04	2.50E-02
up-regulated	BP-GO:0006122	mitochondrial electron transport, ubiquinol to cytochrome c	5	1.80E-03	9.00E-02
up-regulated	BP-GO:0006465	signal peptide processing	5	8.40E-05	7.40E-03
up-regulated	CC-GO:0005787	signal peptidase complex	3	7.90E-03	9.20E-02
up-regulated	BP-GO:1902600	proton transport	7	2.00E-04	1.50E-02
up-regulated	MF-GO:0046933	proton-transporting ATP synthase activity, rotational mechanism	6	3.80E-04	1.80E-02
up-regulated	BP-GO:0040003	chitin-based cuticle development	15	1.10E-03	6.70E-02
up-regulated	MF-GO:0009055	electron carrier activity	8	2.20E-03	7.00E-02
down-regulated	BP-GO:0006465	protein phosphorylation	30	7.60E-09	9.30E-06
down-regulated	MF-GO:0004674	protein serine/threonine kinase activity	23	1.20E-08	2.10E-06
down-regulated	MF-GO:0004672	protein kinase activity	19	4.00E-06	4.60E-04
down-regulated	MF-GO:0005524	ATP binding	45	2.30E-04	1.30E-02
down-regulated	BP-GO:0045475	locomotor rhythm	9	9.60E-04	6.20E-02
down-regulated	CC-GO:0005834	heterotrimeric G-protein complex	5	7.10E-04	3.00E-02
down-regulated	MF-GO:0004871	signal transducer activity	7	1.40E-03	5.30E-02
down-regulated	MF-GO:0031683	G-protein beta/gamma-subunit complex binding	4	1.90E-03	5.50E-02
down-regulated	BP-GO:0030307	positive regulation of cell growth	7	2.70E-04	2.40E-02

Expression in uL11^{K3A}		GO term	Count	p-value	Benjamini
up-regulated	BP~GO:0006749	glutathione metabolic process	9	3.10E-07	1.30E-04
up-regulated	MF~GO:0004364	glutathione transferase activity	8	5.00E-06	8.90E-04
up-regulated	MF~GO:0004602	glutathione peroxidase activity	7	8.10E-06	8.90E-04
up-regulated	BP~GO:0000723	telomere maintenance	6	1.30E-05	2.60E-03
up-regulated	BP~GO:0006310	DNA recombination	5	6.70E-04	7.60E-02
down-regulated	MF~GO:0043565	sequence-specific DNA binding	13	2.90E-05	4.10E-03
down-regulated	BP~GO:0006355	regulation of transcription, DNA-templated	14	1.20E-04	9.30E-03
down-regulated	BP~GO:0006351	transcription, DNA-templated	13	2.60E-04	1.50E-02
down-regulated	MF~GO:0003700	transcription factor activity, sequence-specific DNA binding	11	8.50E-04	4.40E-02
down-regulated	MF~GO:0004035	alkaline phosphatase activity	4	9.30E-04	4.40E-02
