# Supplementary material for: Single amino-acid mutation in a Drosoph ila melanogaster ribosomal protein: An insight in uL11 transcriptional activity
Source: PLoS One. 2022 Aug 18;17(8):e0273198. doi: 10.1371/journal.pone.0273198 (PMC9387862; doi:10.1371/journal.pone.0273198)

Figure 2, top:

Exposure 2’30”” (ECL)

Primary antibodies: α-uL11K3me3 1/6000; α-tubuline 1/10000

Secondary antibodies: α-rabbit IgG 1/20000; α-rat IgG 1/10000

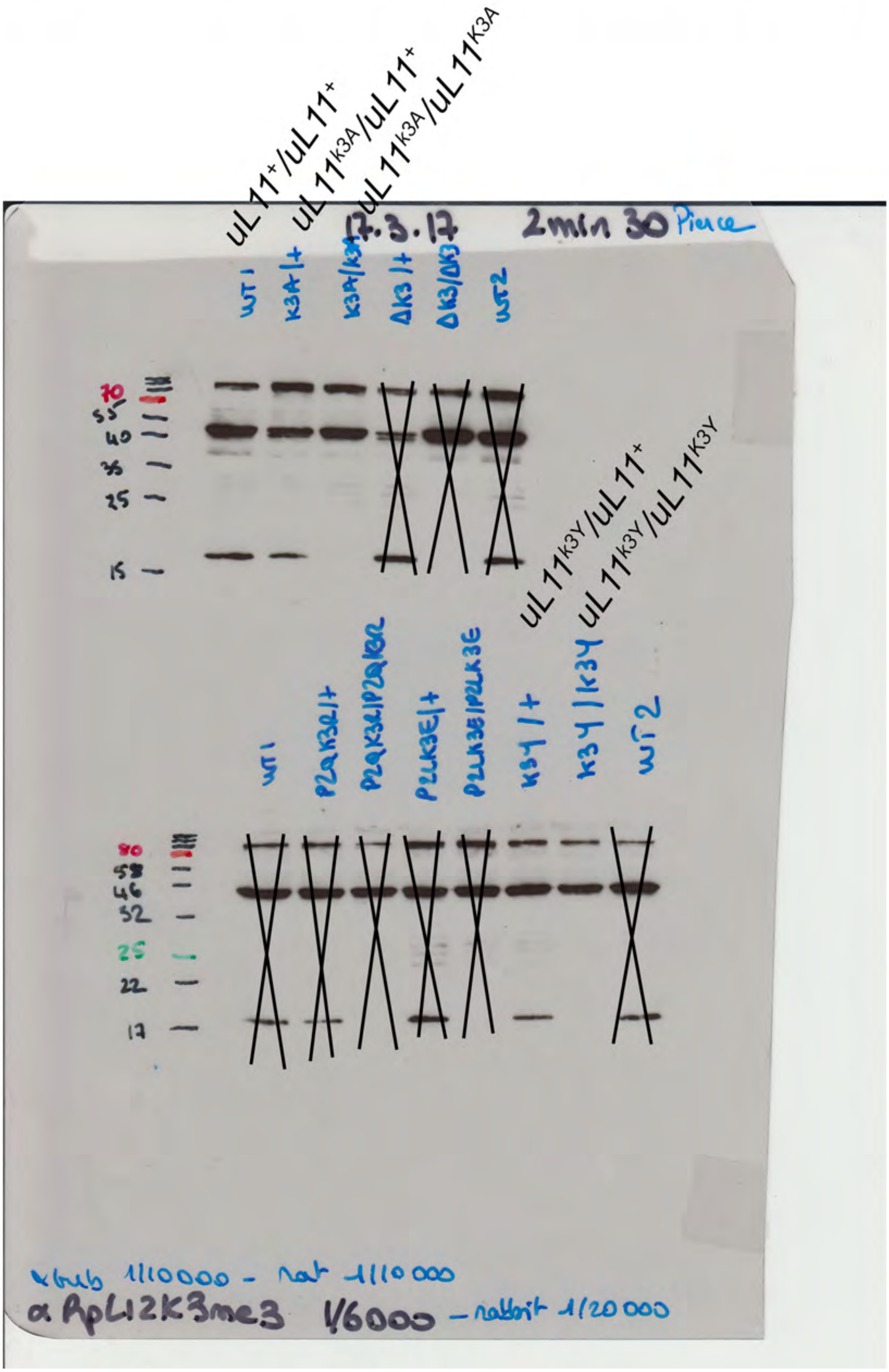

Figure 2, bottom:

Exposure 2’30”

Primary antibodies: α-uL11 1/1000

Secondary antibodies: α-goat IgG 1/10000

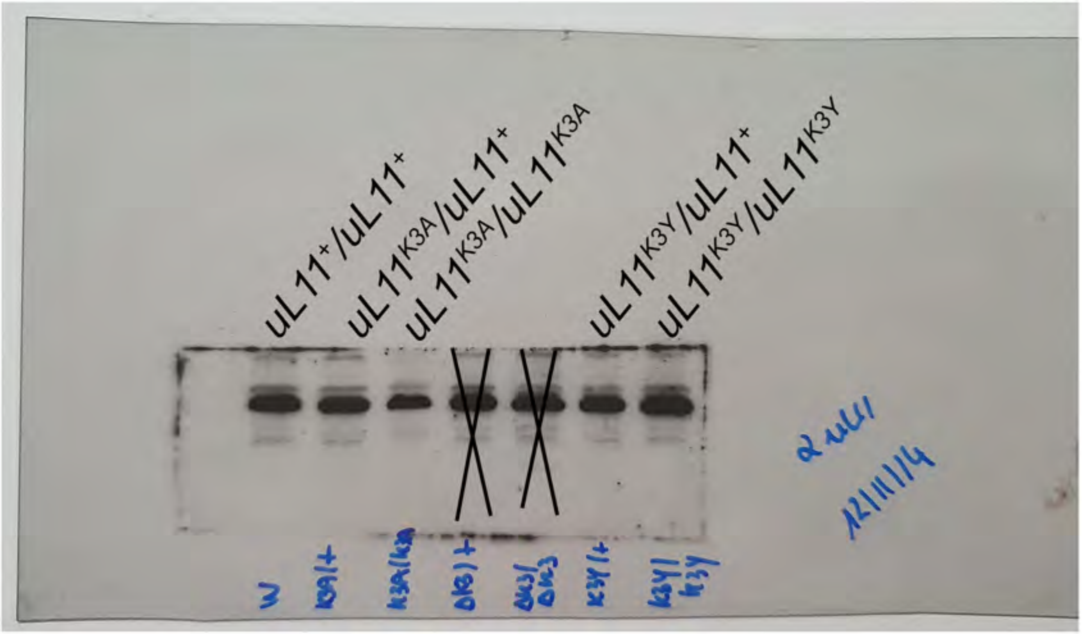

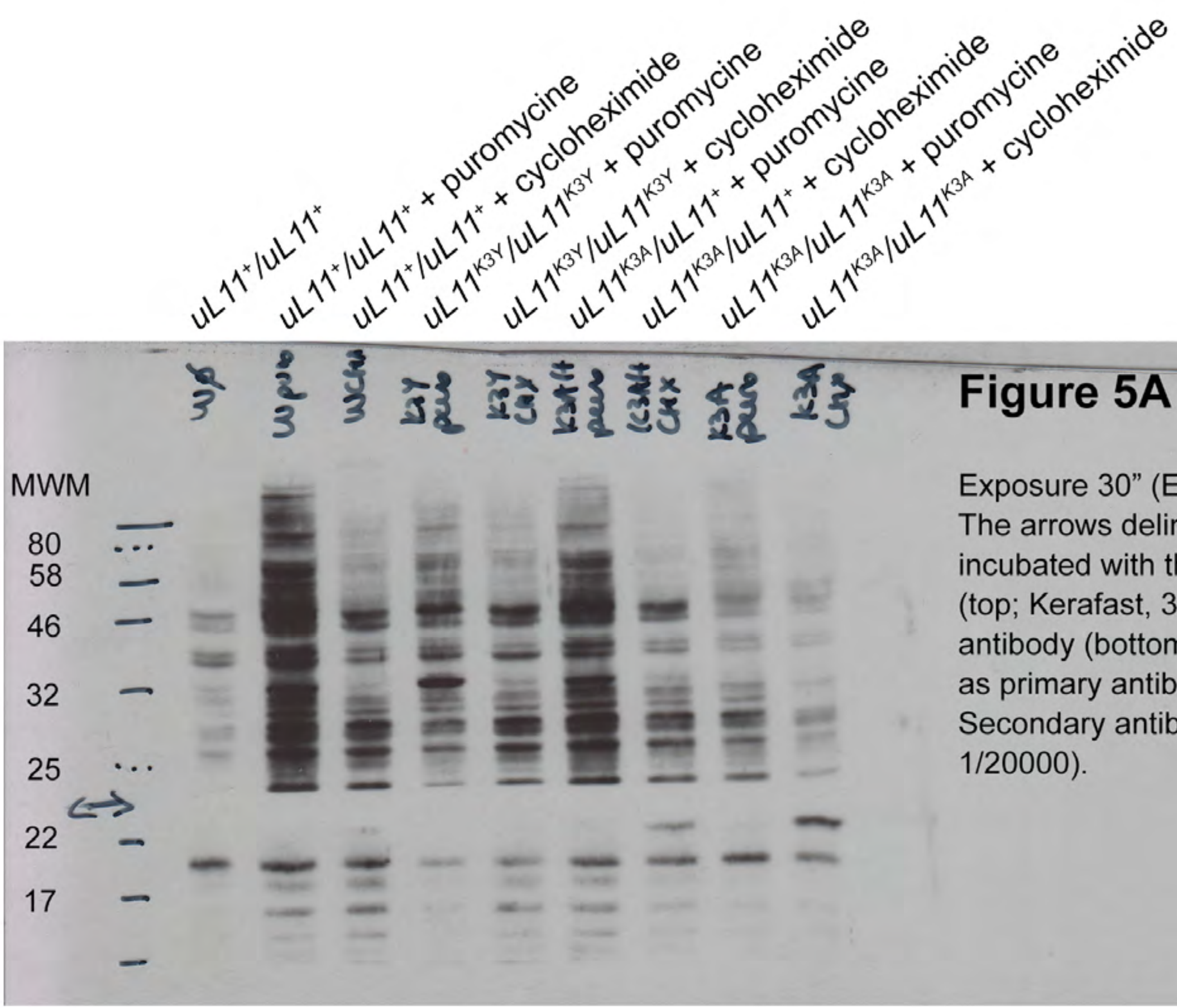

Figure 5A

Exposure 30" (ECL).
The arrows delimit the part of the Western blot incubated with the mouse anti-puromycin antibody (top; Kerafast, 3RH11; 1/500) or the mouse anti-H3 antibody (bottom) (Diagenode; C15200011; 1/1000). as primary antibodies.
Secondary antibodies: anti-mouse (Sigma; NA931; 1/20000).

Figure 5

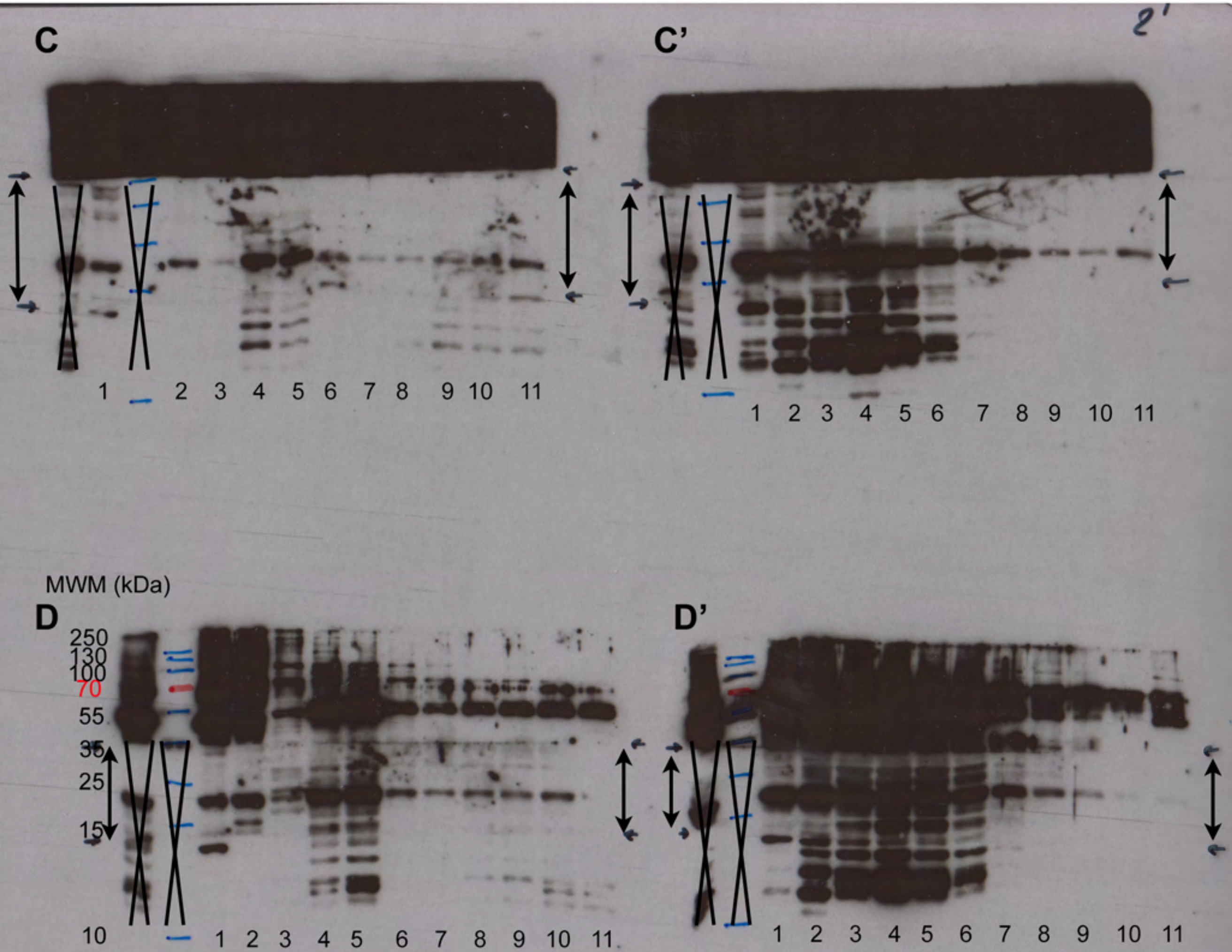

Exposure 2' (ECL).
The arrows delimit the part of the Western blot incubated with the anti-HA primary antibody (Sigma F2411, 1/1,000). Secondary antibody: anti-mouse (Sigma NA931; 1/20000).

Figure 6A

IP Flag-CortoCD / co-IP uL11-Myc

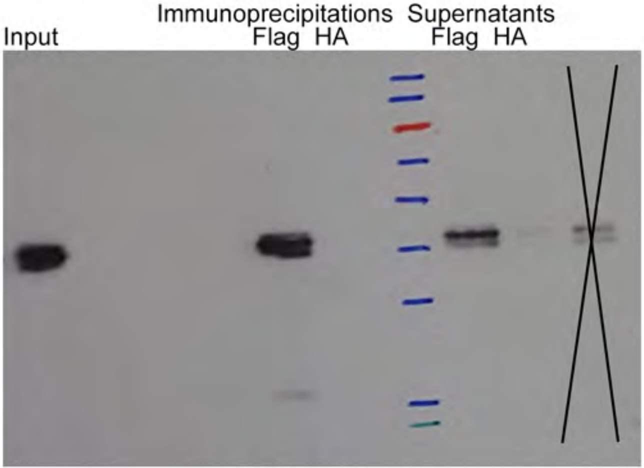

anti-Flag antibody

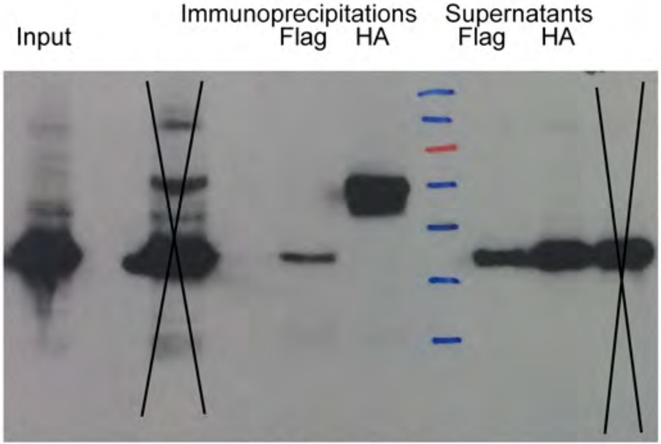

anti-Myc antibody

Figure 6B

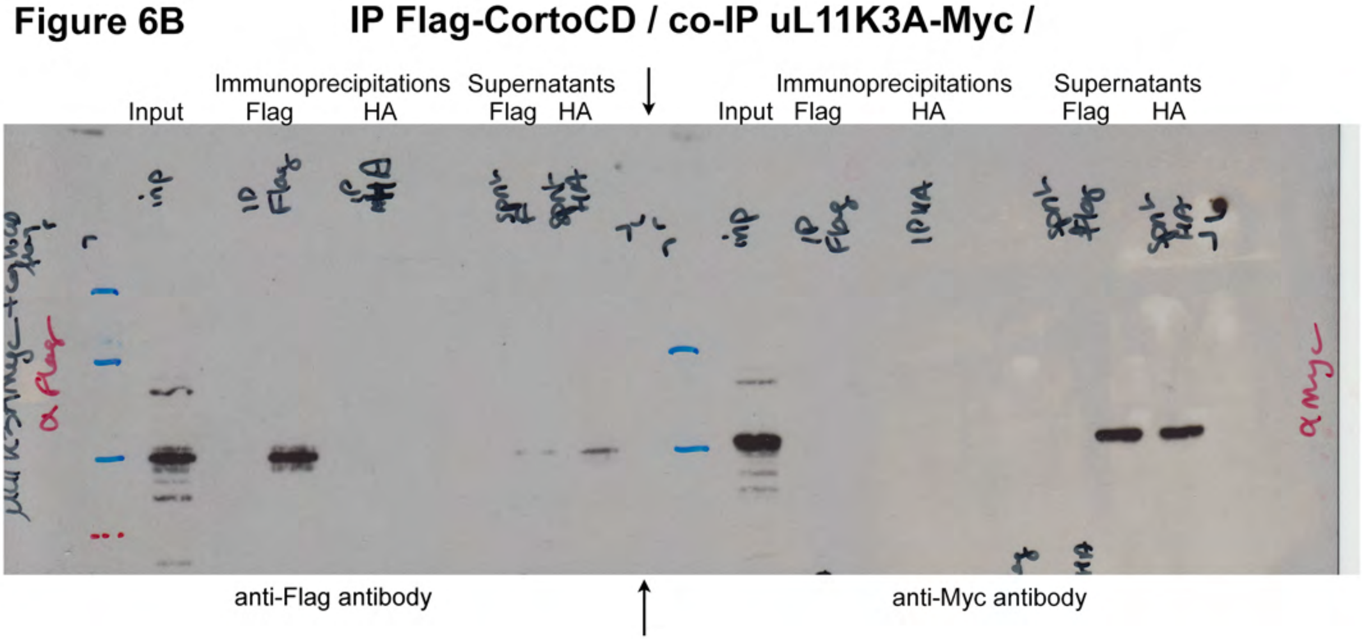
IP Flag-CortoCD / co-IP uL11K3A-Myc /
Immunoprecipitations
Supernatants
Input
Flag
HA
Flag
HA
Immunoprecipitations
Supernatants
Input
Flag
HA
Flag
HA
anti-Flag antibody
anti-Myc antibody

Figure 6C

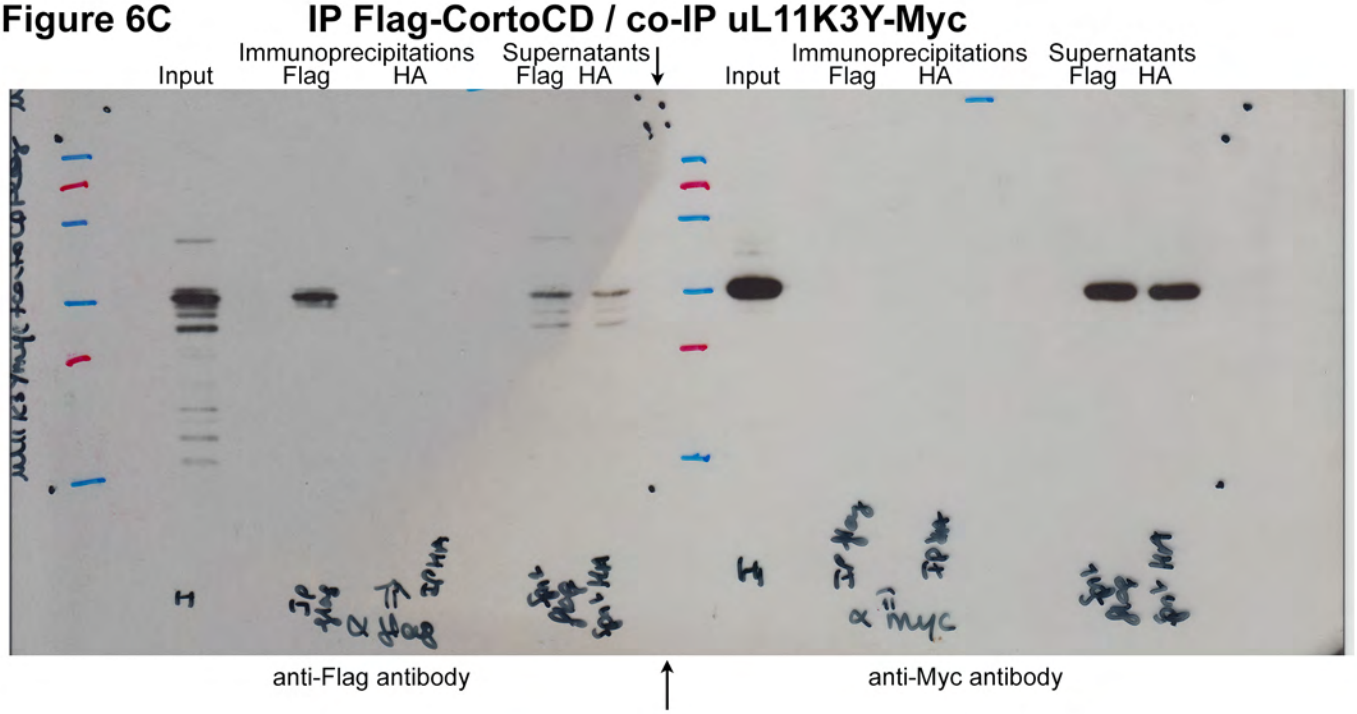
IP Flag-CortoCD / co-IP uL11K3Y-Myc
Immunoprecipitations
Supernatants
Input
Flag
HA
Flag
HA
Immunoprecipitations
Supernatants
Input
Flag
HA
Flag
HA
anti-Flag antibody
anti-Myc antibody

Figure 7A et 7C

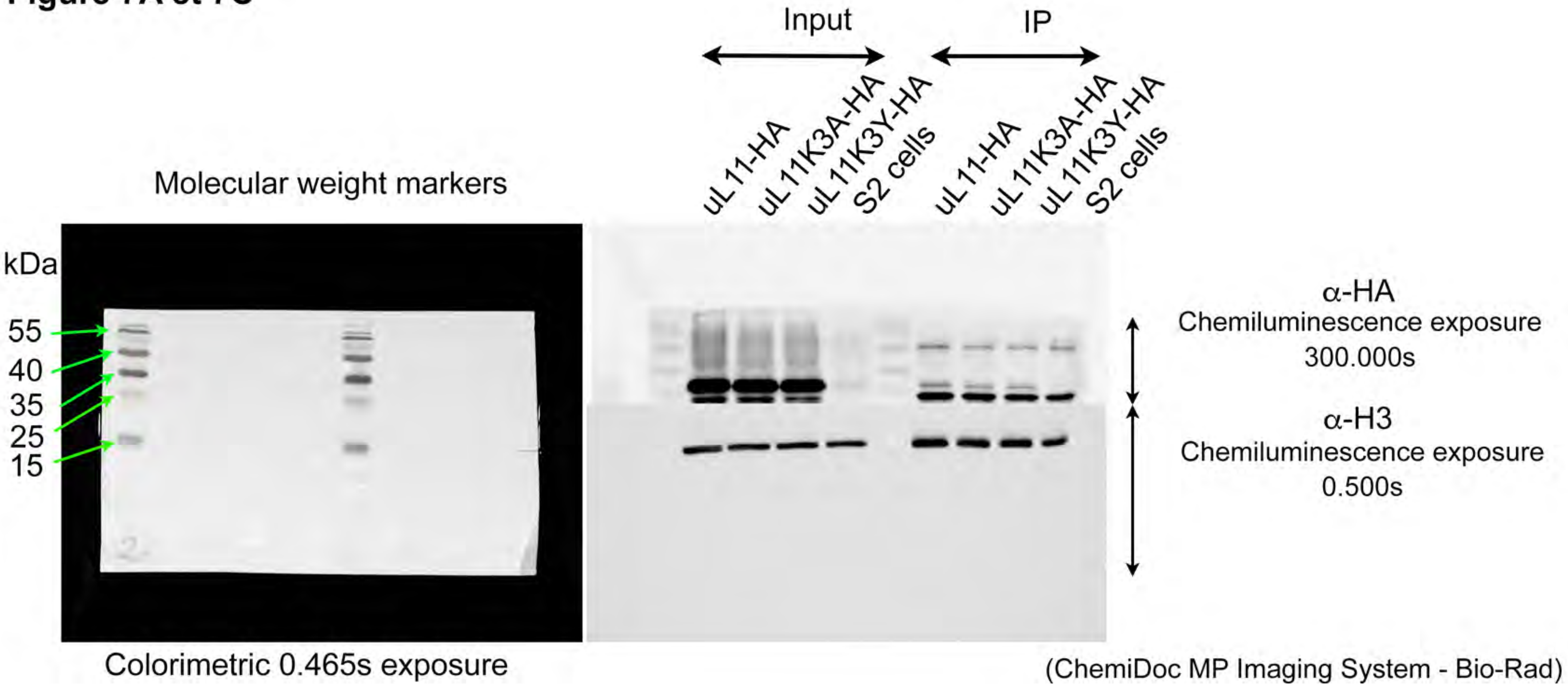

Figure 7B

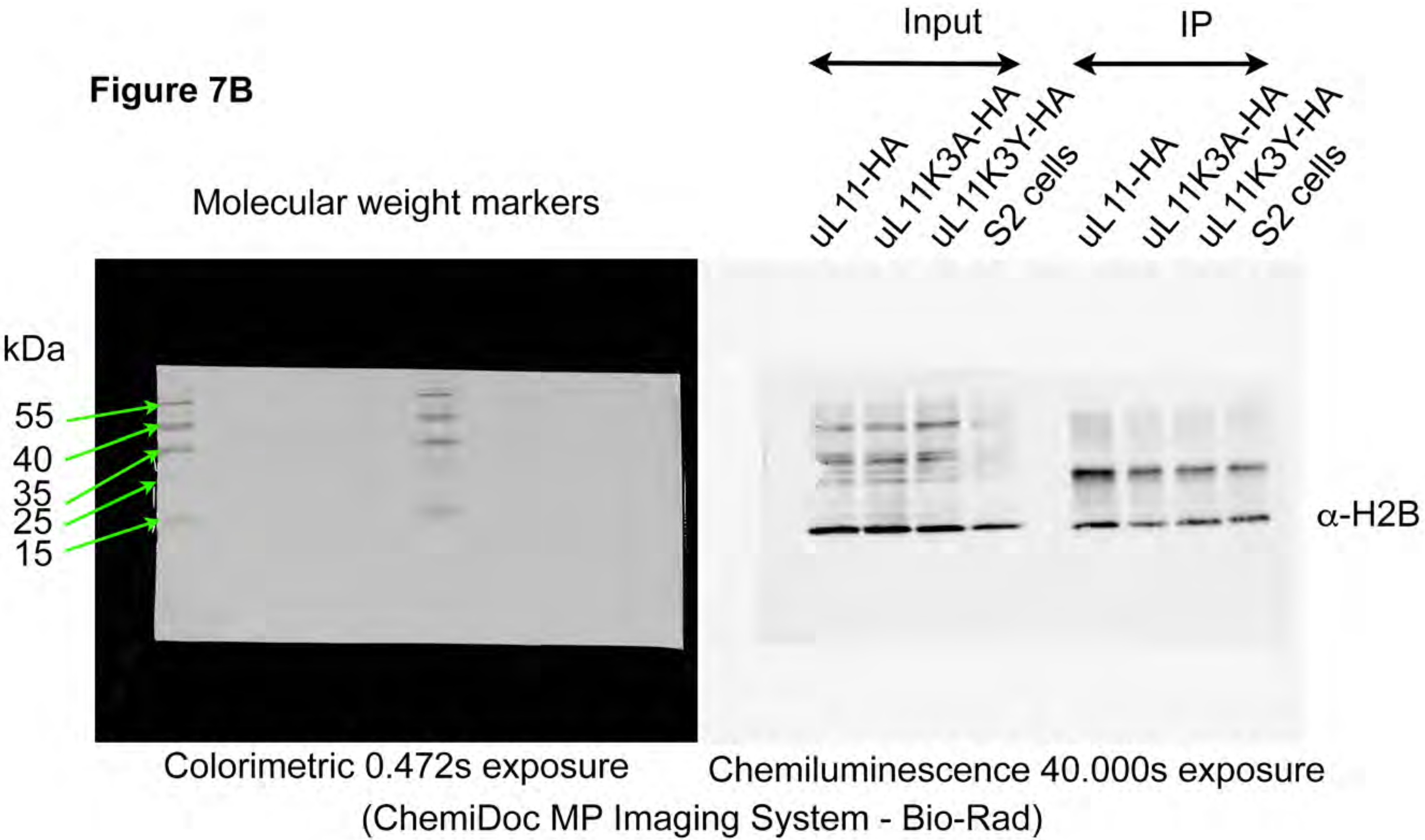

Input
IP
uL11-HA
uL11K3A-HA
uL11K3Y-HA
S2 cells
uL11-HA
uL11K3A-HA
uL11K3Y-HA
S2 cells
Molecular weight markers
kDa
55
40
35
25
15
α-H2B
Colorimetric 0.472s exposure
Chemiluminescence 40.000s exposure
(ChemiDoc MP Imaging System - Bio-Rad)

Supplementary Figure 1

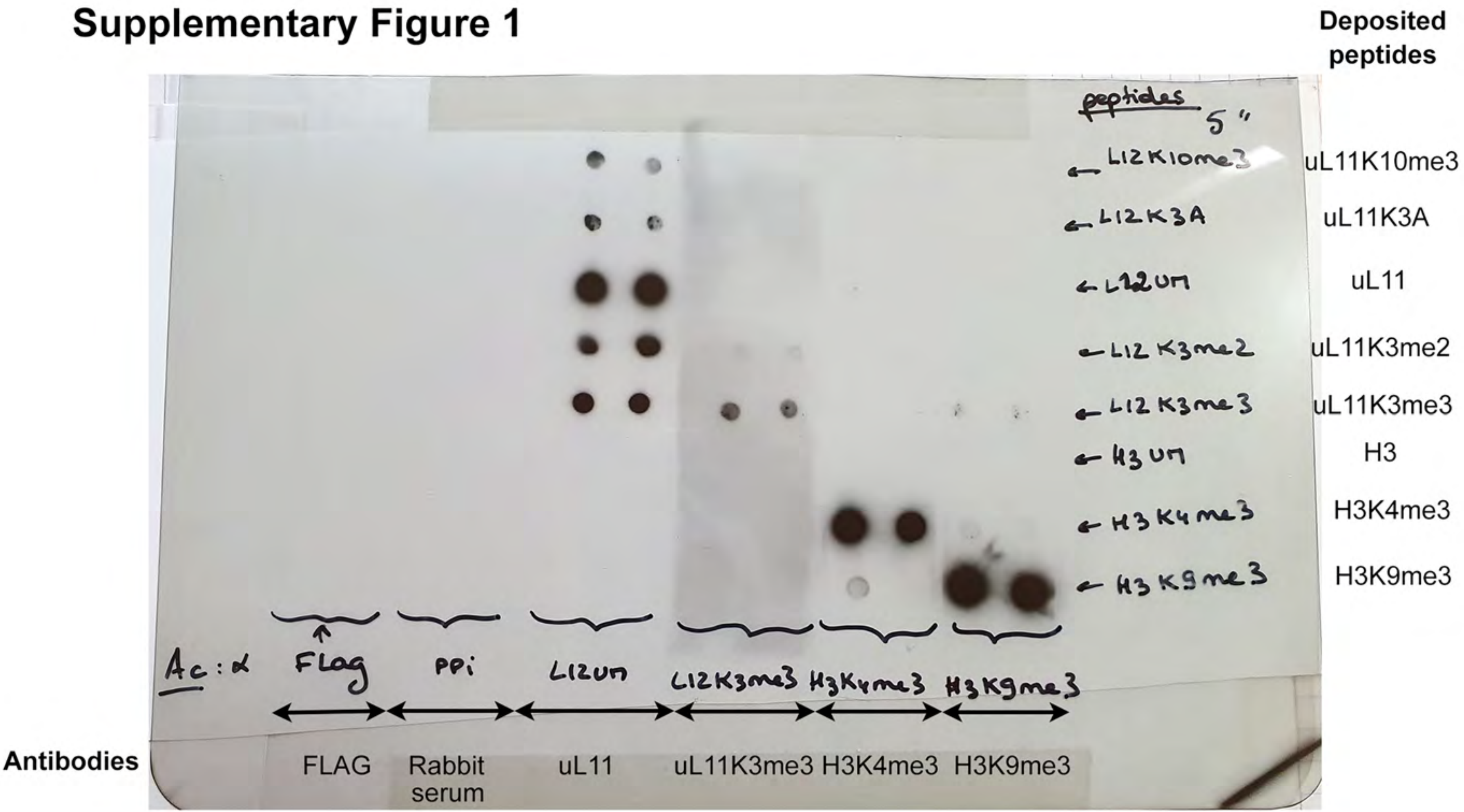

Supplement: S1 Raw images — (PDF) [file pone.0273198.s010.pdf]
